# AI-Validated Brain Targeted mRNA Lipid Nanoparticles with Neuronal Tropism

**DOI:** 10.1021/acsnano.4c15013

**Published:** 2025-09-16

**Authors:** Mor Sela, Gal Chen, Haim Kadosh, Tomer Kagan, Raneen Nicola, Sally Turutov, Yuval Richtman, Lin Zhige, Mia R. Albalak Menasherov, Shaked Kagan, Tzur Schroeder, Patricia Mora-Raimundo, Reaam Kablan, Egor Egorov, Anas Odeh, Tasneem Abu-Raiya, Inbal Ionita, Inbar Freilich, Galoz Kaneti, Ibrahim Knani, Yehuda Arav, Yael Leichtmann-Bardoogo, Keshet Tadmor, Jeny Shklover, Tommaso Patriarchi, Dganit Danino, Peleg Hasson, Uri Ashery, Amit Zeisel, Ben M. Maoz, Tal Laviv, Kira Radinsky, Avi Schroeder

**Affiliations:** † The Louis Family Laboratory for Targeted Drug Delivery and Personalized Medicine Technologies, Department of Chemical Engineering, 26747 Technion—Israel Institute of Technology, Haifa 3200003, Israel; ‡ The Interdisciplinary Program for Biotechnology, 26747 Technion—Israel Institute of Technology, Haifa 3200003, Israel; § Department of Physiology and Pharmacology, Gray Faculty of Medical and Health Sciences, 26745 Tel Aviv University, Tel Aviv 6997801, Israel; ∥ Department of Biomedical Engineering, Engineering Faculty, 26745 Tel Aviv University, Tel Aviv 6997801, Israel; ⊥ Faculty of Computer Sciences, 26747 Technion—Israel Institute of Technology, Haifa 3200003, Israel; ☆ Faculty of Biotechnology and Food Engineering, 26747 Technion—Israel Institute of Technology, Haifa 3200003, Israel; ∇ The Norman Seiden Multidisciplinary Program for Nanoscience and Nanotechnology, 26747 Technion—Israel Institute of Technology, Haifa 3200003, Israel; ○ Department of Genetics and Developmental Biology, The Rappaport Faculty of Medicine and Research Institute, 26747 Technion—Israel Institute of Technology, Haifa, 3200003 Israel; ◆ CryoEM Laboratory of Soft Matter, Department of Biotechnology and Food Engineering, 26747 Technion—Israel Institute of Technology, Haifa 3200003, Israel; ¶ Department of Applied Mathematics, 54623 Israel Institute for Biological Research, Ness-Ziona 7410001, Israel; ■ Sagol School of Neuroscience, 26745 Tel Aviv University, Tel Aviv 6997801, Israel; △ Institute of Pharmacology and Toxicology, University of Zürich, CH-8006 Zürich, Switzerland; ▼ Neuroscience Center Zürich (ZNZ), University of Zürich, CH-8006 Zürich, Switzerland; ★ bCryo-EM and Self-Assembly Laboratory, Guangdong−Technion—Israel Institute of Technology, Shantou 515063, China; ● School of Neurobiology, Biochemistry, Biophysics, Life Sciences Faculty, 26745 Tel Aviv University, Tel Aviv 6997801, Israel; ▲ Drimmer-Fischler Family Stem Cell Core Laboratory for Regenerative Medicine, 26745 Tel Aviv University, Tel Aviv 6997801, Israel; ⬡ Student at the Faculty of Medicine, The Rappaport Faculty of Medicine and Research Institute, 26747 Technion—Israel Institute of Technology, Haifa 3200003, Israel

**Keywords:** Brain Targeting, Lipid Nanoparticles, Gene Delivery, mRNA, Blood−Brain Barrier, Artificial Intelligence, Central Nervous System (CNS)

## Abstract

Targeting therapeutic nanoparticles to the brain poses a challenge due to the restrictive nature of the blood–brain barrier (BBB). Here we report the development of mRNA-loaded lipid nanoparticles (LNPs) functionalized with BBB-interacting small molecules, thereby enhancing brain delivery and gene expression. Screening brain-targeted mRNA-LNPs in central nervous system (CNS) *in vitro* models and through intravenous administration in mice demonstrated that acetylcholine-conjugated LNPs achieved superior brain tropism and gene expression, outperforming LNP modifications with nicotine, glucose, memantine, cocaine, tryptophan, and other small molecules. An artificial intelligence (AI)-based model designed to predict the BBB permeability of small-molecule ligands showed strong alignment with our experimental results, providing *in vivo* validation of its predictive capacity. Cell-specific biodistribution analysis in Cre-reporter Ai9 mice showed that acetylcholine-functionalized LNPs preferentially transfected neurons and astrocytes following either intravenous or intracerebral administration. Mechanistic studies suggest that acetylcholine-LNP uptake is mediated by the functional engagement of acetylcholine receptors (AchRs) followed by endocytosis, which synergistically enhances intracellular mRNA delivery. Moreover, acetylcholine-LNPs successfully crossed a human BBB-on-a-chip model, enabling transgene expression in human iPSC-derived neurons. Their effective penetration and transfection in human brain organoids further support their potential activity in human-based systems. These findings establish a predictive and modular framework for engineering CNS-targeted LNPs, advancing precision gene delivery for brain disorders.

## Graphical abstract

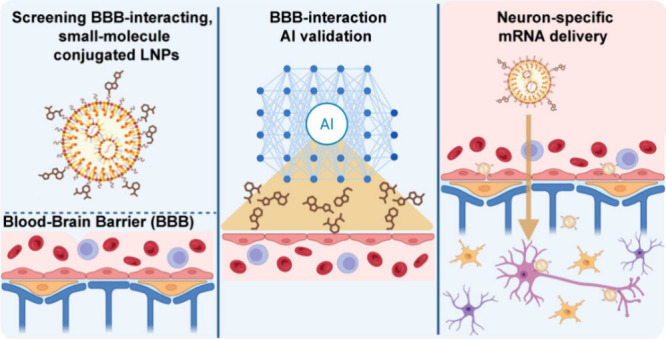

## Introduction

The delivery of therapeutic agents to the brain remains a formidable challenge due to the protective nature of the blood–brain barrier (BBB). This barrier, composed of tightly packed endothelial cells, pericytes, and astrocytes, restricts the delivery of most molecules and nanoparticles into the brain.^1^ Overcoming this barrier is crucial for treating neurological and degenerative disorders, such as Parkinson’s disease and Alzheimer’s disease.^2^

Nucleic acid therapeutics, such as mRNA^3^ and small interfering RNA (siRNA),^4–8^ hold promise for addressing brain disorders, enabling modulation of gene expression, replacement of defective proteins, or silencing harmful genes.^9,10^

Lipid nanoparticles (LNPs) are promising delivery systems for nucleic acid therapeutics due to their biocompatibility and ability to encapsulate and deliver RNA molecules intracellularly.^11–14^ The adaptable structure of LNPs permits their customization for targeted delivery to specific organs and cell types following systemic or local administration.^15–17^ A significant advancement in LNP technology is the incorporation of targeting moieties on their surface.^18–20^ More recently, landmark studies have demonstrated that targeted LNPs can efficiently deliver mRNA across the BBB, underscoring both the therapeutic potential and the pressing need for developing genetic delivery platforms to the CNS.^21–23^

Small-molecule PEG-lipid conjugates offer a chemically modular tool for improving LNP tropism toward target organs, including the brain. Integrating artificial intelligence (AI)-based models into the design process can further enhance this approach by enabling more efficient screening, minimizing reliance on animal models, and accelerating discovery speed.^24^

Here, we synthesized a library of brain-targeted (BT) mRNA-loaded LNPs and show that integrating PEG-lipids, which are conjugated to BBB-interacting small molecules at their distal ends, into standard LNP formulations alters the *in vivo* RNA biodistribution and expression profile. This modification facilitates brain-specific gene delivery and selectively drives tropism toward neurons (Figure 1A). The molecules we tested, including derivatives of glucose (d-glucuronic acid),^25^ methylphenidate (ritalinic acid),^26^ memantine (3,5-dimethyladamantane-1-carboxylic acid),^27^ acetylcholine (carbamylcholine chloride),^28^ cocaine (benzoylecgonine),^29^ nicotine (*trans*-4-cotininecarboxylic acid),^30^ norepinephrine (droxidopa),^31^ and tryptophan,^32^ all of which cross the BBB in their unconjugated form. We hypothesized that synthetic PEG-lipids attached to these moieties would retain their capability to cross the BBB and improve LNP-mediated brain delivery.

**Figure 1:**
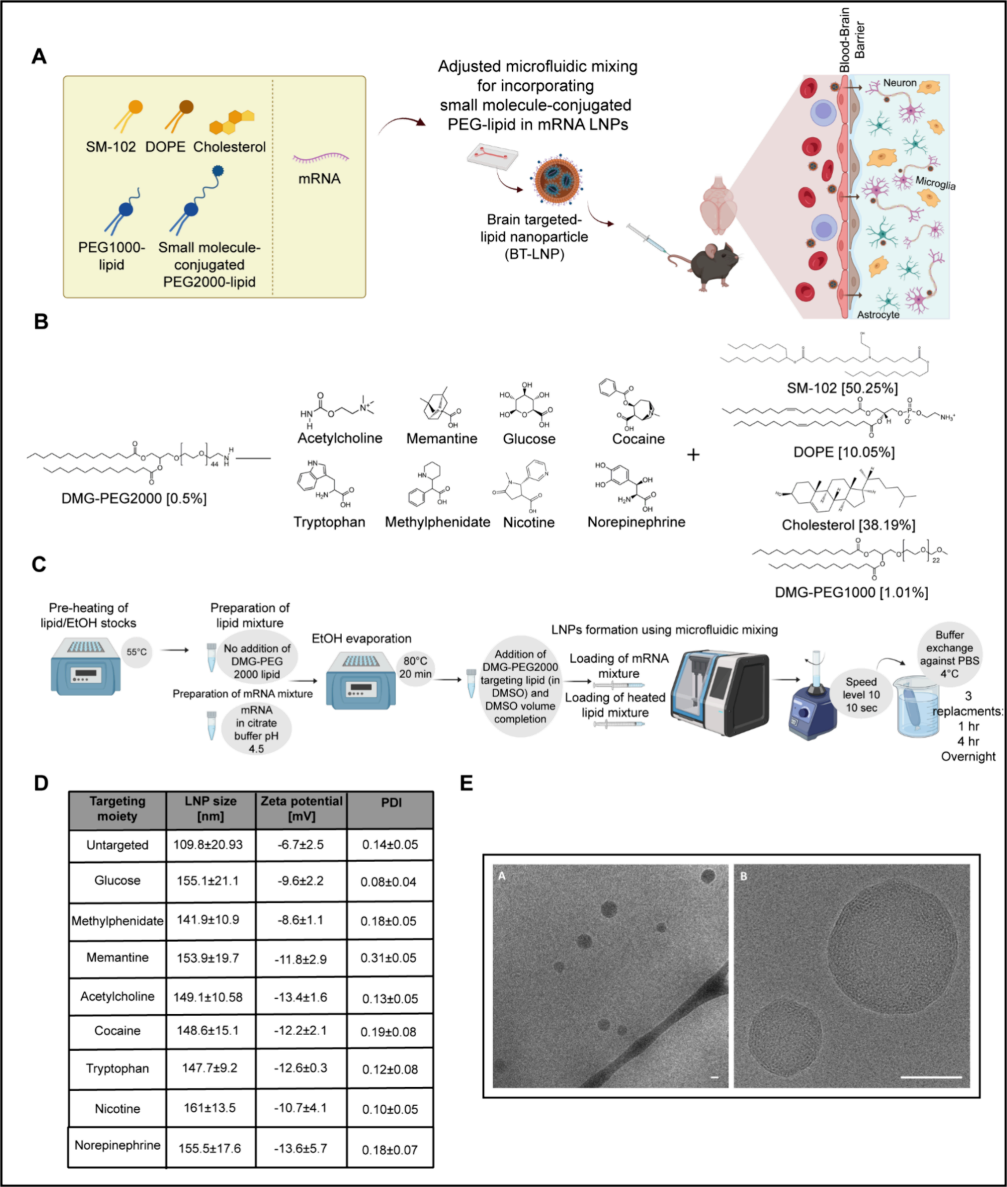
**Engineering a brain-targeted (BT) mRNA-loaded LNP library.** (A) mRNA LNPs were formulated with a blood–brain barrier (BBB)-interacting small molecule-conjugated PEG-lipid using optimized microfluidic mixing to enable BBB crossing, after intravenous administration, and subsequent selective brain-cell transfection, including neurons. (B) BT-LNPs lipid phase composition [mol %]. DMG-PEG2000 was conjugated to derivatives of the presented small molecules, with tryptophan used in its original form. (C) The microfluidic mixing-based production method of LNPs was adjusted to facilitate the integration of small-molecule-conjugated PEG lipid. DMSO was used instead of EtOH to improve solubility, and process temperatures were optimized for best performance. (D) Physicochemical properties of BT-LNP library. Size, polydispersity index (PDI) (*n* ≥ 8 independent groups), and zeta potential (*n* = 3). Data are shown as mean ± SD. (E) Cryo-TEM imaging confirmed the uniformity, repeatability, and size distribution of BT-LNPs. Glucose-LNPs were used as a representative formulation for imaging. Scale bar = 100 nm. Illustrations were created in BioRender [Shklover, J. (2025) https://BioRender.com/fmeu0vq].

As part of optimizing the targeted LNPs and their preparation process, we examined parameters such as PEG molar percentage, mRNA concentration, ionizable lipid-to-mRNA weight ratio, and solvent choice (see Methods and Experimental Procedures and Table S1). Our optimized methodology enables the reproducible synthesis of functional mRNA-loaded LNPs conjugated to nonsoluble small-molecule moieties.

Luminescence-based assays performed in both brain endothelial and neuron-like cell cultures, as well as *in vivo* following systemic mRNA delivery in mice, identified acetylcholine-conjugated LNPs as a top-performing candidate in terms of transfection efficiency and brain-specific expression. In parallel, we employed a graph-based AI model to predict BBB interaction of the ligands. We observed strong alignment with our *in vivo* results, underscoring the utility of computational tools in guiding nanoparticle design.

We further evaluated the acetylcholine formulation using a human iPSC-derived transwell BBB model, demonstrating the ability to transfect neurons after crossing an intact endothelial barrier. Application to cortical brain organoids confirmed its capacity to penetrate complex neural tissue and mediate transgene expression. Mechanistic studies revealed that acetylcholine-LNPs engage acetylcholine receptors and enter cells, contributing significantly to their enhanced mRNA transfection compared to untargeted LNPs.

Finally, flow cytometry and *ex vivo* imaging were used to examine systemic LNPs administration, confirming successful transgene expression within the brain. Advanced image analysis performed after direct intracerebral injection demonstrated the high transfection specificity of acetylcholine-LNPs toward astrocytes and neurons.

Together, this work establishes a predictive and modular framework for engineering scalable, CNS-targeted gene therapies for precision treatment of brain disorders, and emphasizes the importance of computational tools in guiding nanoparticle design.

## Results and Discussion

### Design and Synthesis of BT-LNP Library

LNPs consisted of the ionizable lipid SM-102, an FDA-approved lipid, used in COVID-19 vaccines,^33^ 1,2-dioleoyl-*sn*-glycero-3-phosphoethanolamine (DOPE), cholesterol, and modified and unmodified PEG-lipid mixture.

To facilitate brain targeting, a set of BT PEG-lipids (1,2-dimyristoyl-*rac*-glycero-3-aminepolyethylene glycol-2000 (DMG-PEG2000-NH_2_)) modified with BBB-interacting small molecules was synthesized. The small molecule targeting approach enables a modular preparation process, supports chemical and physical stability, and a cost-effective, large-scale production.

PEG2000 is commonly used for ligand conjugation, allowing the targeting moieties to extend beyond the nanoparticle surface for effective receptor binding. However, excessive PEG density can hinder uptake due to steric hindrance.^34^ Combining PEG2000-ligand conjugates with shorter PEG chains, such as PEG1000, has been shown to enhance targeting and reduce these steric obstructions.^35,36^

We created a series of eight PEG-lipids (DMG-PEG2000-small molecule moiety) conjugated to derivatives of the following small molecules: glucose, methylphenidate, memantine, acetylcholine, cocaine, tryptophan, nicotine, and norepinephrine (Compound Spectra S1–S9). These molecules were selected due to their potential to enhance interaction with brain endothelial cells, key components of the BBB,^37^ and neurons. For example, glucose targets glucose transporters on the BBB and neurons;^38^ methylphenidate interacts with dopamine transporters;^39,40^ memantine binds *N*-methyl-d-aspartate (NMDA) receptors,^41,42^ and acetylcholine stimulates cholinergic receptors.^43,44^ Likewise, cocaine binds dopamine and norepinephrine transporters, tryptophan interacts with serotonergic neurons via serotonin pathways,^45,46^ nicotine engages nicotinic acetylcholine receptors,^47^ and norepinephrine interacts with adrenergic receptors.^48^ An untargeted-LNP formulation was used as a reference for comparison (see Table S3).

The LNPs were formulated using microfluidic mixing^49^ of an organic phase containing ionizable lipid (SM-102; 50.25 mol %), a helper lipid (DOPE; 10.05 mol %), cholesterol (38.19 mol %), and PEG-lipids (DMG-PEG1000; 1.01 mol % and DMG-PEG2000-moiety; 0.5 mol %) (Figure 1B). For LNP production, ethanol is traditionally used as a solvent to dissolve lipids.^50^ However, our experiments show that ethanol poses solubility challenges, particularly for lipids linked to hydrophobic small molecules. DMSO was found to be a suitable alternative, overcoming these solubility issues and ensuring proper lipid integration into the LNP formulation.

Specifically, the lipid mixture was initially dissolved in ethanol, which was then evaporated at 80 °C to obtain a lipid powder. Following evaporation, DMSO was added, and the lipid mix was heated at 65 °C until the solution became clear and ready for loading into the microfluidic syringe. Importantly, after loading into the syringe, the solution underwent an additional heating step to ensure the solution remained clear and prepared for the microfluidic device. The primary consideration when using DMSO is that the solubility of the lipids is limited to 36 mg/mL.

For the mRNA phase, a standard aqueous buffer (with a maximum loading capacity of 94.78 μg mRNA/mL) was utilized (Figure 1C).

### Characterization of BT-LNPs

To validate consistent particle preparation, the physical properties of the formulated LNPs were measured (Figure 1D). Particle sizes ranged from 109.8 ± 20.93 nm to 161.0 ± 13.5 nm, with polydispersity index (PDI) ranging from 0.08 ± 0.04 to 0.31 ± 0.05, and negative zeta potentials from −6.7 ± 2.5 mV to −13.7 ± 5.7 mV, indicating that the addition of targeting moieties did not substantially impact the physical properties. All formulations achieved mRNA encapsulation efficiencies of approximately 90%, highlighting the efficiency of the encapsulation process regardless of the targeting moiety used (Table S2).

Cryogenic transmission electron microscopy (cryo-TEM) images of a representative formulation, glucose-LNP, confirmed the LNPs’ spherical morphology and uniform size distribution (Figure 1E).

Next, we assessed the colloidal and structural stability of a representative BT-LNP formulation (acetylcholine) under standard storage conditions. Specifically, we monitored the particle size, PDI, and mRNA encapsulation efficiency over 20 days at 4 and 25 °C (Figure S1). The untargeted LNP formulation stored at 4 °C was analyzed on days 1 and 21 to assess particle stability (Figure S2). We found that BT-LNPs maintain consistent mean diameters throughout the entire time course, without aggregation or degradation at both temperatures. Similarly, encapsulation efficiency remained stable, with only a minimal reduction (of ∼1.05%) observed at 25 °C. These findings suggest that the BT-LNP retains its structural integrity and mRNA-loading under the experimental conditions.

### Using AI to Predict Small Molecules That Will Target and Cross the BBB

AI models provide advantages in drug development, particularly by enabling the early prediction of pharmacological properties, toxicity, and therapeutic efficacy.^51^ Additionally, AI-driven approaches facilitate the prioritization of candidate compounds, reducing the number of animals used in preclinical trials, streamlining experimental design, and expediting the identification of promising therapeutic leads. As part of this study, we developed a neural network-based model to predict the passage of molecules through the BBB in both rats and humans. The model leverages a graph-based approach where molecules are represented as graphs and encoded into embeddings using a Graph Neural Network (GNN). Additionally, the AI model incorporates organism-specific information through an organism tag, enabling the model to understand biological contexts more effectively. The model employs a curriculum learning strategy inspired by biological drug testing pipelines, progressively introducing data from simpler to more complex organisms during training (Figure 2A). This approach, termed biological complexity curriculum learning, ensures that the model captures relevant patterns and gains a real-world understanding of molecular structures and interactions (for further details regarding model development, see Methods and Experimental Procedures).

**Figure 2:**
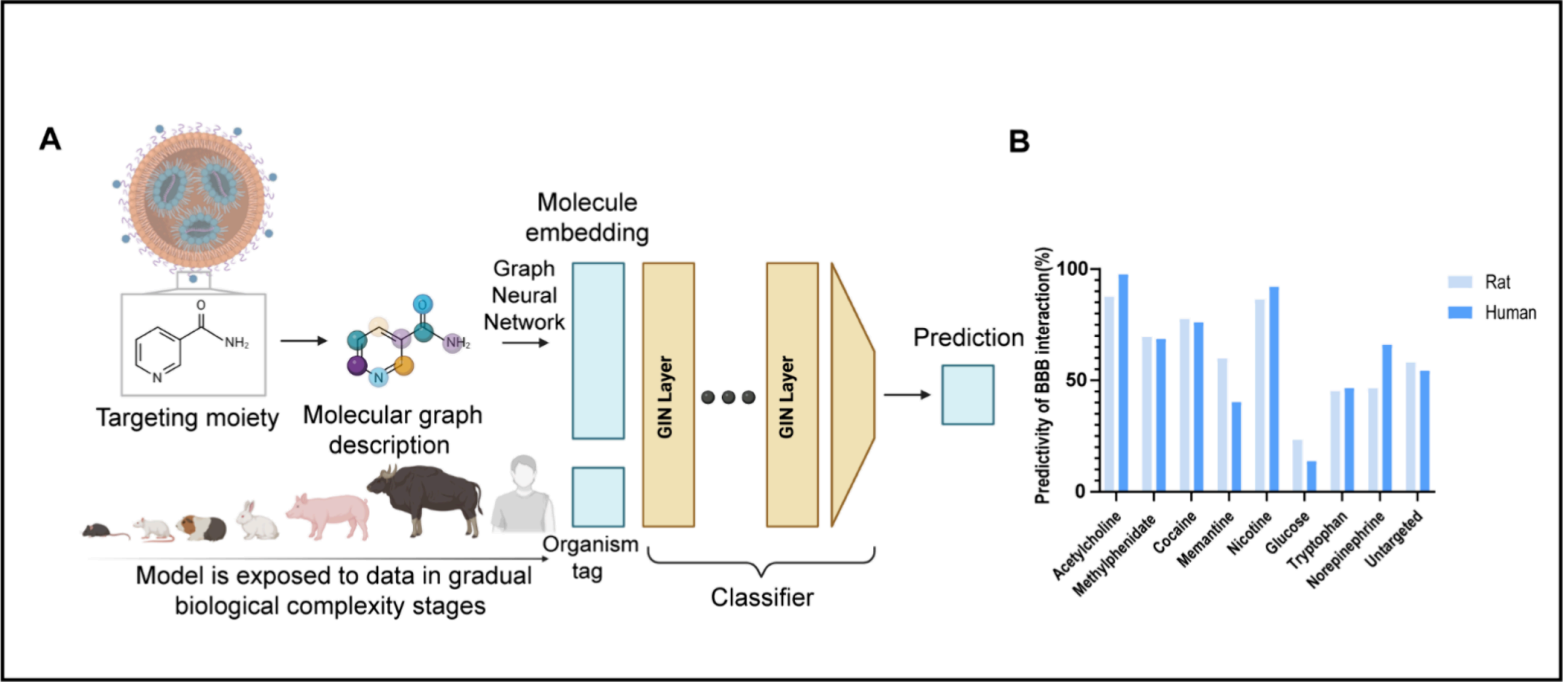
**Predicting molecule–BBB interaction potential across species using a graph neural network-based AI model.** (A) The AI model predicts the activity of a given molecule within a specific tissue of an organism. It begins by converting the molecule into a graph representation, which is encoded through a graph neural network (GNN) to generate a molecular embedding. In parallel, a curriculum learning strategy exposes the model to data from different organisms sequentially, and the organism information is encoded as an organism-specific tag. These embeddings are concatenated and processed by a classifier that predicts the molecule’s activity in the designated tissue. The model is trained for each tissue using all available organismal data, though not every tissue has data from all organisms, as shown in the figure. Illustration created in BioRender [Shklover, J. (2025) https://BioRender.com/fmeu0vq]. (B) The prediction of the molecule–BBB interaction potential (%) for the small-molecule library used in BT-LNPs was estimated using the developed AI model for both rat and human brains. Acetylcholine demonstrated the highest predicted probability of BBB interaction among all tested ligands. A methyl molecule was used to represent the untargeted condition.

We applied the AI-based model to predict the passage of selected BT-molecules across the BBB (Figure 2B). “Untargeted” is represented in calculations as a methyl group. In this case, the model was initially trained on a dataset [https://ui.staging.kit.cloud.douglasconnect.com/datasets] derived from *in vitro* studies, taking into account the chemical structure of each molecule. It was then fine-tuned on the BBBP dataset^52^ to help the model understand the specific factors influencing BBB permeability and predict its potential for molecular interaction with the BBB. Based on the prediction referred for both human and rat brains (no sufficient dataset for mouse brain prediction is yet available), acetylcholine and nicotine were the most promising candidates to facilitate BBB crossing (with 0.876, 0.976 and 0.894, 0.92 predictivity of BBB crossing in rats and humans, respectively) and, thus, will potentially improve brain uptake of LNPs. In contrast, tryptophan and glucose were the least promising candidates predicted by the model (with 0.452, 0.466, and 0.233, 0.138 predictivity of BBB crossing in rats and humans, respectively). These results will be further compared to our animal experimental data to validate the model *in vivo*. The code and datasets are publicly available [10.5281/zenodo.13863512].

### *In Vitro* Screening of BT-LNP Library

Next, we evaluated the transfection efficiency and cytotoxicity of the BT-LNP library (Figure 3A­(i),(ii), respectively). To do this, LNPs were loaded with firefly luciferase (FLuc) mRNA, and the expression was assessed in two cell lines: hCMEC/D3, a key component of the BBB basement membrane,^53^ and differentiated SH-SY5Y cells, which exhibit mature neuron-like phenotypes.^54^ Both cell lines were treated with the different LNP formulations for 16 h or left untreated as control groups (200 ng FLuc mRNA per treatment).

**Figure 3:**
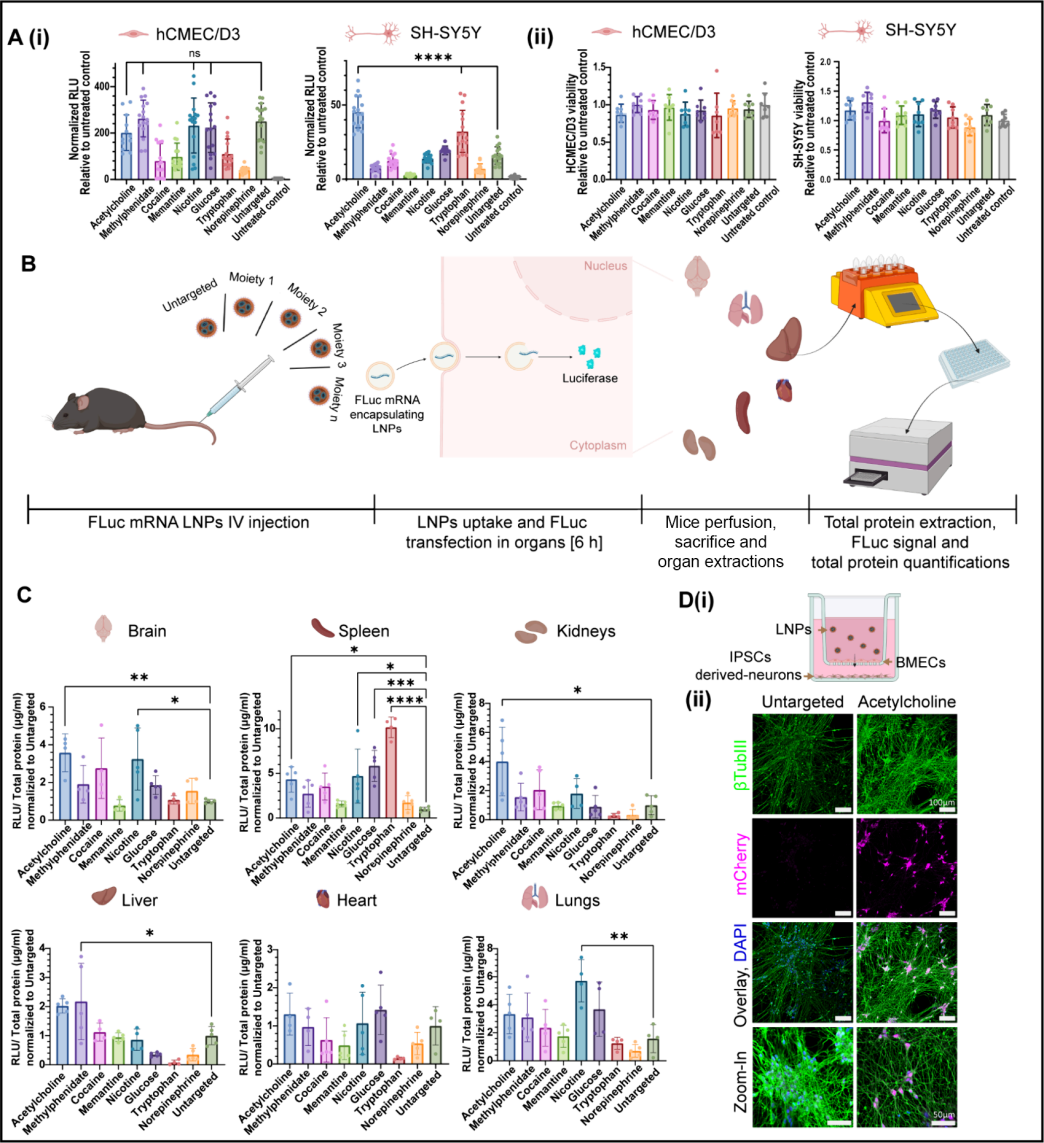
***In vitro* and *in vivo* screening and evaluation of brain targeted (BT)-LNPs.** (A) Cultures of hCMEC/D3 and differentiated SH-SY5Y cells were treated with firefly luciferase (FLuc)-encoding BT-LNPs to assess (i) the transfection efficiency (RLU = relative luminescence units) and (ii) the cytotoxicity. All treatments were normalized to the untreated control (*n* ≥ 12). ****p < 0.0001. (B) A high-throughput luminescence-based assay was used to evaluate the biodistribution profile and *in vivo* transfection efficiency of the FLuc-encoding BT-LNPs library following intravenous (IV) administration. (C) Transfection efficiency of BT-LNPs in various organs. Data in each graph are normalized to the untargeted control group within the same organ (*n* = 4–5 mice per treatment). *p ≤ 0.0357; **p = 0.0094; ***p = 0.0005; ****p < 0.0001. Results are presented as mean ± SD. One-way ANOVA with correction for multiple comparisons was used for statistical analysis. (D) (i) Schematic of the transwell-based BBB model. Human iPSC-derived brain microvascular endothelial cells (BMECs) were seeded on the upper side of the transwell insert, while human iPSC-derived neurons were cultured on the bottom (basolateral) side. Acetylcholine- or untargeted LNPs encoding mCherry mRNA were applied to the apical (BMEC-facing) compartment. (ii) Representative confocal images of the neuronal layer following 24 h of incubation. βTubIII (green) marks neuronal processes, mCherry (magenta) indicates successful transfection, and DAPI (blue) stains nuclei. Neurons exposed to acetylcholine-LNPs showed increased mCherry expression. *n* = 3 biological repetitions; scale bars = 100 μm and 50 μm for zoom-in images. Illustrations created in BioRender [Shklover, J. (2025) https://BioRender.com/fmeu0vq].

Generally, the luminescence values obtained in hCMEC/D3 after transfection were higher than those in SH-SY5Y cells, indicating that transfection in endothelial cells is more efficient than in neuronal cells. However, in hCMEC/D3 cells, no significant difference was observed in transfection efficiency between targeted and untargeted LNP formulations, suggesting that these targeting molecules may not enhance transfection in hCMEC/D3 cells.

In contrast, in differentiated SH-SY5Y cells, acetylcholine-LNPs (*p* < 0.0001), and tryptophan-LNPs (*p* < 0.0001) exhibited significantly greater transfection efficiency compared to untargeted LNPs (Figure 3A­(i)). Differentiated SH-SY5Y cells are known to express choline receptors,^55^ which might facilitate receptor-mediated endocytosis^56^ upon interaction with acetylcholine-LNPs. Similarly, tryptophan-LNPs showed enhanced uptake, likely due to the presence of large neutral amino acid transporters on the surface of SH-SY5Y cells, which are responsible for transporting essential amino acids such as tryptophan.^57^ These findings suggest that using acetylcholine and tryptophan as targeting moieties improves the specificity and efficacy of mRNA delivery to neuron-like cells *in vitro*.

To assess the potential toxicity of the LNP formulations, both cell lines were treated with the respective LNPs, and cell viability was evaluated using the PrestoBlue assay. None of the formulations caused significant cytotoxicity *in vitro*, using either the ethanol-based (both glucose-targeted and untargeted) formulations or the DMSO-based targeted formulations, suggesting that the DMSO concentration was below the cellular toxicity threshold (Figure 3A­(ii)).

### *In Vivo* Screening and Validation of the BT-LNP Library

To evaluate the impact of the targeted LNP on biodistribution and tissue-transfection efficiency, healthy male C57BL/6J mice were intravenously injected with FLuc-LNPs at a dose of 0.729 mg/kg mRNA. After 6 h, the mice were sacrificed, perfused with PBS, and their organs were collected and frozen on dry ice. The tissues were then homogenized using a gentleMACS Dissociator for total protein extraction. To quantify the bioluminescence signal, a 5′-fluoroluciferin substrate was added to the extracted protein solutions, and samples were measured using a microplate reader (Figures 3B and S3). The bioluminescent signal obtained for each organ was normalized to the total protein amount of the corresponding tissue.

Acetylcholine-LNPs (*p* = 0.0094) and nicotine-LNPs (*p* = 0.0205) demonstrated the highest signal in the brain compared to all other formulations, with acetylcholine-LNPs exhibiting approximately 3.6-fold more signal than the untargeted-LNPs (Figure 3C). Significant luciferase expression was also observed for acetylcholine-LNPs in the spleen (*p* = 0.0357) and kidneys (*p* = 0.0039), and for nicotine-LNPs in the spleen (*p* = 0.0134) and lungs (*p* = 0.0154), relative to the untargeted LNPs reference. These findings suggest off-target effects that necessitate further optimization of the lipid composition to enhance specificity.^58,59^

Overall, the acetylcholine moiety not only exhibited excellent *in vitro* transfection ability in neuron-like cells (Figure 3A­(i)) but also demonstrated the highest *in vivo* brain transfection compared to the control group. Beyond brain delivery, different organ-specific accumulations were observed in other LNP formulations. Compared to untargeted-LNPs, glucose-LNPs (*p* = 0.0005) showed significantly higher expression in the spleen. The spleen is rich in immune cells, particularly macrophages,^60^ which take up glucose due to their high metabolic activity,^61^ thereby facilitating the uptake of glucose-LNPs. A similar effect was observed for tryptophan-LNPs (*p* < 0.0001). Tryptophan, a hydrophobic, aromatic essential amino acid, is actively transported by immune cells and plays a critical role in immune metabolism, particularly in the spleen.^62^ The increased accumulation of tryptophan-LNPs in the spleen may result from preferential uptake by phagocytic immune cells such as macrophages and dendritic cells, potentially mediated through scavenger receptors or nonspecific hydrophobic interactions.^63^ Accumulating specific LNP formulations in off-target organs highlights key challenges in achieving selective targeting. These findings underscore the need for further targeting optimization strategies to minimize nonspecific uptake.

While some formulations were less effective in targeting the brain, others, particularly acetylcholine-LNPs, exhibited enhanced brain uptake, making them promising candidates for further investigation.

According to the *in vivo* experimental results and their comparison with predictions from AI model (Figure 2B), the model demonstrates high accuracy and strong correlation with real-world experimental data, with an area under the curve (AUC) value of 0.82. Notably, despite being trained on binary outcomes (pass/fail), the model effectively correlated high prediction scores with molecules that had higher experimental results and vice versa. This suggests that the AI model developed a nuanced understanding of molecular structures and interactions, indicating that fine-tuning with a task-specific dataset (the BBB passage) can significantly enhance its real-world performance.

However, the model struggled with predicting the BBB passage of glucose, a well-established targeting moiety known to interact with glucose transporters to facilitate brain uptake.^25,38^ This outcome could be due to a gap in molecular space coverage during training, where glucose was underrepresented in either the original training data or the BBB dataset. This lack of coverage might have limited the model’s ability to make accurate predictions for glucose and underscored the need to generate databases to improve AI-based targeted systems.

Similarly, results for rat data show that the model generally correlates well with real-world experiments, though there are some discrepancies. For instance, while the model correctly identified lower probabilities for brain targeting by norepinephrine, methyl, tryptophan, and memantine, it had difficulty distinguishing between them with high precision. This performance discrepancy highlights that while the AI model has a solid grasp of the general trend in rat BBB passage, fine distinctions between similar molecules can be challenging. In addition, the fact that the experimental data was received by examining targeted-LNPs, modified with the examined molecules, and studied by their ability for brain uptake in a different organism, e.g., mice, might introduce another reason for prediction inaccuracies.

Interestingly, the model’s predictions for human tissue were more accurate than those for rats. This difference can be attributed to the fact that the AI model was originally designed with a primary focus on predicting activity in human tissues, with the ability to adapt to other organisms, such as rats, as a secondary feature.

Overall, the AI model exhibits strong performance in predicting the BBB permeability of the BT-molecule library, with high accuracy and significant correlation with real-world data from a mouse model. This study provides the first validation of this AI model through practical *in vivo* results, confirming its reliability. Moreover, it underscores the potential of using AI models to streamline drug development and discovery by reducing both time and costs.

Next, to investigate the temporal dynamics of mRNA *in vivo* expression, mice were intravenously injected with glucose-LNPs, and a luciferase signal intensity was measured over time (Figure S4A). Over 6 days, a decline in signal intensity was observed in the liver and spleen, consistent with findings by Rizvi et al.^64,65^ In contrast, the signal reduction in the brain was more gradual and stable, indicating that the mRNA expression persisted longer in the brain than in the other organs (Figure S4B). The ability to sustain mRNA expression in the brain over an extended period highlights the potential of using LNPs for long-lasting neurological therapies.

### *In Vitro* Evaluation of BT-LNPs BBB Crossing

To better evaluate the ability of BT-LNPs to traverse the BBB, we employed an *in vitro* transwell-based model composed of induced pluripotent stem cell (iPSC)-derived human brain microvascular endothelial cells (BMECs).^36^ In this model, human BMECs are cultured in the upper compartment and iPSC-derived neurons in the basolateral chamber (Figure 3D­(i)).

To study the translocation of a targeted formulation across the BBB, we applied acetylcholine-LNPs loaded with mRNA encoding the fluorescent mCherry gene to the apical (BMEC-facing) compartment and incubated the system for 24 h. An untargeted LNP formulation was used for comparison. Confocal imaging for mCherry expression was tested in the BMEC monolayer under both conditions (Figure S5). Notably, the mCherry signal was detected in the neuronal layer of the basolateral compartment, with a stronger signal observed following application of acetylcholine-LNPs (Figure 3D­(ii)). These findings suggest that the acetylcholine modification promotes LNP transport across the endothelial barrier and improves delivery to postbarrier neuronal targets.

### BT-LNP Distribution in Brain Tissue Following Systemic Administration

To evaluate the ability of BT-LNPs to mediate cell-specific gene editing in the brain, we utilized genetically engineered tdTomato reporter mice (Ai9). These mice contain a LoxP-flanked stop cassette that prevents the expression of tdTomato protein.^67^ Upon the presence of Cre-recombinase, the stop cassette is removed via an enzymatic reaction, and the expression of tdTomato is allowed (Figure 4A).^68^ To leverage this model to study our system, we intravenously administered LNPs loaded with Cre recombinase mRNA (Cre mRNA). At this point, we proceeded exclusively with formulations demonstrating the highest potential for brain delivery and transfection *in vivo*: acetylcholine- and nicotine-LNPs. The untargeted LNP formulation served as a reference, and a control group of untreated Ai9 mice was used to establish baseline fluorescence. 48 h after systemic administration of Cre-encoding mRNA-LNPs, tdTomato expression in the brain was monitored (Figure S6).^15^ Brain tissues were harvested and enzymatically dissociated for single-cell analysis. Brain cells were labeled using an antibody panel to identify microglia (CD45+, CD11b+), endothelial cells (CD31+), astrocytes (CD44+, ACSA-2+), oligodendrocytes (O4+), and neurons (CD24+) (Figure S7A). The mean fluorescence intensity (MFI) of tdTomato was then quantified for each LNP formulation in each cell population using a gradual elimination gating strategy (Figure S7B­(i),(ii)).

**Figure 4:**
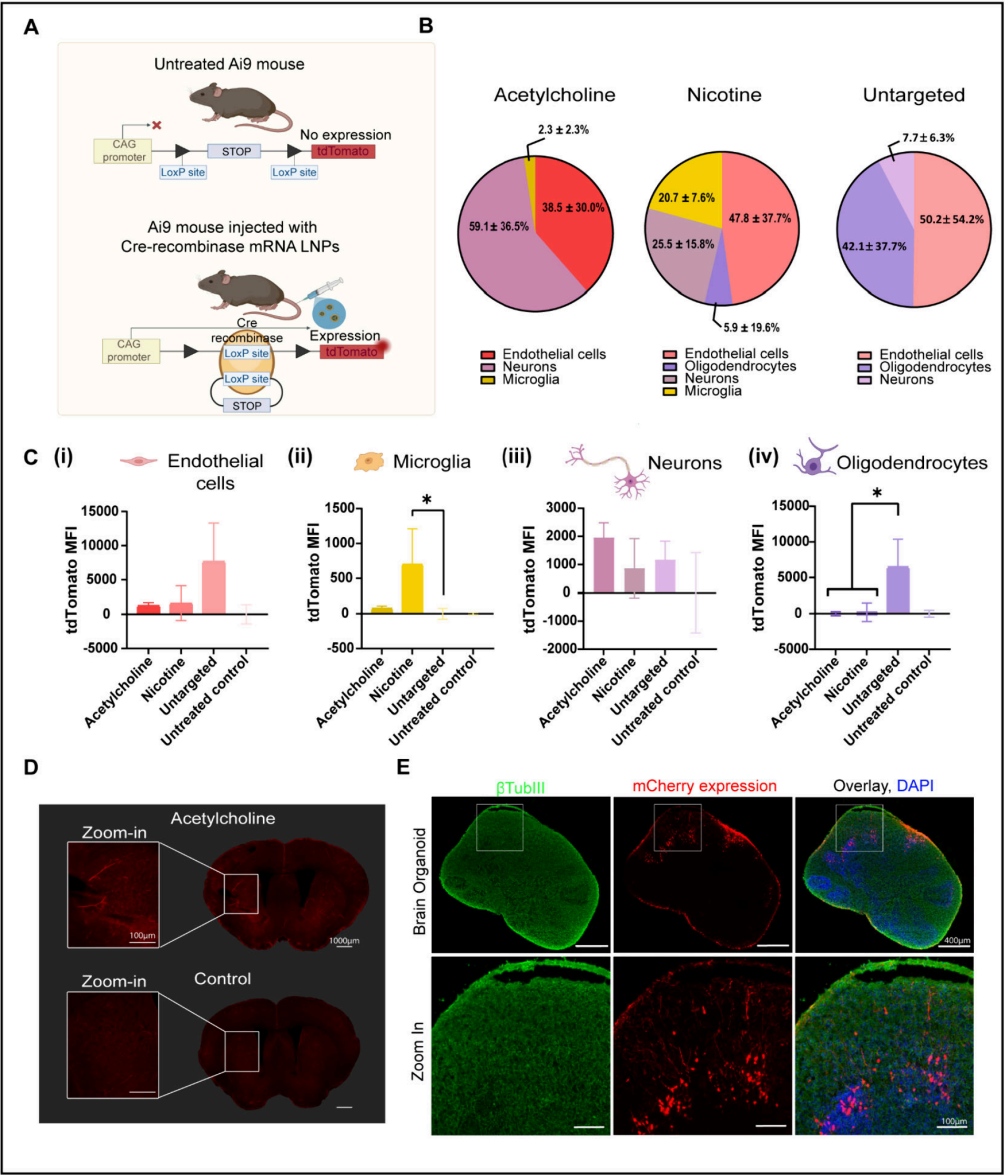
**Cellular-level examination and visualization of selected BT-LNPs in the mouse brain and human brain organoid.** (A) Ai9 Cre reporter mice, which express tdTomato protein under the control of the CAG promoter upon Cre-mediated recombination, were used to study brain-cell selectivity and uptake of selected BT-LNP formulations. (B) Distribution (%) of each LNP formulation across major brain cell populations, shown as pie charts, illustrating cell-type preferences in vivo. (C) Mean fluorescence intensity (MFI) of tdTomato signal in each brain cell population: endothelial cells (i), microglia (ii), neurons (iii), and oligodendrocytes (iv) following LNP treatment. Astrocytes showed no significant positive signal compared to the control group (*n* = 3 mice per treatment). *p ≤ 0.0403. Data are presented as mean ± SD. One-way ANOVA with multiple comparisons adjusted *p* values were used for statistical analysis. (D) 48 h after acetylcholine-LNPs administration, brain sections show a widespread tdTomato expression, predominantly localized along vascular structures. A noninjected mouse served as a control. Images are shown at 4× magnification; scale bar = 1000 μm; zoom-in scale bar = 100 μm. (E) Human iPSC-derived cortical brain organoids were treated with acetylcholine-LNPs encapsulating mCherry mRNA (*n* = 2 independent groups). After 48 h, confocal imaging demonstrated mCherry expression (red) across both peripheral and deeper organoid regions, including rosette-like structures. Neurons are marked by βTubulin III (βTubIII, green) and nuclei by DAPI (blue); scale bars = 400 μm (top), 100 μm (bottom). Illustrations created in BioRender [Shklover, J. (2025) https://BioRender.com/fmeu0vq].

First, to gain a clearer understanding of the cell-type distribution of each LNP formulation in the brain, we quantified the relative tdTomato MFI contributed by each cell population to the total tdTomato signal per treatment group using the following formula:

$$\mathrm{relative}\;\mathrm{MFI}=\frac{\mathrm{cell‐type}\;\mathrm{tdTomato}\;\mathrm{MFI}}{\mathrm{total}\;\mathrm{tdTomato}\;\mathrm{MFI}}\times 100$$

As shown in Figure 4B, neurons were the dominant contributors to the total tdTomato signal in the acetylcholine-LNP group (59.1 ± 36.5%). In contrast, their contribution in the nicotine-LNP and untargeted-LNP groups was substantially lower (25.5 ± 15.8% and 7.7 ± 6.3%, respectively). Endothelial cells were the predominant contributors to the total signal for both nicotine-LNP and untargeted-LNPs. Notably, oligodendrocytes contributed substantially to the untargeted-LNP signal (42.1 ± 37.7%), potentially reflecting their anatomical proximity to the vasculature and high metabolic demands for myelin production, both of which could facilitate greater nanoparticle uptake.^69–71^

Further analysis of absolute MFI values revealed that the untargeted-LNPs exhibited the highest signal within endothelial cells (7670 ± 5635), approximately ∼4.7-fold and ∼6.0-fold higher than that observed with nicotine-LNPs and acetylcholine-LNPs, respectively (Figure 4C­(i)). Additionally, this formulation elicited a significantly elevated tdTomato signal in oligodendrocytes (*p* = 0.0223) (Figure 4C­(iv)). These findings suggest that in the absence of a targeting ligand, LNPs preferentially accumulate in endothelial and perivascular cell types, indicating their natural tropism for the BBB and its surrounding microenvironment.

Interestingly, nicotine-LNPs produced significantly higher tdTomato expression in microglia compared to the untargeted-LNPs (*p* = 0.0403) (Figure 4C­(ii)). This may reflect the known interaction of nicotine and its metabolites, such as cotinine, with nicotinic acetylcholine receptors on microglial cells, potentially enhancing their uptake of nicotine-modified nanoparticles.^72,73^ In neurons, acetylcholine-LNPs induced the highest tdTomato expression (1963 ± 521), approximately twice that of untargeted- LNPs (Figure 4C­(iii)). No significant tdTomato signal was detected in astrocytes across treatment groups compared to untreated Ai9 controls.

Overall, these findings highlight the abilities of acetylcholine-LNPs and nicotine-LNPs to effectively deliver mRNA to neurons and microglia. In contrast, untargeted-LNPs primarily accumulated in endothelial cells and oligodendrocytes, demonstrating the adaptability of LNP design for precise therapeutic delivery to specific brain cell types.

To visualize tdTomato expression in the brain, we selected acetylcholine-LNPs that were identified in our screen as the most promising formulation for systemic brain and neuronal-targeted delivery. Brains were collected 48 h postinjection for subsequent analysis. Fluorescent microscopy of cryosectioned brain tissue showed broad tdTomato expression, primarily localized along vascular structures (Figures 4D and S8). In addition, *ex vivo* imaging performed following the same administration protocol demonstrated widespread tdTomato fluorescence in the brains of acetylcholine-LNPs-treated mice (Figure S6B­(i)). Together, these imaging modalities independently confirm brain delivery and Cre-mediated recombination following systemic administration of acetylcholine-LNPs.

### Transgene Expression in Deep Layers of Human Brain Organoids Using Selected BT-LNPs

To evaluate the relevance of the acetylcholine-LNPs in a human tissue model, we applied untargeted or acetylcholine-conjugated mCherry-encoding LNPs to human iPSC-derived cortical brain organoids, which mimic key features of early neurodevelopment and three-dimensional cytoarchitecture.

At 48 h after treatment, we observed mCherry fluorescent expression throughout the organoid, indicating that LNP formulations were capable of supporting transgene expression in this complex system. Notably, the fluorescence signal was evident not only in the peripheral regions but also within deeper zones of the organoid, primarily in rosette-like structures (Figure 4E and Figure S9). For acetylcholine-LNPs treatment, expression was observed in both neuronal soma and extended along neurite structures, suggesting successful transfection of morphologically mature neurons (see the zoom-in views in Figure 4E). These observations demonstrate that acetylcholine-LNPs effectively penetrate human iPSC-derived brain organoids and mediate gene expression within complex, multilayered neural tissue, supporting their potential as a delivery system in human settings.

### Mechanistic Evaluation of Acetylcholine-LNP Cellular Uptake

To explore the mechanism by which acetylcholine-LNPs enhance cellular uptake and mRNA delivery, we conducted a series of inhibition experiments using two CNS-relevant models: tight junction–forming BMECs and SH-SY5Y.

To assess the role of acetylcholine receptors (AchRs) in acetylcholine-LNP uptake, cells were pretreated with scopolamine (Scop), a muscarinic AchR antagonist; mecamylamine (Mec), a nicotinic AchR antagonist; or a combination of both. Following pretreatment, cells were exposed to luciferase-encoding mRNA-loaded acetylcholine- or untargeted control LNPs. Luminescence was measured after overnight incubation to assess transfection efficiency.

In both BMECs and SH-SY5Y cells, acetylcholine-LNPs induced higher luminescence than untargeted LNPs, consistent with improved the cellular uptake and gene expression. While pretreatment with either Scop or Mec alone resulted in modest, nonsignificant reductions in luminescence, dual receptor blockade significantly decreased transfection efficiency in the BMECs setup. Importantly, this reduction did not entirely abolish acetylcholine-LNP activity, suggesting that receptor engagement contributes meaningfully, but not exclusively, to the internalization process. Untargeted LNPs consistently yielded comparatively low luminescence signals across all conditions, including in the presence of receptor inhibitors, serving as a negative control and underscoring the specificity of acetylcholine-mediated effects (Figures 5A­(i),(ii) and S10A).

**Figure 5:**
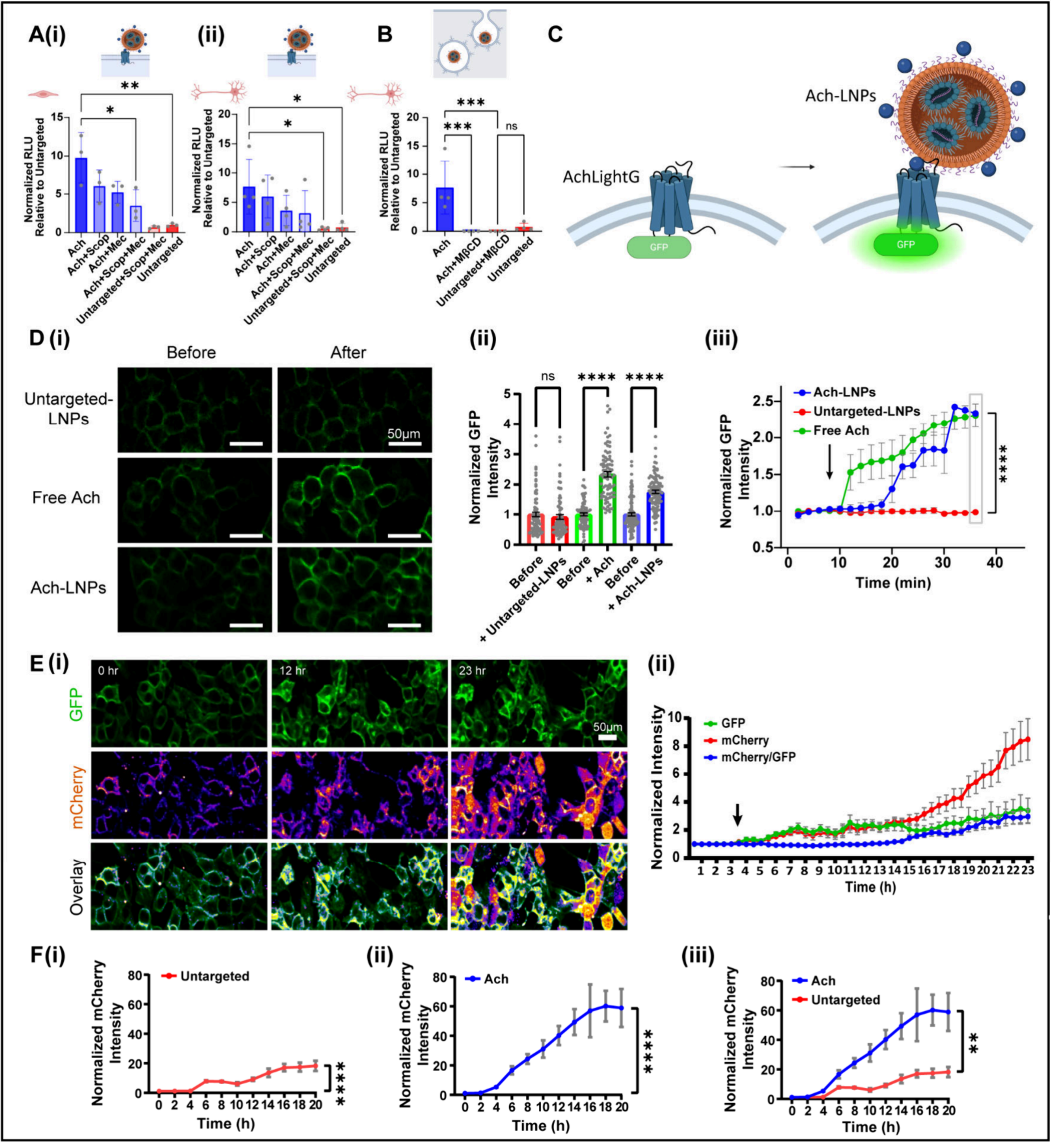
**Mechanistic investigation of acetylcholine-conjugated LNPs (Ach-LNPs) cellular uptake.** (A) (i), (ii) Luciferase mRNA-loaded Ach-LNPs were applied to BMECs (in a transwell BBB model) and SH-SY5Y neuronal-like cells. Cells were treated with Ach-LNPs alone or preincubated with acetylcholine receptor inhibitors: scopolamine (Scop, muscarinic antagonist) or mecamylamine (Mec, nicotinic antagonist). Untargeted LNPs served as controls. (B) To assess endocytosis involvement, SH-SY5Y cells were pretreated with methyl-β-cyclodextrin (MβCD), a caveolae-mediated endocytosis inhibitor. Data from A and B are presented as normalized luminescence units (RLU) relative to untargeted control (mean ± SD, *n* ≥ 3). *p = 0.0216; **p = 0.0011; ***p = 0.0010. (C) AchLightG sensor activation occurs upon Ach-LNP binding, inducing GFP fluorescence for real-time tracking. (D) (i) Representative images of HEK293 cells expressing AchLightG before and after 35 min of treatment with untargeted LNPs, free Ach (100 μM), or Ach-LNPs. Increased green fluorescence indicates sensor activation. Scale bar = 50 μm. (ii) Quantification of normalized GFP intensity before/after 35 min; both free Ach and Ach-LNPs significantly increased GFP signal (*n* ≥ 80). (iii) Time course of normalized GFP fluorescence shows significant increases (****p < 0.0001) after Ach or Ach-LNP treatment. The arrow shows the treatment application time. (E) (i) Time-lapse imaging of AchLightG-expressing HEK293 cells treated with mCherry mRNA-loaded Ach-LNPs shows progressive mCherry and GFP signal over 23 h. Scale bar = 50 μm. AchLightG sensor activation is shown in green (GFP) and mCherry expression on the fire scale. Scale bar = 50 μm. (ii) Quantification of GFP, mCherry, and mCherry/GFP intensity over time. (F) (i)–(iii) Quantification of normalized mCherry fluorescence up to 20 h post-LNP treatment (*n* = 60). Ach-LNPs yielded significantly higher final mCherry signal (**p = 0.0028). *n* = number of cells; error bars denote SEM; statistics by Student’s *t* test or one-way ANOVA with *post hoc* Tukey’s test. Experiments were independently repeated at least 3 times. Illustrations created in BioRender [Shklover, J. (2025) https://BioRender.com/fmeu0vq].

These results imply that both muscarinic and nicotinic receptors may be involved in a cooperative uptake mechanism, and that their engagement facilitates, but does not solely mediate, the internalization of acetylcholine-LNPs. The incomplete inhibition further indicates that nonreceptor-mediated interactions or alternative pathways may participate in the uptake process.

To further investigate the mechanistic basis of acetylcholine-LNP uptake, we examined the role of membrane cholesterol in SH-SY5Y cells using methyl-β-cyclodextrin (MβCD), a well-established disruptor of lipid rafts. This approach was motivated by prior work showing that particular LNP transport mechanisms across the BBB can be sensitive to raft disruption^22^ and by the hypothesis that acetylcholine-LNPs may rely on cholesterol-rich domains for efficient internalization.

Pretreatment with MβCD led to a near-complete loss of acetylcholine-LNP transfection (Figures 5B and S10B). Notably, MβCD did not significantly affect the already low transfection by untargeted LNPs, suggesting that the observed effect is specific to the acetylcholine-conjugated formulation. These findings suggest that membrane cholesterol plays a role in acetylcholine-LNP internalization, supporting the involvement of a raft-associated uptake mechanism, possibly through lipid raft-facilitated receptor clustering or caveolar endocytosis. While the precise endocytic pathway remains to be defined, the data suggest that cholesterol-rich membrane microdomains act as a permissive platform for acetylcholine-LNP internalization.

To assess the functional interaction between our acetylcholine-LNPs and cell surface AchRs, we employed the genetically encoded fluorescent reporter system AchLightG (also known as nLightG), expressed in HEK293 cells. AchLightG is based on an engineered M3-muscarinic G protein-coupled receptor (GPCR) scaffold coupled to a circularly permuted green fluorescent protein (cpGFP) inserted within the intracellular loop (ICL3), which produces robust green fluorescence upon AchR agonist binding (Figure 5C). This sensor enables sensitive and real-time monitoring of ligand-induced receptor activation, with a high signal-to-noise ratio and temporal resolution.^74^

Upon application of acetylcholine-LNPs to AchLightG-expressing HEK293 cells, we observed a rapid increase in green fluorescence, indicating that the surface-conjugated acetylcholine moieties retain their functional ability to engage with GPCR-type muscarinic AchRs and trigger a conformational response. Free acetylcholine induced a similarly robust activation, whereas untargeted LNPs showed no appreciable signal (Figure 5D­(i),(ii) and Movies S1 and S2). A kinetic analysis further demonstrated that the acetylcholine-LNP-induced fluorescence increase followed a time-dependent profile, plateauing after ∼35 min, closely resembling the response to free acetylcholine (Figure 5D­(iii)). Interestingly, acetylcholine-LNP-treated cells exhibited a noticeable delayed fluorescence onset relative to free acetylcholine, suggesting slower engagement with cell-surface receptors. This lag in response may be attributed to steric hindrance imposed by the PEG corona on the LNP surface, which can reduce ligand accessibility and restrict immediate receptor binding. In contrast, free acetylcholine molecules, being small and unconjugated, likely diffuse and interact more rapidly with the AchRs. Despite this initial delay, acetylcholine-LNPs ultimately achieved a robust activation of the AchLightG sensor, supporting the conclusion that the conjugated ligand remains functionally capable of eliciting receptor-mediated responses over time.

Next, to determine whether this receptor engagement facilitates functional mRNA delivery, we loaded acetylcholine-LNPs with mCherry-encoding mRNA and monitored protein expression over time in AchLightG-expressing cells. Cells treated with acetylcholine-LNPs exhibited time-dependent accumulation of mCherry fluorescence, with signal colocalizing in GFP-positive cells. The mCherry/GFP ratio isolates an increase in mCherry expression over time by minimizing the green-to-red spectral bleed-through during dual-channel fluorescence acquisition (Figures 5E­(i),(ii) and S11 and Movie S3). Quantification revealed significantly higher expression levels in the acetylcholine-LNP-treated group compared to the untargeted-LNP control (Figure 5F­(i)–(iii)), confirming enhanced delivery and translation efficiency via receptor-mediated uptake.

In summary, the enhanced transfection efficiency observed with acetylcholine-LNPs compared to untargeted LNPs likely stems from their ability to engage in specific interactions with AchRs on the cell surface, even in the absence of classical receptor-mediated internalization. Although the AchLightG sensor used in this study does not internalize upon ligand binding, the anchoring of acetylcholine-LNPs to surface AchRs likely increases their residence time and local concentration at the plasma membrane. This prolonged interaction may increase the probability of cellular uptake, most likely through endogenous endocytic pathways. Such receptor-assisted proximity may be sufficient to initiate internalization, especially in contexts where spontaneous nanoparticle–membrane interactions are otherwise limited.

These data support a model in which receptor recognition and membrane biophysics synergize to enhance nanoparticle uptake and functional mRNA delivery. This provides a strong rationale for ligand-guided design of brain-targeted nanocarriers.

### Cellular Specificity of BT-LNPs Following Intracerebral Injection

Our flow cytometry analysis demonstrated the potential brain cell specificity of the selected BT-LNPs. However, the complexity of BBB crossing and the basal tdTomato expression in the Ai9 model resulted in a limited detected signal within certain cell types and a relatively narrow population of tdTomato-positive cells. Therefore, further investigation was required to elucidate LNPs’ cellular selectivity. For this reason, we performed intracerebral injections of acetylcholine-LNPs and untargeted-LNPs, containing Cre mRNA, into the cortex of the brain of Ai9 mice. LNPs loaded with FLuc mRNA served as a negative control (Figure 6A).

**Figure 6:**
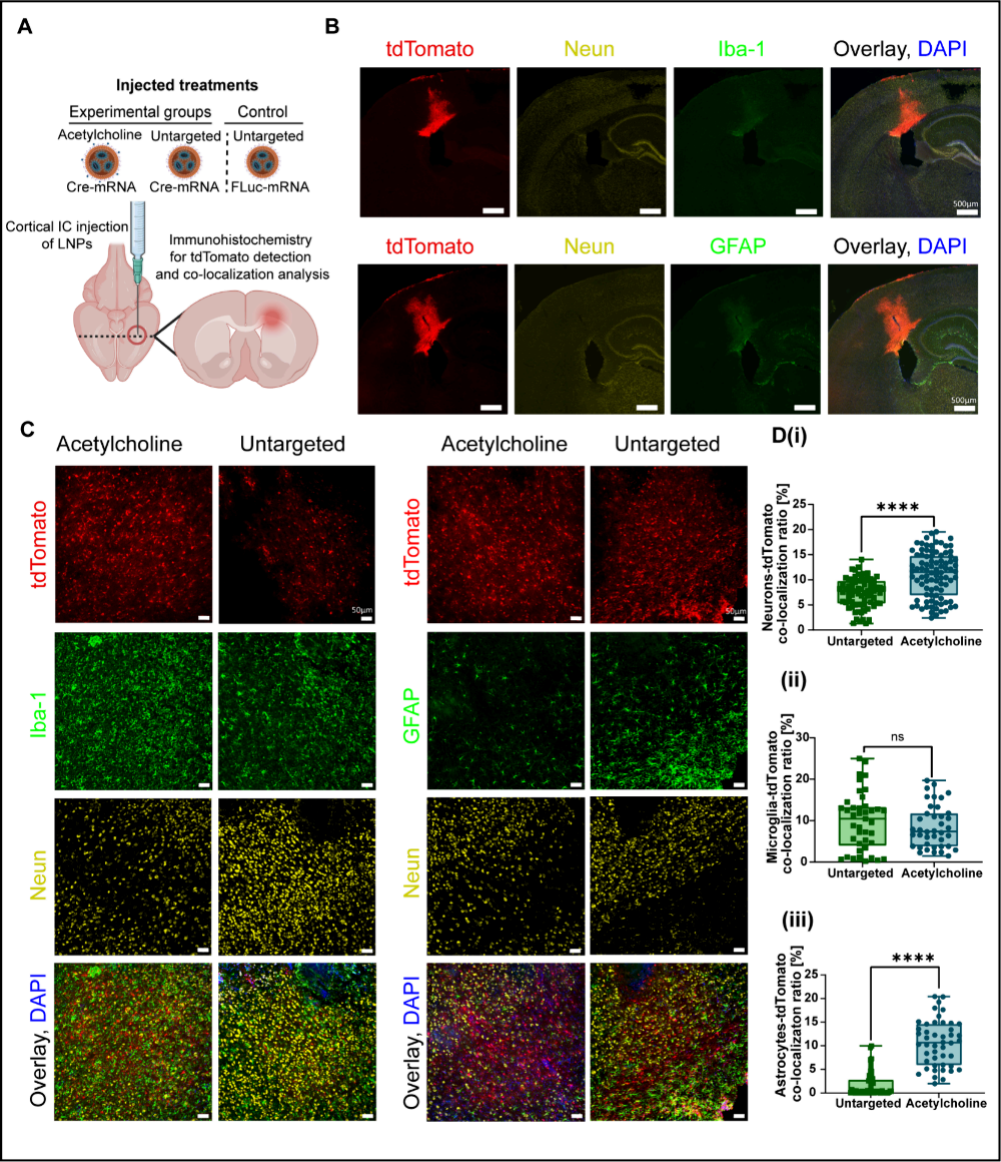
**Cellular specificity of BT-LNPs following intracerebral (IC) injection.** (A) IC injections of Cre-encoding acetylcholine and untargeted LNPs were conducted to compare their cellular specificity within the brain. Untargeted FLuc-mRNA LNPs were included as a negative control to set the basal tdTomato expression. Illustration created in BioRender [Shklover, J. (2025) https://BioRender.com/fmeu0vq]. (B) Representative images of brain sections that were fixed and immunostained for NeuN (neurons), Iba1 (microglia, (i)), and GFAP (astrocytes, (ii)). Cell nuclei were counterstained with DAPI. LNP uptake and endosomal escape were confirmed by the expression of tdTomato. Images were captured at 10× magnification; scale bar = 500 μm. (C) Representative higher-magnification images (20×) of cell staining in slides of both acetylcholine- and untargeted LNPs injected brains. Scale bar = 50 μm. (D) Image analysis of tdTomato colocalization ratio (%) in brain cells, including neurons (i), microglia (ii), and astrocytes (iii). Data are expressed as mean ± SD (*n* = 4 independent experiments, each with at least 40 replicates per group); unpaired *t* test *p* value; ****p < 0.0001.

Histological brain sections were stained for neuronal nuclear protein (NeuN), a neuronal marker;^75^ glial fibrillary acidic protein (GFAP), an astrocytic marker;^76^ and ionized calcium-binding adaptor molecule 1 (Iba1), a microglial marker^77^ (Figures 6B,C and S12, and S13).

For the quantification analysis, each image underwent a series of consecutive processing steps (see Methods and Experimental Procedures and Figure S14). The acetylcholine-LNPs group exhibited a significantly higher percentage of overlay colocalization (tdTomato+; NeuN+) compared to the untargeted-LNPs group (*p* < 0.0001) (Figure 6D­(i)). This finding aligns with earlier flow cytometry results (Figure 4B and Figure 4C­(iii)), which also demonstrate enhanced neuronal targeting and expression in comparison to the untargeted-LNPs. We further evaluated the percentage of overlay colocalization (tdTomato+; Iba1+), where neither the acetylcholine-LNPs nor the untargeted-LNPs showed specificity for microglial targeting (Figure 6D­(ii)), consistent with the earlier flow cytometry data.

Additionally, analyzing the overlay colocalization of tdTomato-positive cells with brain cell types other than microglia and neurons (Figure S15A) revealed that the untargeted-LNPs group exhibited significantly higher expression in these non-neuronal and nonmicroglial populations compared to the acetylcholine-LNPs group (*p* = 0.0002). These results highlight the low targeting specificity of untargeted-LNPs, as they do not selectively interact with specific brain cell populations. Untargeted-LNPs also demonstrated significantly higher overlay colocalization of tdTomato-positive cells with brain cell types other than astrocytes and neurons (*p* < 0.0001, Figure S15B), further underscoring their nonselectivity.

Interestingly, analysis of overlay colocalization (tdTomato+; GFAP+) showed significantly higher values for the acetylcholine-LNPs group compared to the untargeted-LNPs group (*p* < 0.0001) (Figure 6D­(iii)). Similar to neurons, astrocytes express acetylcholine receptors, specifically muscarinic acetylcholine receptors, which are more densely populated on astrocytes than on microglia.^78–80^ This likely explains the acetylcholine-LNPs’ ability to target astrocytes. Notably, while flow cytometry detected no expression in astrocytes following intravenous administration, intracerebral administration led to detectable expression in these cells. After systemic administration, a significant portion of LNPs accumulates in BBB endothelial cells and peripheral organs. However, in contrast, with intracerebral administration, localized LNPs are localized in a specific brain area, where they are primarily preferentially taken up by neurons and supportive brain cells. This targeted localization reduces the likelihood of missing certain cell populations, enabling more accurate quantification of LNPs transfection of astrocytes.

Overall, these results indicate that acetylcholine LNPs show promising potential for selectively targeting neurons and astrocytes, making them a strong candidate for therapeutic applications in genetic disorders affecting these cell types and neurodegenerative diseases. In contrast, untargeted LNPs exhibit a broader, less specific distribution, which may limit their effectiveness in targeted therapies.

## Conclusions

Recent efforts in LNP engineering have focused on expanding delivery beyond the liver, including strategies to modulate organ selectivity and enabling access to extrahepatic targets such as the brain. Recently, several targeting strategies have been reported, such as modulating LNP biodistribution through the incorporation of charge-altering lipids, enabling improved delivery to organs such as the spleen and lungs.^15^ Complementary strategies include the use of targeting ligands or peptides that promote receptor-mediated transcytosis.^21,22^

In this study, we developed small-molecule PEG-lipid conjugates that engage BBB transporters and receptors and incorporated them into the LNP formulation with enhanced receptor accessibility. This strategy establishes a stable and scalable platform for brain delivery with precise cell-type targeting. These features position our platform as a robust and versatile addition to the current landscape of CNS gene delivery.

*In vitro* evaluations showed that the BT-LNPs exhibited enhanced transfection efficiency in neuron-like cells (SH-SY5Y) without further cytotoxicity. Among the tested formulations, acetylcholine-LNPs demonstrated the highest transfection efficiency, supporting their potential for delivering therapeutic mRNA to neurons affected by neurodegenerative diseases.^81^ Their performance was further validated by enhanced mRNA delivery to human iPSC-derived neurons following translocation across a tight BMEC monolayer and effective transfection in the deeper layers of human cortical brain organoids.

Our *in vivo* studies confirmed the efficacy of these BT-LNPs, with acetylcholine-LNPs achieving a 3.6-fold increase in brain uptake and nicotine-LNPs also exhibiting superior brain uptake compared to untargeted-LNPs.

The integration of AI-driven models has the potential to significantly enhance the prediction of LNP biodistribution and efficacy, offering to reduce the synthetic burden of large chemical libraries, as well as the reliance on animal studies by accurately predicting *in vivo* outcomes.^24,82^ In this study, *in vivo* data were validated by an AI-based model, designed to predict molecular activity and drug efficiency across various tissues, particularly in humans. By incorporating both molecular structure and biological context, we observed strong concordance between the experimental and computational data, with acetylcholine and nicotine molecules identified as the most promising candidates for BBB crossing in human and rat models. This validation highlights the potential of using computational models as a powerful tool for optimizing LNP design, refining the screening process, and enhancing targeting specificity.

While our AI-driven approach has demonstrated strong predictive performance in identifying effective BBB-interacting molecules for LNP-based drug delivery, it is important to acknowledge potential biases, such as those affecting glucose-level-related predictions, and consider their broader implications. One key limitation arises from the reliance on available datasets to train our predictive model. Many public and proprietary datasets used for BBB permeability prediction have inherent biases due to the underrepresentation of specific chemical scaffolds, metabolic pathways, or species-specific variations. In particular, interactions, such as glucose-BBB, may be affected by variability in glucose transporter expression across different biological conditions, such as disease states (e.g., diabetes or neurodegeneration).^83^

Additionally, the dataset contains significantly more inactive (meaning, no predicted BBB interaction) than active samples in particular species (e.g., rats with 2770 inactive and 706 active samples). Such imbalances may skew the model’s learning process, leading to an overrepresentation of negative predictions, particularly in underrepresented species.

Despite these limitations, our findings demonstrate that AI-enhanced molecular screening provides valuable insights into BBB permeability. Continually refining these models will contribute to more accurate and personalized therapeutic strategies.

The selectivity of LNPs for specific brain cell types represents a new level of precision targeting, with the potential to more accurately treat diseases affecting distinct cellular populations. Flow cytometry and imaging results provided valuable insights into the cellular specificity of the lead LNP formulations within the brain. Following intracerebral injection, acetylcholine-LNPs exhibited a strong preference for transfecting neurons and astrocytes. This is significant for treating neurological disorders like Alzheimer’s and Parkinson’s disease that primarily affect neurons^84,85^ as well as conditions involving astrocyte dysfunction, such as multiple sclerosis^86^ and Alexander disease.^87^ Nicotine-LNPs were particularly effective in targeting microglia, the brain’s resident immune cells; a promising finding for conditions involving neuroinflammation, such as multiple sclerosis^88^ or traumatic brain injury.^89^ In contrast, the broader distribution of untargeted-LNPs, which predominantly accumulated endothelial cells and oligodendrocytes, could be beneficial for addressing diseases associated with the BBB microenvironment.^90^

Our mechanistic studies of the lead formulation, acetylcholine-LNPs, suggest that internalization occurs through a cooperative, receptor-mediated process likely involving endocytic uptake through cholesterol-rich membrane domains. Using a genetically encoded fluorescent reporter system, we further demonstrated that acetylcholine-LNPs engage cell surface acetylcholine receptors to promote significantly enhanced mRNA delivery and transgene expression compared to untargeted LNPs.

In conclusion, our research introduces a robust and versatile approach to developing brain-targeted mRNA-carrying LNPs with high cellular specificity and efficient delivery of genetic payloads. The ability of these LNPs to selectively target and transfect specific brain cell populations, such as neurons, astrocytes, and microglia, represents a significant importance in precision medicine. We show that integrating AI-based predictive modeling with experimental validation enhances the screening design of brain-targeted nanoparticles. Mechanistic data on acetylcholine-LNPs provide insights for the cellular interactions and internalization process of small-ligand conjugated LNPs, advancing our understanding of how small-molecule surface modifications influence nanoparticle interactions with cellular barriers and target cell populations.

## Materials and Experimental Procedures

### Synthesis of the Brain-Targeted Lipid Library

For all synthesized materials, NMR spectroscopy was conducted by TAMI-IMI ICL Central Institute for Research & Development (see Compound Spectra S1–S9).

#### DMG-PEG2000-tryptophan

Boc-tryptophan (8530380025; Sigma-Aldrich; 24.3 mg, 0.0795 mmol) was dissolved in 1.5 mL of DMF, and then *N*-hydroxysuccinimide (NHS) (130672; Sigma-Aldrich; 13.7 mg, 0.112 mmol) and *N*-ethyl-*N*′-(3-(dimethylamino)­propyl)­carbodiimide (EDC) (341006; Sigma-Aldrich; 22.9 mg, 0.112 mmol) were added. The reaction was stirred overnight at 25 °C under an inert atmosphere. The activated product Boc-tryptophan-NHS (0.0795 mmol) was added to 0.55 mL of chloroform containing DMG-PEG2000-NH_2_ (DMG-PEG2k-AM; Nanocs; 50 mg, 0.0199 mmol) and triethylamine (3 μL). The reaction was stirred overnight at 25 °C. The product was precipitated by adding 100 mL of heptane and purified by rotary evaporation (Buchi; Switzerland). Boc protecting group was removed by stirring in 1 mL of TFA and 50 μL of H_2_O for 1 h at 25 °C. The product was dried and dissolved in 2 mL of DMF and 4 mL of H_2_O, then dialyzed against water using a 2000 Da dialysis membrane (132625; Repligen, USA) at 4 °C with three buffer exchanges to remove residual reagents. DMG-PEG2000-tryptophan was obtained after lyophilization and stored as 10 mM dry DMSO stock at −80 °C until use. The product was identified by TLC and characterized by ^1^H NMR.

#### DMG-PEG2000-norepinephrine

Droxidopa (D9628; Sigma-Aldrich; 48 mg, 0.219 mmol) was dissolved in 2.25 mL of 10% NaHCO_3_/THF (2:1 v/v) and cooled to 0 °C. Fmoc-Cl (23184; Sigma-Aldrich; 0.2415 mmol) in 0.78 mL of THF was added dropwise. The reaction was stirred overnight at 25 °C and then evaporated under reduced pressure. The product was extracted with ethyl acetate and the aqueous layer was acidified to pH 2 with 6 M HCl. Extraction with ethyl acetate was repeated and the organic layer was evaporated under reduced pressure after dilution in isopropanol. The crude Fmoc-droxidopa was redissolved in 10 mL of ethyl acetate, and then 2.9 mL (0.0796 mmol) was activated by reaction with EDC (22.9 mg, 0.112 mmol) and NHS (13.7 mg, 0.112 mmol) in 1 mL of DMF overnight at 25 °C. After evaporation, the activated product Fmoc-droxidopa-NHS was redissolved in 0.5 mL of DMF and added to 0.5 mL of chloroform containing DMG-PEG2000-NH_2_ (50 mg, 0.0199 mmol) and triethylamine (3 μL). The reaction was stirred overnight at 25 °C. The product was precipitated by adding 100 mL of heptane and purified by rotary evaporation and dialysis as described above. The Fmoc protecting group was removed by stirring in 20% piperidine/DMF for 2 h at 25 °C. Finally, the product was precipitated with heptane, purified by rotary evaporation, dissolved in 2 mL of DMF and 4 mL of H_2_O, and dialyzed against water (MWCO 2000 Da) at 4 °C with three buffer exchanges to remove residual reagents. DMG-PEG2000-droxidopa was obtained after lyophilization and stored as 10 mM dry DMSO stock at −80 °C until use. The product was identified by TLC and characterized by ^1^H NMR.

#### DMG-PEG2000-glucose

d-Glucuronic acid (G5269; Sigma-Aldrich; 22.0 mg, 0.113 mmol) was dissolved in 1 mL of DMF, and then EDC (23.9 mg, 0.125 mmol) and NHS (14.4 mg, 0.125 mmol) were added. The reaction was stirred overnight at 4 °C under an inert atmosphere. The activated product d-glucuronic-NHS (0.0756 mmol) was added to 0.45 mL of chloroform and 0.5 mL of DMF containing DMG-PEG2000-NH_2_ (50 mg, 0.0199 mmol) and triethylamine (67 μL). The reaction was stirred overnight at 25 °C under an inert atmosphere. The product was purified by heptane precipitation, rotary evaporation, and dialysis as described above. DMG-PEG2000-glucose was obtained after lyophilization and stored as 10 mM dry DMSO stock at −80 °C until use. The product was identified by TLC and characterized by ^1^H NMR.

#### DMG-PEG2000-cocaine

Cocaine hydrochloride (C5776; Sigma-Aldrich; 200 mg, 0.588 mmol) was stirred in 9 mL of phosphate buffer (KH_2_PO_4_, 1.12 g; K_2_HPO_4_, 7.30 g; pH 7.4) and 1 mL of THF at 80 °C for 3 h. Isopropanol (200 mL) was added, and the product was evaporated under reduced pressure. The residue was refluxed in dichloromethane (20 mL) for 1 h and then concentrated to obtain benzoylecgonine. Benzoylecgonine (18.3 mg, 0.0595 mmol) was dissolved in 2.5 mL of DMF by sonication at 45 °C. EDC (40.0 mg, 0.209 mmol) and NHS (20.6 mg, 0.179 mmol) were dissolved in 1 mL of chloroform and 50 μL of DMF, and then added to the reaction. The reaction was stirred overnight at 25 °C. The activated product was reacted with DMG-PEG2000-NH_2_ (50 mg, 0.0199 mmol) and triethylamine (3 μL) overnight at 25 °C in an inert environment. The product was purified by heptane precipitation, rotary evaporation, and dialysis as described above. DMG-PEG2000-cocaine was obtained after lyophilization and stored as 10 mM dry DMSO stock at −80 °C until use. The product was identified by TLC and characterized by ^1^H NMR.

#### DMG-PEG2000-memantine

3,5-Dimethyladamantane-1-carboxylic acid (C4382; Sigma-Aldrich; 16.6 mg, 0.0796 mmol) was dissolved in 1 mL of DMF, then EDC (22.9 mg, 0.119 mmol) and NHS (12.1 mg, 0.119 mmol) were added. The reaction was stirred overnight at 25 °C under an inert atmosphere. The activated product was added to 0.5 mL of chloroform containing DMG-PEG2000-NH_2_ (50 mg, 0.0199 mmol) and triethylamine (3 μL). The reaction was stirred overnight 25 °C. The product was purified by heptane precipitation, rotary evaporation, and dialysis as described above. DMG-PEG2000-memantine was obtained after lyophilization and stored as 10 mM dry DMSO stock at −80 °C until use. The product was identified by TLC and characterized by ^1^H NMR.

#### DMG-PEG2000-acetylcholine

Carbamylcholine chloride (108240050; Holland Moran, Israel; 40 mg, 0.219 mmol) was dissolved in 4 mL of DMSO, then glutaric anhydride (G3806; Sigma-Aldrich; 22.7 mg, 0.199 mmol) and triethylamine (37 μL) were added. The reaction was stirred overnight at 80 °C. After evaporation, the product was redissolved in 0.35 mL of methanol and 0.8 mL of chloroform, then EDC (24.2 mg, 0.126 mmol) and NHS (12.7 mg, 0.126 mmol) were added. The reaction was stirred overnight at 25 °C under an inert atmosphere. After evaporation, the activated product was reacted with DMG-PEG2000-NH_2_ (50 mg, 0.0199 mmol) and triethylamine (30 μL) in 2 mL of DMSO overnight at 25 °C under an inert atmosphere. The product was purified by evaporation and dialysis as described above. DMG-PEG2000-acetylcholine was obtained after lyophilization and stored as 10 mM dry DMSO stock at −80 °C until use. The product was identified by TLC and characterized by ^1^H NMR.

#### DMG-PEG2000-methylphenidate

Ritalinic acid (602647; Sigma-Aldrich; 17.45 mg) was dissolved in 1.5 mL of DMF, and 2 M HCl was added dropwise until dissolution. EDC (45.8 mg, 0.238 mmol) and NHS (27.4 mg, 0.238 mmol) were added, and the pH was adjusted to 4–5. The reaction was stirred overnight at 25 °C. The activated product was added to 0.5 mL of chloroform containing DMG-PEG2000-NH_2_ (50 mg, 0.0199 mmol), and the pH was adjusted to 8–9 with triethylamine. The reaction was stirred overnight at 25° under an inert atmosphere. The product was purified by heptane precipitation, rotary evaporation, and dialysis as described above. DMG-PEG2000-methylphenidate was obtained after lyophilization and stored as 10 mM dry DMSO stock at −80 °C until use. The product was identified by TLC and characterized by ^1^H NMR.

#### DMG-PEG2000-nicotine

*trans*-4-Cotininecarboxylic acid (347574; Sigma-Aldrich; 150 mg, 0.681 mmol) and triethylamine (285 μL) were dissolved in 6 mL of DMF, and then *N*,*N*′-disuccinimidyl carbonate (DSC) (225827; Sigma-Aldrich; 191.9 mg, 0.749 mmol) was added. The reaction was stirred overnight at room temperature under an inert atmosphere. The activated product reacted with DMG-PEG2000-NH_2_ (50 mg, 0.0199 mmol) in 0.5 mL of chloroform overnight at 25 °C under an inert atmosphere. The product was purified by heptane precipitation and rotary evaporation. Then it dissolved in 5 mL of DMF and 10 mL of H_2_O and dialyzed as described above. DMG-PEG2000-nicotine was obtained after lyophilization and stored as 10 mM dry DMSO stock at −80 °C until use. The product was identified by TLC and characterized by ^1^H NMR.

### BT-LNP Fabrication and Characterization

Initially, we attempted to formulate the targeted LNPs using the classic vortex mixing method (Table S1).^91^ This method is straightforward to implement and effective for standard LNP formulations at a laboratory scale. However, for the targeted LNPs, which required dissolving lipids in DMSO, vortex mixing posed significant challenges in particle creation, likely due to the uncontrollability of the mixing parameters. Consequently, we adopted the microfluidic mixing method, which offers notable advantages, including reproducibility, uniformity of LNPs, and precise control over mixing parameters.^91^ This control allows us to modulate LNP properties such as size and consistency across a broad scale range. After optimizing the weight ratio of ionizable lipid to mRNA, we selected a 26.5:1 ratio, which provided the best size and polydispersity index (PDI) parameters while allowing us to use the maximum mRNA amount (60 μg) (Tables S1 and S2).

A total lipid mixture of 8-[(2-hydroxy­ethyl)­[6-oxo-6-(undecyl­oxy)­hexyl]­amino]­octanoic acid, 1-octylnonyl ester (SM-102) (2089251-47-6; Tzamal D-Chem Laboratories Ltd., Israel), 1,2-dioleoyl-*sn*-glycero-3-phosphoethanolamine (DOPE) (565600; Lipoid, Germany), cholesterol (C8667; Sigma-Aldrich), 1,2-dimyristoyl-*sn*-glycero-3-methoxypolyethylene glycol l000 (DMG-PEG1000) , (001317-1K; Biopharma PEG, USA), and 1,2-dimyristoyl-*rac*-glycero-3-polyethylene glycol 2000 amine (DMG-PEG2000-NH_2_) (DMG-PEG2000-AM; Nanocs, USA) in molar percentages of 50.25:10.05:38.19:1.01:0.5, was dissolved in ethanol or DMSO solvents (the organic phase); ethanol was only used for untargeted and glucose-LNP fabrication. To prepare untargeted-LNPs, DMG-PEG2000 (001317-2K; Biopharma PEG, USA) was used instead of DMG-PEG2000-NH_2_. mRNA (L-7211/L-7203/L-7202; Syntezza, Israel) was dissolved in 10 mM citrate buffer (pH 4.5) to produce an aqueous phase. Using the NanoAssembler Ignite (NIN0001; Cytiva, USA; provided by the A. Zinger lab, Technion), a microfluidic device, the organic and aqueous phases were combined at a 1:5 volumetric ratio and flow rate of 12 mL/min and diluted in a 1:1 volume ratio in PBS to produce LNPs. LNPs were then dialyzed against PBS (pH 7.4; 1:1000 volume ratio) using a 3.5–5 kDa dialysis membrane (133198; Repligen, USA) to change the organic solvent to water-based buffer at 4 °C for 24 h. The buffer was exchanged three times during the 24 h. LNPs that were used for CryoTEM imaging and *in vivo* experiments were downstream processed and concentrated to the desired RNA concentration using 100 kDa MWCO Amicon Ultra filter units (UFC510024; Sigma-Aldrich). Fresh batches of LNPs were used for each experiment.

The physical characteristics of LNPs, including mean size diameter (nm), poly dispersity index (PDI), and zeta potential (mV), were measured using dynamic light scattering with a Zetasizer Ultra (Malvern, UK). To measure mRNA encapsulation efficiency, the Quant-iT Ribogreen RNA Assay K\1 kit (R11490; Thermo Fisher, Rhenium, Israel) was conducted. LNPs were diluted 1:50 in either TE buffer or 1% Triton X-100 (93443; Sigma-Aldrich) in TE buffer, plated on a 96-well plate, and incubated at 37 °C for 10 min to induce lysis. RiboGreen reagent was then added to each well. After 5 min at 25 °C of incubation, the fluorescence intensity was measured on a plate reader (Tecan, Switzerland) at an excitation/emission of 485/528 nm. LNP mRNA encapsulation efficiency (%) was calculated by subtracting the unencapsulated mRNA fluorescence intensity value (intact LNPs in TE buffer) from total mRNA fluorescence intensity value (lysed LNPs in Triton) and then this value was divided by the total mRNA fluorescence intensity.

### BT-LNP Stability Examination

As a representative formulation, acetylcholine-LNPs and untargeted-LNPs were prepared to examine particle stability. Parameters such as particle size, PDI, and mRNA encapsulation efficiency were evaluated. BT-LNPs and UT-LNPs were prepared using microfluidic mixing and then divided into two storage groups (*n* = 3 per group): one stored at 25 °C and the other at 4 °C. Acetylcholine-LNPs samples were measured every 5 days, while untargeted-LNPs (of the 4 °C group) were measured only on day 1 and day 21. The mean particle diameter (nm) was measured using dynamic light scattering with a Zetasizer Ultra (Malvern, UK). mRNA encapsulation efficiency was determined using the RiboGreen RNA Assay Kit.

### Cryo-TEM

As a representative formulation, glucose-LNPs were imaged using cryo-TEM. A concentration of 10^12^ particles/mL (measured by a Zetasizer Ultra) was used for the imaging measurement. Vitrified specimens were prepared in a closed chamber equilibrated at 25 °C and near water saturation (>95% relative humidity). For each sample, a small drop of ∼6 μL was placed on a perforated grid (Ted Pella). Thin sample films were prepared by blotting excess liquid, and the blotted grid was plunged into liquid ethane at its freezing point (−183 °C) to create a vitrified specimen and transferred to liquid nitrogen (−196 °C) for storage. Cryo-EM analysis was performed while maintaining temperatures below −175 °C during transfer and imaging. The analysis was performed with a Tecnai T12 G2 TEM (FEI, Netherlands). Images were recorded at low dose irradiation by Digital Micrograph (Gatan, UK) on a Gatan US1000 2kx2k high-resolution cooled CCD camera using procedures developed in the D. Danino laboratory.^92^

### Development of the Graph Neural Network-Based Model

In this study, we deployed a neural network model developed to predict molecular activity in human tissues by utilizing data from various organisms, following a curriculum learning strategy. At the core of the model is a graph neural network (GNN), which processes molecular data by converting molecules into graph structures.

Consider a molecular graph, denoted as *G* = (*V*, *E*), in which *V* signifies the set of nodes and *E* comprises the set of edges. In this context, each node in a molecular graph represents a chemical atom, while each edge signifies a chemical bond between two atoms. Given a collection of molecular graphs *G* = {*G*_1_, ···, *G*_*N*_} and their corresponding labels *Y* = {*y*_1_, ···, *y*_*N*_}, our model’s primary objective is to learn a molecular representation vector that predicts the label, indicating whether a molecule is active or inactive within the specific tissue it is trained on, for each *G*_*i*_ ∈ *G*. This learning process involves the development of a mapping function, denoted as *f*_θ_: *G* → *Y*.

A GNN model capitalizes on both the inherent graph structure and node/edge features to generate a representation vector *h*_*v*_ for each node *v* ∈ *V*. More precisely, GNN employs a neighborhood aggregation function, which iteratively updates the node’s representation by aggregating the representations of its neighboring nodes and edges. After undergoing *l* iterations, a node representation *h*_*v*_^*l*^ effectively captures the information encapsulated within its *l*-hop neighborhoods.

In the initial layer of the GNN, we initialize the representations of both nodes and edges using their specific attributes within the molecular graph. Node attributes include the atom number (AN) and chirality tag (CT), while edge attributes encompass the bond type (BT) and bond direction (BD), as done by Guo et al.^93^ Formally, the node representation commences as *h*_*v*_^0^ = *v*_AN_ ⊕ *v*_CT_ and the edge representation as *h*_*e*_^0^ = *e*_BT_ ⊕ *e*_BD_, where *v* and *e* represent node and edge attributes, respectively, and ⊕ represents the concatenation operator.

Subsequently, the node representation *h*_*v*_^*l*^ at the *l*th layer of the GNN is formulated as follows:

$$h_{N\left(v\right)}^{l}={\mathrm{AGG}}_{l}\left(\left\{h_{u}^{l-1}:\forall u\in N\left(v\right)\right\},\left\{h_{e}^{l-1}:e=\left(v,u\right)\right\}\right)$$

$$h_{v}^{l}=\sigma \left(W^{l}\cdot \mathrm{Concat}\left(h_{v}^{l-1},h_{N\left(v\right)}^{l}\right)\right)$$

where *N*(*v*) represents the neighbor set of *v*, σ(·) denotes a nonlinear activation function, and AGG(·) stands for an aggregating function. We employ the graph isomorphism network (GIN),^94^ a model that has demonstrated state-of-the-art performance across various benchmark tasks.

Following this, we obtain the representation of each node within the molecular graph, denoted as

$$h_{v}=\frac{h_{v}^{l}}{∥h_{v}^{l}{∥}_{2}}$$

To derive the graph-level representation *h*_*G*_ for a given molecular graph, we calculate the average node embeddings at the final layer using

$$h_{G}=\mathrm{Mean}\left(\left\{h_{v}^{l}:v\in V\right\}\right)$$

This graph-level molecular representation, *h*_*G*_, is then fed into a classifier, such as a multilayer perceptron, to facilitate molecular activity prediction.

To optimize our model’s performance and harness universal representations, we integrate the pretrained graph neural network technique^95^ for initializing the parameters of the molecular GNN. This approach offers improved parameter initialization, aids in mitigating overfitting, and aligns with the advantages conferred by pretraining in various domains, such as natural language processing, computer vision, and graph analysis.

To further enhance molecular representations, our AI model incorporates a bond reconstruction loss and an atom-type prediction loss in addition to the conventional activity prediction loss. The bond reconstruction loss focuses on learning the presence or absence of chemical bonds between atoms within molecular graphs, using both positive edges (representing actual bonds) and negative edges (pairs of atoms without a bond). The atom-type prediction loss aims to identify the types of atoms present in a molecule by examining contextual subgraphs and the interactions of neighboring atoms. These losses are paired with the activity prediction loss, which uses a Multi-Layer Perceptron (MLP) to predict molecular activity based on graph-level representations of the molecule. The joint loss function combines these components, enabling the model to gain a deeper understanding of molecular structure and behavior, which in turn enhances its capacity to predict molecular activity across tissues.

Furthermore, we leverage a curriculum learning strategy to expose the model to data from different organisms in a sequential manner. Training begins with simpler species, such as rats, and gradually advances to more complex species like humans. This progressive training approach helps the model generalize predictions across species by adapting to increasing biological complexity. For model training, we utilized a comprehensive in vitro dataset sourced from the Open TG-GATEs^96^ and DrugMatrix^97^ repositories, accessible via https://ui.staging.kit.cloud.douglasconnect.com/datasets.

This dataset provides detailed information on molecules, specific tissues tested, involved organisms, test specifics, and the resulting activity status (active or inactive). The dataset can be conceptualized as tuples of molecule, tissue, organism, (activity/inactivity), where ’activity’ signifies a positive interaction between the molecule and a protein in the tested tissue of the organism. Although this dataset includes information across various tissues, we focused solely on data relevant to brain tissues for our training purposes.

To further specialize the AI model for predicting BBB passage, we fine-tuned the model using the BBBP dataset.^52^ This additional training refined the model’s ability to understand the specific factors influencing BBB permeability, thereby enhancing its performance in predicting BBB passage for both rat and human data.

The code and datasets are publicly available: 10.5281/zenodo.13863512.

#### Implementation Details

The AI model is based on the graph isomorphism network (GIN).^94^ Specifically, it employs the supervised-pretrained GIN model.^95^ The architecture consists of five GIN layers, each with a fixed embedding dimension of 300. Dropout is set to 0.5 to reduce overfitting. The learning rate is initialized at 0.001.

The initial activity dataset was partitioned into an 80–10–10 train–validation–test split, and the model checkpoint with the highest AUC over 1000 training epochs was retained. For fine-tuning on the blood–brain barrier permeability (BBBP) task, the dataset was again split into 80–10–10, and the checkpoint achieving the highest AUC on the validation set across 20 epochs was selected for evaluating the molecules examined in this study.

### Cell Culture

Each cell line was cultured at 37 °C in a humidified atmosphere containing 5% CO_2_, and fresh medium was added every 2–3 days.

hCMEC/D3 immortalized human brain capillary endothelial cells (Merck, USA) were provided by A. Sosnik (Laboratory of Nanomaterials Science, Department of Materials Science and Engineering, Technion). Cells (adherent) were cultured in EndoGRO-MV Complete Media Kit (SCME004; Merck Millipore, USA), supplemented with 1 ng/mL FGF-2 (GF003; Merck Millipore). Cell plating was performed on a flask coated with collagen type I, rat tail (08115; Merck Millipore) solution in PBS (BSS-1005-B; Merck Millipore) at a dilution of 1:20 and then incubated for 1 h at 37 °C. Then, trypsin-EDTA (SM2003C; Merck Millipore) was used for cell dissociation.

SH-SY5Y (ATCC), a thrice-cloned subline of the neuroblastoma cell line SK-N-SH, was provided by Prof. A. Fishman (Laboratory of Molecular and Applied Biocatalysis, Faculty of Biotechnology and Food Engineering, Technion). Cells (adherent) were cultured in a complete media comprising a 1:1 mixture of Dulbecco’s Modified Eagle’s Medium (DMEM) (D5796; Sigma-Aldrich) and Nutrient Mixture F12 HAM with sodium bicarbonate (N4888; Sigma-Aldrich), supplemented with 1% (v/v) penicillin (10,000 units/mL), streptomycin (10 mg/mL; Pen-Strep) (030311B; Biological Industries, Israel), 1% (v/v) amphotericin B (Amp-B) (2.5 mg/mL) (030281B; Biological Industries), 10% (v/v) FBS, and 1% (v/v) nonessential amino acids (013401B; Biological Industries). In general, cells were dissociated and harvested using a cell scraper.

For neuronal differentiation, SH-SY5Y cells were seeded on 1% gelatin from porcine skin, gel strength 300, Type a (G2500; Sigma-Aldrich) coated 96-well plates, followed by incubation in complete media supplemented with 10 μM all-trans retinoic acid (RA) (R2625; Sigma-Aldrih) for 4 days. Then, the medium was replaced with a starvation media (complete media without FBS), supplemented with 50 ng/mL human BDNF factor (4500210; PeproTech, Israel) for an additional 4 days; the cells were fully differentiated after 7 days.

Induced pluripotent stem cells (iPSCs) were maintained in Nutristem hPSC XF media (05-100-1A; Sartorius) on 100 μg/mL Matrigel basement membrane matrix (354234; Corning) diluted in Knockout DMEM (10829018; Gibco) or DMEM/Ham’s F12 (L0093-500; Biowest) coated cell culture plates. The media was changed daily, and at 80% confluency, iPSCs were passaged using EZ-LiFT Stem Cell Passaging Reagent (SCM139; Sigma-Aldrich) according to the manufacturer’s instructions.

For transwell-model experiments, human brain microvascular endothelial cells (BMECs) were differentiated from iPSCs (BGU019i), as described by Hollmann et al.^98^ and Sela et al.^36^ iPSCs were seeded on Matrigel-coated 6-well plates (354234; Corning) at a density of 3 × 10^5^ cells per well in Nutristem. The following day, the media was changed to DMEM/Ham’s F12 medium, supplemented with 20% knockout serum (10828010; Gibco), 1% (v/v) MEM Non-Essential Amino Acids Solution (NEAA) (01-340-1B; Sartorius), 0.5% (v/v) GlutaMAX (35050038; Gibco-ThermoFisher Scientific), 0.1 mM 2-mercaptoethanol, and 1% (v/v) penicillin–streptomycin solution (03-031-1B; Sartorius). The medium was changed daily for 6 days. Then the medium was switched to endothelial serum-free medium (11111044; Gibco-ThermoFisher Scientific), supplemented with 20 ng/mL basic fibroblast growth factor (bFGF, 100–18B-250; PeproTech), 10 μM retinoic acid (RA) (R2625; Sigma-Aldrich), 1% (v/v) B27 (17504044; Gibco-ThermoFisher Scientific), 1% (v/v) penicillin–streptomycin solution, for 2 days.

On day 8 of the differentiation, BMECs were detached and reseeded at density of 1 × 10^5^ cells onto 3 μm pore polyester transwell inserts in a 24-well plate (662630; Greiner AG), precoated with 100 μg/mL human collagen type IV (C5533; Sigma-Aldrich) and 100 μg/mL bovine fibronectin (F1141; Sigma-Aldrich). Cells were maintained in the same medium composition as used on day 6. The medium was replaced with the same formulation as day 6, the following day, but without RA and bFGF. The barrier function was evaluated by transepithelial/transendothelial electrical resistance (TEER) measurements (Millicell ERS-2 voltohmmeter, Merck Millipore) daily. On day 12 of the differentiation, an average TEER above 1000 Ω·cm^2^ was chosen as the optimal value to start the experiments.

The human iPSCs (male WTC11 background, with stably integrated doxycycline-inducible NGN2 transgene) line generated i3 neurons based on a previously published protocol.^99^ Briefly, iPSCs were seeded on Matrigel-coated six-well plates at a density of 1.5 × 10^6^ cells per well in DMEM/F12 medium with 1% (v/v) N-2 supplement (17502048; Gibco-ThermoFisher Scientific), 1% (v/v) GlutaMAX, 1% (v/v) NEAA, supplemented with 2 μg/mL doxycycline and 10 μM ROCKi. The next day and the following, the media was changed with the same composition without ROCKi. On day 3 the cells were detached with tryplE (Gibco-ThermoFisher Scientific) and were frozen in Nutrifreeze (Sartorious). For the in vitro BBB model experiment, thawed neurons were seeded on 24-well plate glass bottom (p24-1.5H-N, Cellvis) precoated with 30 μg/mL poly-l-ornithine (PLO, P4957, Sigma-Aldrich) and 10 μg/mL laminin (23017015; Gibco-ThermoFisher Scientific) in a maturation medium consisting of Neurobasal-A (10888022; Gibco-ThermoFisher Scientific), 2% B27, 10 ng/mL BDNF (450-02-50; PeproTech), 10 ng/mL NT-3 (450-03-50; PeproTech), and 1 μg/mL laminin. The medium was changed twice a week. After 6 days, NGN2 neurons were cocultured with BMECs seeded on a transwell.

For acetylcholine LNPs receptor binding assays, HEK 293T cells (American Type Culture Collection) in passage numbers 12–20 were cultured in DMEM supplemented with 10% FBS, 1% l-glutamic acid, and 1% penicillin–streptomycin at 37 °C in 5% CO_2_ and transfected with 3 μg of AchLightG^74^ plasmids in 35 mm plates (Lipofectamine 3000 Transfection Reagent). Imaging was performed 24 h following transfection in external Tyrode solution (119 mM NaCl, 5 mM KCl, 25 mM HEPES, 2 mM CaCl_2_, 2 mM MgCl_2_, 33 mM glucose) at pH 7.4.

### Generation of Human Cortical Organoids

The human iPSCs line BGU1110iHC was used to generate cortical organoids according to a combination of two previously published protocols by Martins-Costa et al. and Rosebrock et al.^100,101^ Briefly, iPSCs were seeded at density of 9000 cells/well in low attachment U-shape 96 well plates (650970; Greiner Bio-One) in hESCs media composed of DMEM/Ham’s F12, 20% (v/v) Knockout Serum Replacement, 0.5% (v/v) GlutaMAX, 1% (v/v) NNEA, 1% (v/v) penicillin–streptomycin solution (03-031-1B; Biological Industries Israel Beit-Haemek Ltd.), and 5 μM 2-mercaptoethanol with the addition of 4 ng/mL human bFGF and 50 μM ROCKi.

On day 2, media was changed to hESC media containing 10 μM SB431542 (1614; Tocris), 250 ng/mL LDN193189 (SML0559; Sigma-Aldrich), and 3.3 μM XAV939 (3748; Tocris). On day 4, the media was changed to N-2 media containing DMEM/Ham’s F12, 1% (v/v) N-2 supplement, 1% (v/v) GlutaMAX, 1% (v/v) NEAA, 1% (v/v) penicillin–streptomycin solution with the addition of the molecules from day 2. The media was changed every 2 days, and on day 10, 2% Matrigel was added. On day 13, the media was changed to N-2/NB media containing 50% (v/v) Neurobasal (21103; Gibco-ThermoFisher Scientific), 50% (v/v) DMEM/Ham’s F12, 0.5% (v/v) N-2 supplement, 1% (v/v) B27 supplement minus vitamin A (12587010; Gibco- ThermoFisher), 1% (v/v) GlutaMAX, 1% (v/v) NEAA, 1% (v/v) penicillin–streptomycin solution, and 5 μM 2-mercaptoethanol. On day 15, the media was changed to N-2\NB media containing 1% (v/v) B27 instead of B27 minus vitamin A. On day 17, the organoids were transferred into cell-repellent 24-well plates (662970; CELLSTAR) and placed on an orbital shaker. Afterward, the media was changed twice a week.

### *In Vitro* LNP Viability and Transfection Efficiency

For the BT-LNP library screening and viability studies, hCMEC/D3 cells were seeded (65,000 cells/well) 1 day before the experiment. SH-SY5Y cells were seeded (65,000 cells/well) and differentiated 1 week before the experiment. On the experiment day, both types of cells were treated at a dose of 200 ng luciferase mRNA (L-7202; Trilink BioTechnologies, US) LNPs for 16 h. To measure luciferase expression, 20 μL of ONE-Glo Luciferase Assay System (E6110; Promega, US) was added to the media. Finally, the luminescent signal of the plates was measured using a microplate reader. The relative light units (RLUs) obtained from BT-LNPs were normalized against the RLU of untargeted LNPs.

To measure cell viability after LNP treatment, the PrestoBlue (A13261; Thermo Fisher, USA) assay was performed according to the manufacturer’s protocol. Fifteen minutes after adding the reagent to the hCMEC/D3 wells and the SH-SY5Y wells, the fluorescence signal (535/590 nm) was measured using a microplate reader. The measurements from the media-only group were averaged and subtracted from all other values. Subsequently, the values for each cell treatment were normalized to those of the untreated cells (control group).

### *In Vivo* Biodistribution Studies

All animal experiments were approved by the Inspection Committee on the Constitution of the Animal Experimentation at the Technion (IL0300123H, IL1110623H and IL1631224) and conducted according to its stipulated regulations. For luciferase mRNA LNPs, healthy male adult C57BL/6 mice (Envigo, Israel) were injected via the lateral tail vein with LNPs at a dose of 0.729 mg/kg mRNA. After 6 h, mice were euthanized, perfused with PBS, and then dissected to collect organs. The organs were then placed on dry ice and kept at −80 °C until homogenization took place. The organ tissues were cut into small pieces, and a lysis buffer was added to the samples. For the brain samples, 2 mL of a prepared lysis buffer (100 mM Tris-HCl, 2 mM EDTA, 0.1% Triton X-100, pH 7.8) was added. For the remaining organ samples, a commercial lysis buffer (E1531; Promega, US) was used: 4.5 mL for liver samples, 2 mL for kidney samples, and 1 mL for heart, lung, and spleen samples. Then the samples were physically dissociated using a gentleMACS device (Protein 01 program; Miltenyi Biotec, US). The samples’ lysate was centrifuged at 12,000*g* for 10 min at 4 °C. To 100 μL of the supernatant from each sample, 20 μL of the ONE-Glo Luciferase Assay System was added, and the luciferase activity was recorded for 1000 ms using a microplate reader. Finally, the samples’ protein concentration was determined using a Bradford Protein Assay Kit (5000201; BIO-RAD, Israel). Luciferase activity is presented as RLUs per milligram of total protein normalized to untargeted-LNPs values.

For the *in vivo* kinetics study, healthy adult male C57BL/6 mice were injected via the lateral tail vein with glucose-LNPs at a dose of 0.729 mg/kg mRNA. At 6 h, 2 days, and 6 days postinjection, the mice were euthanized, perfused with PBS, and their livers, brains, and spleens were extracted. The organs were placed on dry ice and stored at −80 °C until the luciferase assay, as described above, was performed.

### Evaluation of BT-LNPs *In Vitro* BBB Passage

To assess the transport of selected BT-LNPs (acetylcholine) across the BMEC barrier, transwells containing BMECs in the upper chamber (day 12 of differentiation) were placed on top of human iPSC-derived neurons cultured in the lower compartment. Either acetylcholine-targeted or untargeted LNPs encapsulating mCherry mRNA were added to the upper (BMEC-facing) chamber at a final concentration of 400 ng mRNA/well and incubated for 24 h.

Following incubation, both compartments were fixed with 4% paraformaldehyde (PFA) for 10 min at room temperature and washed three times with PBS (5 min each). Cells were permeabilized using 0.1% Triton X-100 for 10 min, followed by blocking in a solution containing 2% normal goat serum (NGS) (005-000-121; Jackson ImmunoResearch) and 1% bovine serum albumin (BSA) (A4503; Sigma-Aldrich) for 1 h at room temperature. Cells were then incubated overnight at 4 °C with the following primary antibodies, diluted in 1% NGS and 0.1% BSA: rabbit anti-ZO-1 (13663; Cell Signaling Technology), rabbit anti-β-Tubulin III (T2200; Sigma-Aldrich), and chicken anti-mCherry (ab205402; Abcam). The next day, cells were washed three times with PBS and incubated with goat anti-rabbit Alexa Fluor 488 (A11034; ThermoFisher Scientific) and goat anti-chicken Alexa Fluor 647 (A32933; ThermoFisher Scientific) secondary antibodies for 1 h at room temperature. After final washes with PBS (3 × 5 min), DAPI Fluoromount-G (0100-20; SouthernBiotech) was applied for nuclear staining and mounting. Imaging was performed using a confocal microscope (Olympus FV3100-IX-83) with 405, 488, and 640 nm lasers. Image adjustments for representative figures were performed uniformly using ImageJ, applying standardized brightness and contrast settings.

It should be noted that while ZO-1 staining appears discontinuous in some regions, the BMEC monolayers maintained high transendothelial electrical resistance (TEER) values (average > 1000 Ω·cm^2^), consistent with an intact and functionally competent barrier phenotype.

### *In Vivo* Cre mRNA Delivery

Male Ai9 mice (B6.Cg-Gt­(ROSA)­26Sor^tm9(CAG‑tdTomato)Hze^/J), originally sourced from The Jackson Laboratory, were obtained from an institutionally managed animal colony (Ethical approval IL0290123). These mice were intravenously administered LNPs encapsulating mRNA encoding Cre Recombinase (L-7211; Trilink BioTechnologies, US) at a dose of 0.729 mg/kg. After 2 days, the mice were sacrificed and perfused with either only PBS (for flow cytometry) or with PBS followed by 4% PFA in PBS (for histological and immunofluorescence analysis). The brains were then extracted for further processing.

### Cell Isolation and Staining for Flow Cytometry

To test the tdTomato+ cells in brain cell types, cell isolation and staining were performed after 2 days of treatment with Cre mRNA formulations (0.729 mg/kg), and then the cells were analyzed by flow cytometry.

The extracted brain tissues were cut into small pieces and enzymatically and physically dissociated using a gentleMACS device and an Adult Mouse Brain Dissociation Kit (mouse and rat) (130107677; Almog Diagnostics, Israel), according to the kit’s dissociation protocol.

The dead cells were removed from cell samples using a Dead Cell Removal Kit (130090101; Almog Diagnostics), according to the manufacturer’s instructions. The live-single-cell suspensions were counted in Beckman Coulter Z2 Cell Counter (provided by the T. Shlomi lab, Technion) and then stained with a panel of antibodies: Brilliant Violet 510 Anti-Mouse/Human CD44 (BLG-103044), Brilliant Violet 711 Anti-Mouse CD45 (BLG-103147), Brilliant Violet 421 Anti-Mouse CD31 (BLG-102424), PE/Cyanine7 Anti-Mouse/Human CD11b (BLG-101215) (all these antibodies were purchased from BioLegend, US), Anti-Mouse CD24 Antibody Clone M1/69 Alexa Fluor 488 (STEMCELL, US), ACSA-2 Antibody Anti-Mouse APC-Vio770 REAfinity (130-116-247; Miltenyi Biotec, Us), and O4 Antibody Anti-Human/Mouse/Rat APC REAfinity (130-119-982; Miltenyi Biotec, US). Additionally, 10 μL of BD Horizon Brilliant Stain Buffer Plus (566385; BD Biosciences, US) was added to each staining tube. The dilution of each antibody was determined according to the manufacturer’s instructions. The cells were incubated with antibodies for 30 min on ice in the dark. Subsequently, cells were washed once with PBS and resuspended with Zombie Red (BLG-423109; BioLegend, US) regent according to the manufacturer protocol. Cells were incubated with the reagent for 15 min at 25 °C. Finally, cells were washed with FACS buffer (2% FBS in PBS) and resuspended with FACS buffer before reading.

All cell groups were measured and analyzed using Cytek Aurora (Cytek Biosciences; US). Each antibody was used for single staining. A minimum of one million cells were recorded for each test sample. In the mean fluorescence intensity (MFI) graphs (Figure 4C), the final signal values were calculated by subtracting the “basal” values from the untreated mice (control group).

### *Ex Vivo* Fluorescence Imaging

Biodistribution, specifically for acetylcholine-targeted LNPs in the Ai9 mice model, was also evaluated using IVIS imaging in the brain and liver. LNPs encapsulating Cre mRNA were administered intravenously at a dose of 0.729 mg/kg to two groups of Ai9 mice. Tissues were exercised for either 48 h or 3 weeks postinjection. Brains were imaged using a 570 nm excitation wavelength and a 620 nm emission filter, with a binning factor of 8, an f-stop of 2, and an exposure time of 0.75 s. Livers were imaged using the same excitation and emission settings, with a binning factor of 2, an f-stop of 4, and an exposure time of 1 s. Quantitative analysis was performed using the region of interest (ROI) tool in Living Image software. To correct for background signal, the average radiance of tissues from a PBS-injected control mouse was subtracted from the respective radiance values of treated samples.

### LNPs Treatment and Immunofluorescence Staining of Cortical Organoids

On day 57 of differentiation, cortical organoids were incubated with acetylcholine or untargeted encoding mCherry LNPs for 48 h. The LNPs were applied at a concentration equivalent to 1 μg mRNA per organoid. After incubation, organoids were washed with PBS and fixed with 4% PFA at 4 °C overnight. The following day, organoids were washed with PBS (3 × 5 min) and incubated in 20% sucrose in PBS overnight at 4 °C. The next day, organoids were transferred to 30% sucrose in PBS at 4 °C overnight. Organoids were then embedded in OCT, snap frozen, and stored at −80 °C. Cryosections were cut at 20 μm thickness using a cryostat (Leica CM1950) and mounted on positively charged slides (BN9308C; Bar-Naor Ltd.). Sections were washed with PBS, permeabilized with 0.3% Triton X-100 (00738859; Sigma-Aldrich) for 15 min, and blocked in solution containing 5% NGS, 2% BSA, and 0.1% Triton X-100 for 1 h at room temperature. Sections were then incubated overnight at 4 °C with rabbit anti-β-Tubulin III (T2200; Sigma-Aldrich), diluted in 2% NGS, 1% BSA, and 0.1% Triton X-100. The next day, sections were washed with PBS (3 × 5 min) and incubated with goat antirabbit Alexa Fluor 488 (A11034; Thermo-Fisher) for 1 h at room temperature. After three PBS washes, slices were mounted with DAPI Fluoromount-G (0100–20; Southern Biotech). Image acquisition was performed using a confocal microscope (Olympus IX-83) using 405, 488, and 561 nm lasers. Brightness and contrast of representative images were uniformly adjusted in ImageJ software.

### Receptor Binding Mechanism Studies

To evaluate the specificity and mechanistic basis of acetylcholine-LNP cellular uptake, we performed inhibition studies using tight-junction-forming BMECs and SH-SY5Y human neuroblastoma cells. BMECs and SH-SY5Y were seeded in 24-well transwell inserts or 96-well plates, respectively, and allowed to reach appropriate confluence before treatment.

For receptor inhibition experiments, cells were pretreated for 30 min with the muscarinic acetylcholine receptor antagonist scopolamine (Scop) (2 mM), the nicotinic receptor antagonist mecamylamine (Mec) (10 μM), or a combination of both inhibitors. Following pretreatment, cells were exposed to FLuc-encoding acetylcholine-LNPs or untargeted LNPs. To further investigate the contribution of membrane microdomain integrity and endocytic pathway-based internalization of acetylcholine-LNP uptake, SH-SY5Y cells were pretreated with 4.5 mM methyl-β-cyclodextrin (MβCD), a caveolae-mediated endocytic inhibitor, 30 min before FLuc-encoding acetylcholine-LNPs or untargeted LNPs addition. Cells were incubated with the LNP formulations for 16–20 h at 37 °C.

To assess inhibitory effects, mRNA transfection efficiency was tested using ONE-Glo luciferase assay reagent as described above, with slight modifications for the BMECs experiment. Values were normalized to the untargeted LNPs control groups.

To further investigate the contribution of membrane microdomain integrity and endocytic pathway-based internalization of acetylcholine-LNP uptake, SH-SY5Y cells were pretreated with 4.5 mM MβCD 30 min before adding luciferase-encoding acetylcholine-LNPs or untargeted LNPs.

To exclude potential cytotoxic effects of the different inhibitors at the tested concentrations, a PrestoBlue viability assay was conducted as described above.

For receptor binding assessment in AchLightG-HEK293 experiments, acetylcholine-targeted LNPs were applied at a concentration of 10^8^ particles/μL in a total volume of 80 μL per experiment. For evaluation of acetylcholine-LNP–receptor interactions, FLuc-LNPs encapsulating 1 μg mRNA were used per well. Acetylcholine-LNPs encapsulating mCherry mRNA were employed to examine receptor engagement followed by transfection (1 μg mRNA per treatment). For continuous single-cell imaging experiments, cells were incubated with either acetylcholine-FLuc LNPs for ∼35 min or acetylcholine-mCherry-LNPs for 23 h at room temperature without removal. In other mCherry-acetylcholine-LNP experiments, cells were incubated under standard conditions until the time of imaging. Untargeted LNPs were used as experimental control.

### Biosensor Microscopy

We imaged fluorescence intensity using a 2pFLIM microscope, which was based on a Galvo–Galvo two-photon system (Thorlabs) and a 2pFLIM module (Florida Lifetime Imaging), equipped with a time-correlated single-photon counting board (Time Harp 260, Picoquant). The microscope was controlled, and fluorescence intensity was quantified via the FLIMage software (Florida Lifetime Imaging Microscopy, USA). For excitation, we used a Ti:sapphire laser (Chameleon, Coherent) at a wavelength of 960 nm to simultaneously excite AchLightG and mCherry. Excitation power was adjusted using a Pockels cell (Conoptics) to 1.0–2.0 mW. Emission was collected with a 16 × 0.8 NA objective (Nikon), divided with a 565 nm dichroic mirror (Chroma), with emission filters of 525/50 nm and 607/70 nm, detected with two photomultiplier tubes with low transfer time spread (H7422-40p, Hamamatsu). Images were collected by 128 × 128 or 256 × 256 pixels and movies by 256 × 256. Each image was acquired at 2 ms/line, averaged over 24 frames.

Software for quantification of the fluorescence intensity data in HEK293 cells is available on the R. Yasuda laboratory GitHub page (https://github.com/ryoheiyasuda/FLIMage_public/). The resulting raw data for analysis are available at 10.5281/zenodo.17034133.

### Fluorescence Immunohistochemistry and Image Analysis

Fluorescence immunohistochemistry was employed in two phases: first, to visually confirm the tdTomato expression of LNPs throughout the entire brain, and second, to explore the localized expression specifically within the cortical area.

The first group of male Ai9 mice received an intravenous injection of acetylcholine-LNPs encapsulating Cre mRNA (0.729 mg/kg). Two days later, the mice were anesthetized, sacrificed, perfused with ice-cold PBS followed by 4% PFA in PBS, and their brains were collected for the fluorescence immunohistochemistry process. Untreated mice served as the control group. Brain sections were imaged using a Nikon Eclipse Ti2 epifluorescence microscope (Nikon, Japan) equipped with appropriate filter sets. To minimize background autofluorescence and baseline tdTomato expression observed in untreated controls, all images were uniformly adjusted for brightness and contrast using Adobe Photoshop.

The second group of male Ai9 mice was anesthetized with 0.5% isoflurane and 1% O_2_ and received a unilateral stereotactic injection with either luciferase mRNA LNPs (as a control) or Cre mRNA (with untargeted-LNPs or acetylcholine-LNPs) into the cortex. This procedure was conducted using an automated stereotactic injection device (NBT–New Biotechnology Ltd., US) equipped with the mouse brain atlas. The LNP formulation was injected at a dosage of 0.17 μg/μL (0.2 μL/min, as a single injection). Injections were performed using a Hamilton Neuros syringe (Hamilton Company). After the infusion, the injector was left at the injection site for 5 min before being slowly withdrawn. A period of 2 days was allowed before perfusion and brain fluorescence immunohistochemistry analysis.

The brains were postfixed overnight in 4% PFA, washed twice with PBS, and then cryoprotected by 30% sucrose in PBS at 4 °C for 2–3 days. After cryoprotection, the brains were frozen in the O.C.T. compound (BN62550; Bar-Naor, Israel) and stored at–80 °C until further processing. The brain sections were obtained by a cryostat machine (Leica VT1200S Automated Vibrating Microtome, provided by the A. Zeisel lab). The slices were sectioned on the coronal plane at 50 μm, mounted on glass slides, and stored at–20 °C until further use.

Sections without further immunostaining (Figures 4D and S8) were mounted with mounting medium immune reagent (BN9990412, Bar-Naor, Israel).

Sections from the cortex area were immunostained and used for imaging analyses. Specifically, the sections were washed three times in a wash buffer (PBST) (PBS, 0.05% Triton X-100) and then blocked (2.5% goat serum (S-1012-20; Vector Laboratories, US)) at room temperature for 1 h. The sections were next incubated in antibody dilution buffer (PBS + 1% BSA) with primary antibodies (1:250 rabbit anti-Iba1 (ab178846; Abcam, US) or 1:250 rabbit anti-GFAP (ab68428; Abcam) overnight at 4 °C. The sections were washed three times in a wash buffer before incubation with a secondary antibody (1:500 goat anti-rabbit IgG H&L Alexa Fluor 488 (ab150077; Abcam)) for 2 h. Next, the sections were washed three times in a wash buffer and incubated in antibody dilution buffer with an additional primary antibody (1:1000 Alexa Fluor 647 anti-NeuN (EPR12763, Abcam)) overnight at 4 °C. Finally, the sections were washed three times, dried, mounted using a DAPI Fluoromount-G (010020; ENCO, Israel), covered-slipped, and stored at −80 °C until imaging.

The stained brain sections were representatively imaged with a CSU-W1 spinning disk confocal microscope (Nikon, Japan) equipped with 405, 488, 561, and 640 nm lasers for multichannel acquisition. A Nikon Eclipse Ti2 epifluorescence microscope with appropriate filter sets was used for image acquisition and further analysis. Representative images were uniformly adjusted for brightness and contrast using ImageJ software, without altering the original signal content.

Image analysis was performed to quantify the colocalization of tdTomato-positive cells with NeuN, GFAP, Iba1, and nuclei using a multistep process. The colocalization assessment was based on DAPI nuclear staining as a reference point, ensuring a nucleus-centered approach. Each DAPI-stained nucleus served as a reference point for evaluating overlapping signals from the red (tdTomato-positive cells), green (cell-specific markers: GFAP or Iba1), and yellow (NeuN marker) channels. Initial image segmentation was conducted using Roboflow,^102^ a machine learning-based computer vision platform. Following segmentation, a Python-based analysis was implemented to process the red, green, yellow, and blue fluorescent channel images. The images were converted into binary masks, where each nucleus was individually assessed for overlapping signals in the different fluorescent channels. This approach ensured precise quantification of cellular colocalization. To calculate the tdTomato colocalization ratio (%), logical operations were applied to determine overlap between fluorescent channels.

For the non-neuronal and non-astrocytes population and for the non-neuronal and non-microglia population, the colocalization ratio was obtained by excluding the overlap between the red and green channels (tdTomato and GFAP/Iba1 respectively) and the red and yellow channels (tdTomato and NeuN) from the total overlap of the red and blue (tdTomato and DAPI) channels. To refine the colocalization ratio for neurons, cells double-positive for yellow (NeuN) and green (GFAP/Iba1) were excluded from the total overlay of the red and yellow channels, preventing misclassification of neuronal tdTomato expression. The overlap ratios were compiled in Excel files for further statistical analysis. The complete computational workflow, including segmentation and colocalization quantification, is available at 10.5281/zenodo.13894885.

### Statistical Analysis

All values in HEK293 experiments are presented as the mean ± standard error of the mean (SEM). Statistical significance was tested by two-tailed *t* test for comparison of two groups or one-way analysis of variance (ANOVA) followed by *post hoc* Tukey’s multiple-comparison test for comparison of multiple groups. Sample sizes were not predetermined using statistical methods and were selected based on a previous similar experimental design.^103,104^ All other data were reported as mean ± standard deviation (SD). Comparisons were performed between distinct groups. Groups were analyzed by a two-tailed unpaired *t* test and ANOVA. Statistical significance was set as *p < 0.05, **p < 0.01, ***p < 0.001, and ****p < 0.0001 with a 95% confidence interval. Analysis and figures were generated using GraphPad Prism v. 10.2.3 (GraphPad Software, Inc., La Jolla, CA, USA).

## Supplementary Material











## Data Availability

All of the data needed to evaluate the conclusions in the paper are present in the paper and/or the . Correspondence and requests for materials should be addressed to A.S. (avids@technion.ac.il) and K.R. (kirar@cs.technion.ac.il).

## Notes

Optimization and characterization of LNPs, additional *in vitro* and *in vivo* data, representative flow cytometry gating scheme, image analysis strategy and controls (PDF)
Movie showing changes in AchLightG fluorescence intensity following acetylcholine-targeted LNP application in HEK293 cells (Movie S1) (AVI)
Movie showing changes in AchLightG fluorescence intensity following untargeted LNP application in HEK293 cells (Movie S2) (AVI)
Movie showing changes in AchlightG and mCherry fluorescence intensities following application of mCherry mRNA-encapsulating acetylcholine-targeted LNPs in HEK293 cells (Movie S3) (AVI)
^1^H NMR characterization of synthesized small molecule-PEG lipid conjugates (PDF)
