# Supplementary material for: AI-Validated Brain Targeted mRNA Lipid Nanoparticles with Neuronal Tropism
Source: ACS Nano. 2025 Sep 16;19(41):36106–28. doi: 10.1021/acsnano.4c15013 (PMC12548354; doi:10.1021/acsnano.4c15013)
Supplement: Supplementary file 1 [file nn4c15013_si_001.pdf]

Supporting Information

AI-Validated Brain Targeted mRNA Lipid Nanoparticles with Neuronal Tropism

Mor Sela[1 #], Gal Chen[1,2 #], Haim Kadosh[1,2 #], Tomer Kagan[3], Raneen Nicola[4], Sally Turutov[5], Yuval Richtman[1], Lin Zhige[6], Mia R. Albalak Menasherov[1,2], Shaked Kagan[1], Tzur Schroeder[7], Patricia Mora-Raimundo[1], Reaam Kablan[1,8], Egor Egorov[1], Anas Odeh[9], Tasneem Abu-Raiya[1], Inbal Ionita[10], Inbar Freilich[10], Galoz Kaneti[1], Ibrahim Knani[1], Yehuda Arav[11], Yael Leichtmann-Bardoogo[4], Keshet Tadmor[12], Jeny Shklover[1], Tommaso Patriarchi[13,14], Dganit Danino[10,15], Peleg Hasson[9], Uri Ashery[12,16,17], Amit Zeisel[6], Ben M. Maoz[4,12,17], Tal Laviv[3,16], Kira Radinsky[5,+] and Avi Schroeder[1, +]

[1]The Louis Family Laboratory for Targeted Drug Delivery and Personalized Medicine Technologies, Department of Chemical Engineering, Technion – Israel Institute of Technology, Haifa 3200003, Israel

[2]The Interdisciplinary Program for Biotechnology, Technion - Israel Institute of Technology, Haifa 3200003, Israel

[3]Department of Physiology and Pharmacology, Gray Faculty of Medical and Health Sciences, Tel Aviv University, Tel Aviv 6997801, Israel

[4]Department of Biomedical Engineering, Engineering Faculty, Tel Aviv University, Tel Aviv 6997801, Israel

[5]Faculty of Computer Sciences, Technion - Israel Institute of Technology, Haifa 3200003, Israel

[6]Faculty of Biotechnology and Food Engineering, Technion–Israel Institute of Technology, Haifa 3200003, Israel

[7]Student at the Faculty of Medicine, The Rappaport Faculty of Medicine and Research Institute, Technion – Israel Institute of Technology, Haifa, 3200003 Israel

[8]The Norman Seiden Multidisciplinary Program for Nanoscience and Nanotechnology, Technion – Israel Institute of Technology, Haifa 3200003, Israel

[9]Department of Genetics and Developmental Biology, The Rappaport Faculty of Medicine and Research Institute, Technion – Israel Institute of Technology, Haifa, 3200003 Israel

[10]CryoEM Laboratory of Soft Matter, Department of Biotechnology and Food Engineering, Technion - Israel Institute of Technology, Haifa 3200003, Israel

[11]Department of Applied Mathematics, Israel Institute for Biological Research, Ness-Ziona 7410001, Israel

[12]Sagol School of Neuroscience, Tel Aviv University, Tel Aviv 6997801, Israel

[13]Institute of Pharmacology and Toxicology, University of Zürich, Zürich CH 8006, Switzerland

[14]Neuroscience Center Zürich (ZNZ), University of Zürich, Zürich CH 8006, Switzerland

[15]bCryo-EM and Self-Assembly Laboratory, Guangdong-Technion - Israel Institute of Technology, Shantou 515063, China

[16]School of Neurobiology, Biochemistry, Biophysics, Life Sciences Faculty, Tel Aviv University, Tel Aviv 6997801, Israel

[17]Drimmer-Fischler Family Stem Cell Core Laboratory for Regenerative Medicine, Tel Aviv University, Tel Aviv 6997801, Israel

+**Corresponding authors** avids@technion.ac.il, kirar@cs.technion.ac.il

# These authors contributed equally

Supplementary tables and figures

Table S1. Size and PDI data optimization results for representative BT-LNP formulations. The final selected method conditions are detailed in the methods section. All data are presented as mean ± SD (n = 3).

BT-LNPs	A		B		C	
	Z-Average [nm]	PDI	Z-Average [nm]	PDI	Z-Average [nm]	PDI
Tryptophan-LNPs	4406±749	0.5222±0.0307	2675±209	1.302±0.046	142.9±7.5	0.2454±0.0118
Acetylcholine-LNPs	5327±81	0.4585±0.0523	8011±1527	1.924±0.021	503±58	0.6671±0.1131
Cocaine-LNPs	2563±1935	0.692±0.333	1685±603	0.8674±0.3010	1184±94	0.3645±0.1829
Memantine-LNPs	3757±959	1.478±0.286	7397±2612	1.338±0.3874	441±23	0.1750±0.2151
Norepinephrine-LNPs	2440±103	1.165±0.065	1739±778	0.9486±0.3294	797±82	0.6376±0.0467
Methylphenidate-LNPs	5339±2506	0.525±0.165	2596±269	1.132±0.1178	144.2±6.7	0.1940±0.0230
**BT-LNPs**	**D**		**E**		**F**	
	**Z-Average [nm]**	**PDI**	**Z-Average [nm]**	**PDI**	**Z-Average [nm]**	**PDI**
Tryptophan-LNPs	135.8±10.9	0.1378±0.0898	185.6±3.1	0.0805±0.0570		
Acetylcholine-LNPs	116.0±3.8	0.2099±0.0330	203.4±5.9	0.2787±0.0387		
Cocaine-LNPs	130.4±9.8	0.2812±0.0231	475±332	0.4713±0.0939	1284±220	0.5204±0.1109
Memantine-LNPs	119.7±5.1	0.2381±0.0113	1137±80	0.6838±0.1041	260.2±5.6	0.2763±0.0208
Norepinephrine-LNPs	135.7±3.4	0.2169±0.0569	783±395	0.7457±0.0746		
Methylphenidate-LNPs	120.9±4.8	0.4532±0.0434	952±138	0.3623±0.1931		

Method conditions:

A = Vortex mixing method; 1% molar of targeted-lipid; 14.7:1 w/w ratio of ionizable lipid to mRNA; lipid organic phase in Ethanol.

B = Vortex mixing method; 1% molar of targeted-lipid; 14.7:1 w/w ratio of ionizable lipid to mRNA; lipid organic phase in DMSO.

C = Microfluidic mixing method; 1% molar of targeted-lipid; 26.4:1 w/w ratio of ionizable lipid to mRNA; lipid organic phase in DMSO.

D = Microfluidic mixing method; 0.5% molar of targeted-lipid; 26.4:1 w/w ratio of ionizable lipid to mRNA; lipid organic phase in DMSO.

E = Microfluidic mixing method; 0.5% molar of targeted-lipid; 17.7:1 w/w ratio of ionizable lipid to mRNA; lipid organic phase in DMSO.

F = Microfluidic mixing method; 0.5% molar of targeted-lipid; 22.1:1 w/w ratio of ionizable lipid to mRNA; lipid organic phase in DMSO.

Table S2. mRNA encapsulation efficiency of BT-LNP library.
All data are presented as mean ± SD (n = 3 - 8).

Targeting Moiety	mRNA Encapsulation Efficiency [%]
Untargeted	91.68 ± 2.12
Glucose derivate	91.70 ± 1.13
Methylphenidate derivate	87.58 ± 8.05
Memantine derivate	89.20 ± 5.13
Acetylcholine derivate	90.09 ± 5.76
Cocaine derivate	90.72 ± 3.81
Tryptophan derivate	89.33 ± 2.55
Nicotine derivate	93.70 ± 1.98
Norepinephrine derivate	89.32 ± 3.05

Table S3. Targeting moieties and their relevant receptors/pathways.

Molecule	Targeted Receptor/Pathways	Targeted cells
Acetylcholine	Nicotinic acetylcholine receptors (nAChRs)	Mainly neurons and glial cells<br><br>Some expression on BBB endothelial cells
	Muscarinic acetylcholine receptors (mAChRs)	Neurons and glial cells
Methylphenidate	Dopamine Transporter (DAT)	Neurons
	Norepinephrine Transporter (NET)	
Cocaine	Dopamine Transporter (DAT)	Neurons
	Norepinephrine Transporter (NET)	
	Serotonin Transporter (SERT)	
Memantine	NMDA-type glutamate receptors	Neurons and some glial cells
Nicotine	Nicotinic acetylcholine receptors (nAChRs)	Mainly neurons and glial cells<br><br>Some expression on BBB endothelial cells
Glucose	GLUT1 (SLC2A1)	BBB endothelial cells
	GLUT3	Neurons
Tryptopane	LAT1	BBB endothelial cells
	Serotonin Pathway	Neurons and glial cells
Norepinephrine	Adrenergic receptors	Mainly neurons<br>Some glial and BBB endothelial cells

	Norepinephrine Transporter (NET)	Neurons

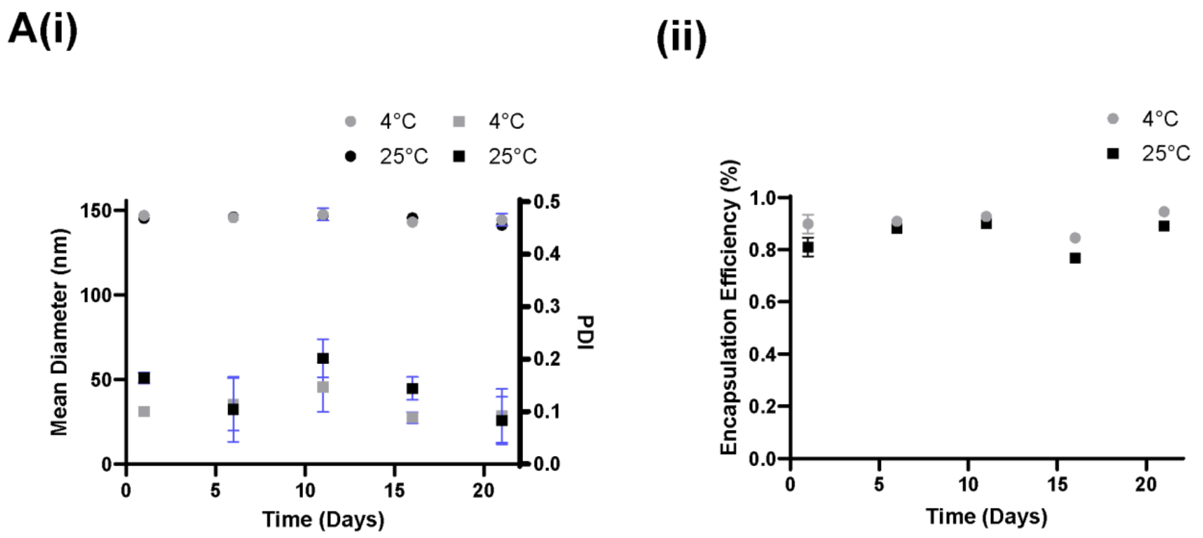

Figure S1. Stability of representative BT-LNPs. (A) Particles mean diameter (nm), PDI **(i)**, and mRNA encapsulation efficiency (%) **(ii)** of acetylcholine-LNPs measured over 20 days at both 4°C and 25°C storage temperatures. Data are presented as mean ± SD, n = 3 independent repetitions.

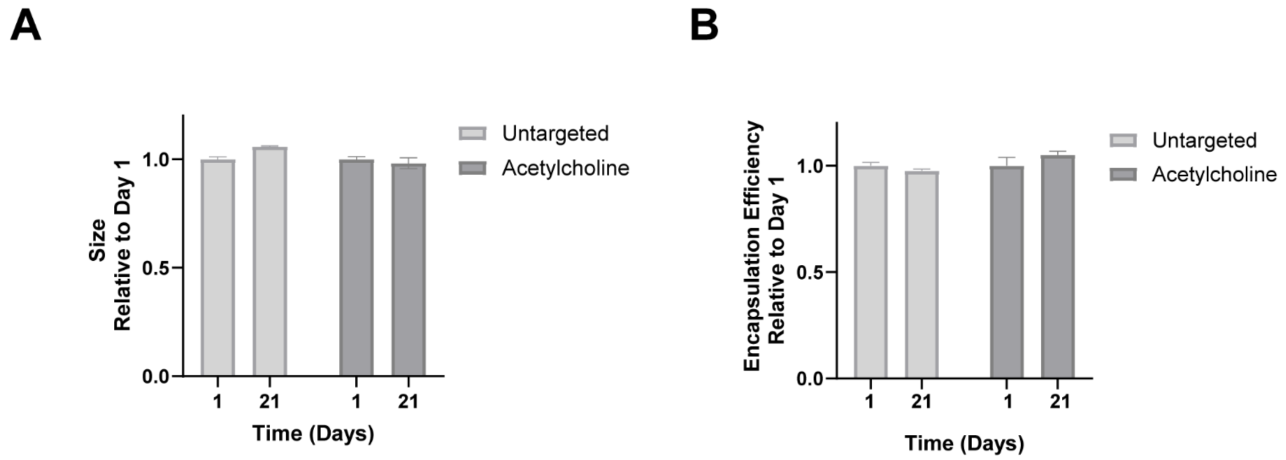

Figure S2. Stability of BT-LNPs and untargeted LNPs over 20 days. (A) Relative particle size and **(B)** encapsulation efficiency of representative BT-LNP formulation (acetylcholine) and untargeted LNPs, stored at 4°C and measured at days 1 and 21, normalized to respective day 0 values. Data represent mean±SD from three independent samples (n = 3).

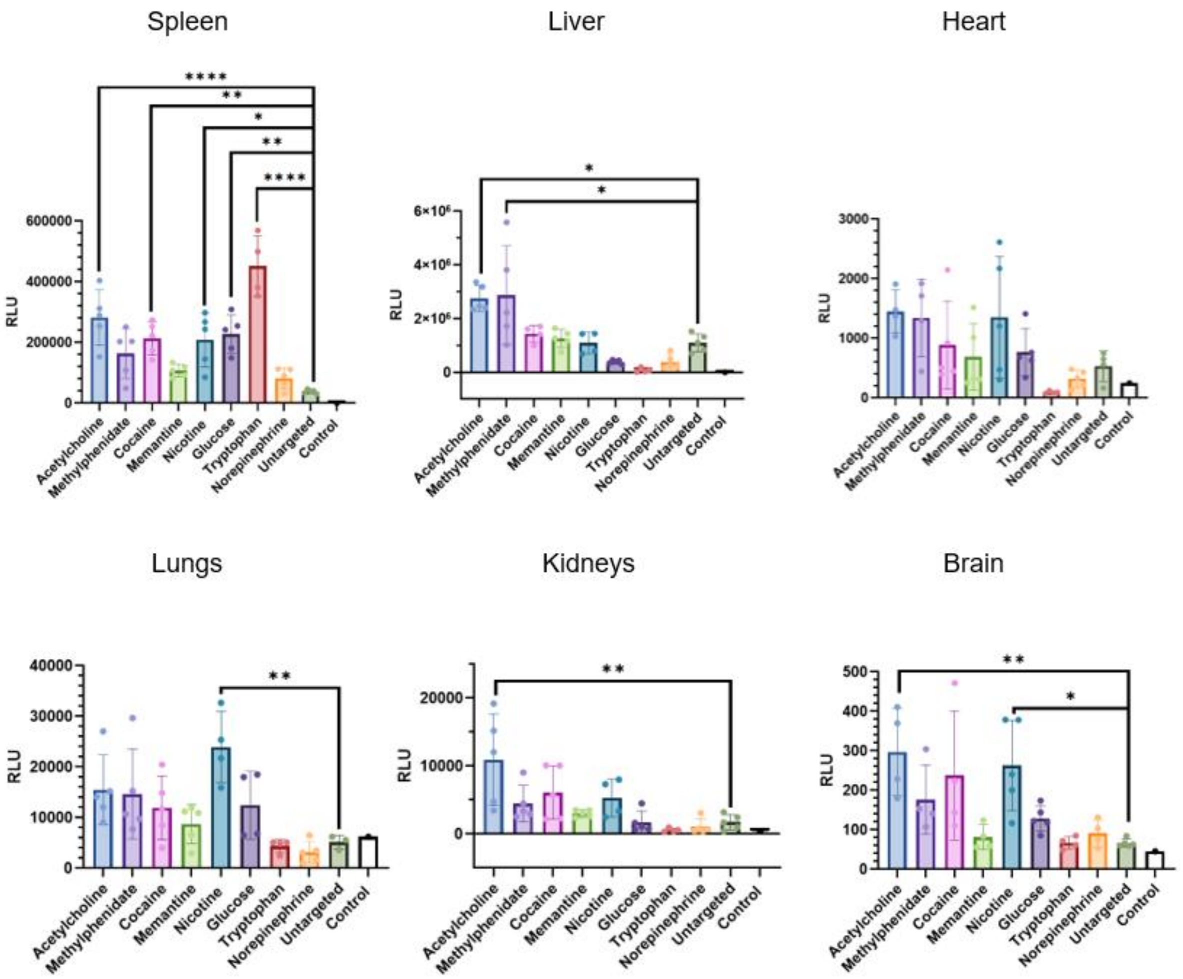

Figure S3. Analysis of luciferase mRNA expression in different organs of adult male C57BL/6 mice injected with BT-LNP library. Mice were injected intravenously with mRNA LNPs at a dose of 0.729 mg/kg, perfused with PBS, and sacrificed after 6 h; non-injected mice were used as a control group. The luciferase signal's Relative Luminescence Unit (RLU) was measured using a microplate reader and reported as mean ± SD (n = 5). One-way ANOVA with multiple comparisons test was used to determine statistical significance

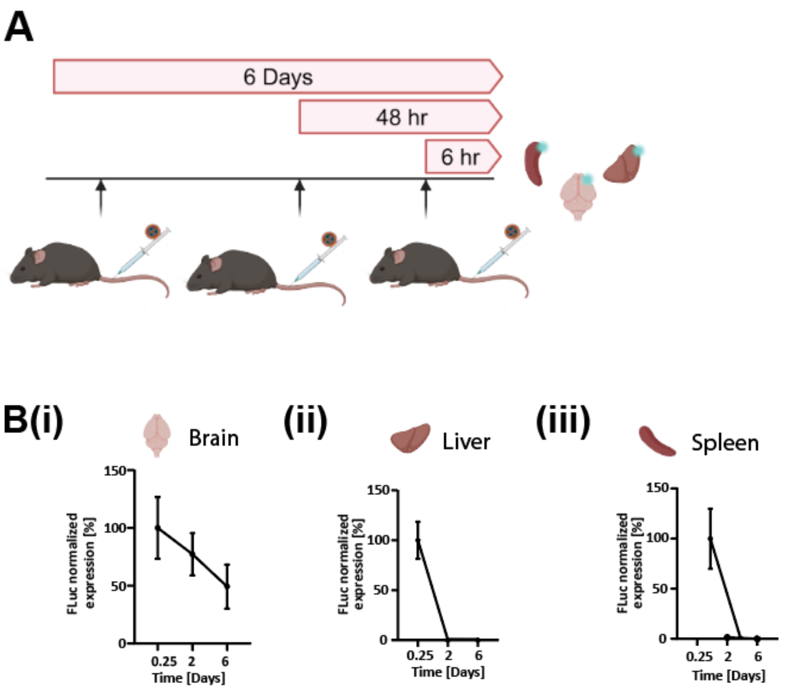

Figure S4. Temporal dynamics of mRNA expression following systemic administration of BT-LNPs. (A) Temporal dynamics of mRNA in vivo expression in brain, liver, and spleen following IV administration of FLuc-LNPs over six days. Glucose-LNPs served as a reference formulation. (B) Firefly luciferase (FLuc) expression was quantified over time in **(i)** brain, **(ii)** liver, and **(iii)** spleen. mRNA expression was normalized to peak levels and plotted as a percentage over 0.25, 2-, and 6-days post-injection. Data is normalized to the 0.25-day value for each organ and presented as mean ± SD (n=5 mice per group).

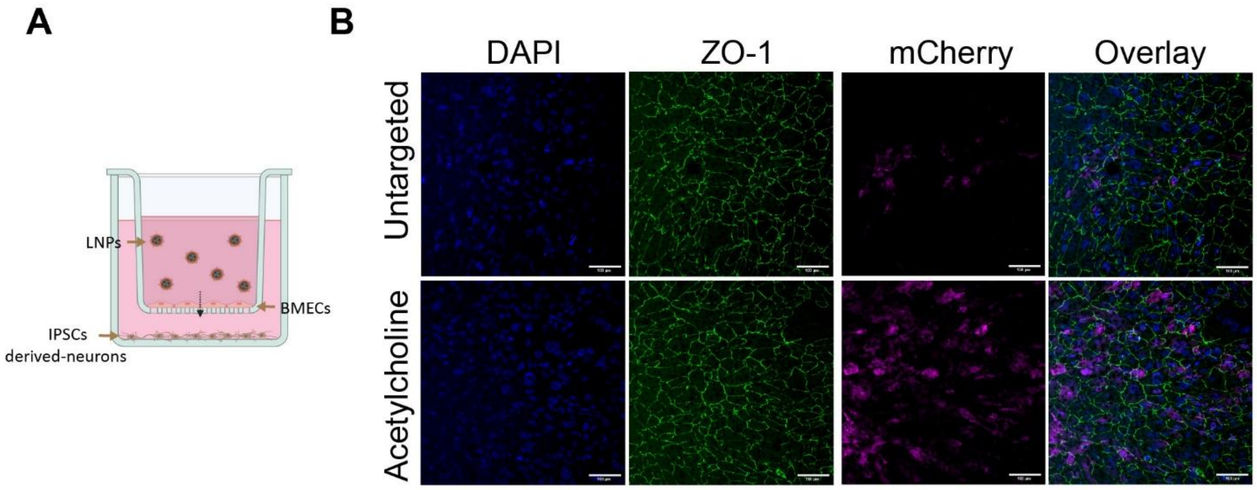

Figure S5. Transfection of human brain microvascular endothelial cells (BMECs) in an in vitro BBB model. (A) Schematic of the transwell-based BBB model with BMECs seeded apically and iPSC-derived neurons basolaterally. Acetylcholine -LNPs or untargeted LNPs carrying mCherry mRNA were added to the apical side. **(B)** Confocal images of the BMEC layer 24 h post-treatment. ZO-1 (green) marks tight junctions, DAPI (blue) stains nuclei, and mCherry (magenta) indicates transfection; n=3 biological repetitions; Scale bars: 100 µm.

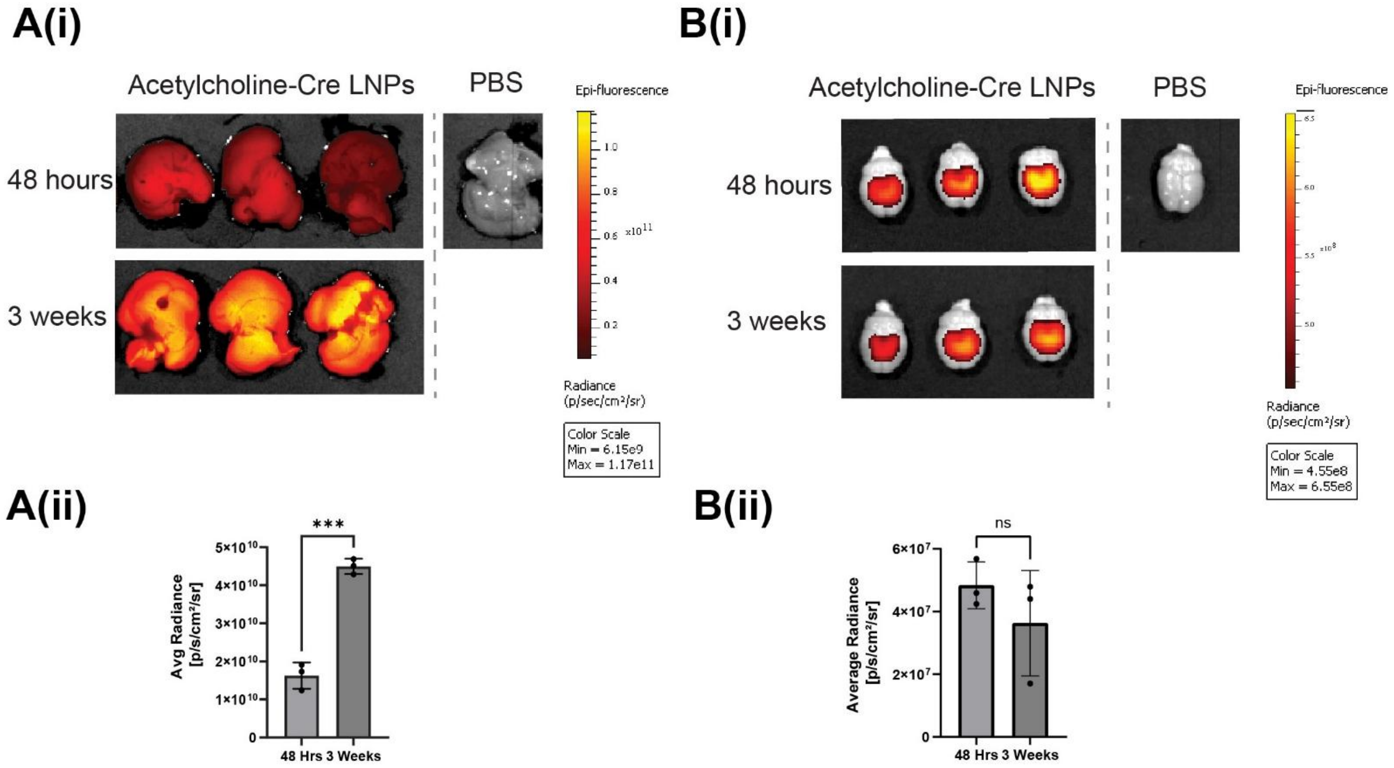

Figure S6. Temporal analysis of tdTomato expression in Ai9 mice following systemic delivery of BT-LNPs. Acetylcholine-conjugated LNPs encapsulating Cre mRNA were intravenously administered to Ai9 mice at 0.729 mg/kg. Liver and brain tissues were extracted 48 hours or 3 weeks post-injection (n=3 mice per group). **(A)** *In vivo* imaging system (IVIS) images **(i)** and quantification **(ii)** of liver tdTomato fluorescence at both time points. **(B)** IVIS images **(i)** and quantification **(ii)** of brain tdTomato fluorescence at both time points. Data are mean ± SD, unpaired t-test with multiple comparisons adjustment. ***p = 0.0002, ns=non-significant (p = 0.6387).

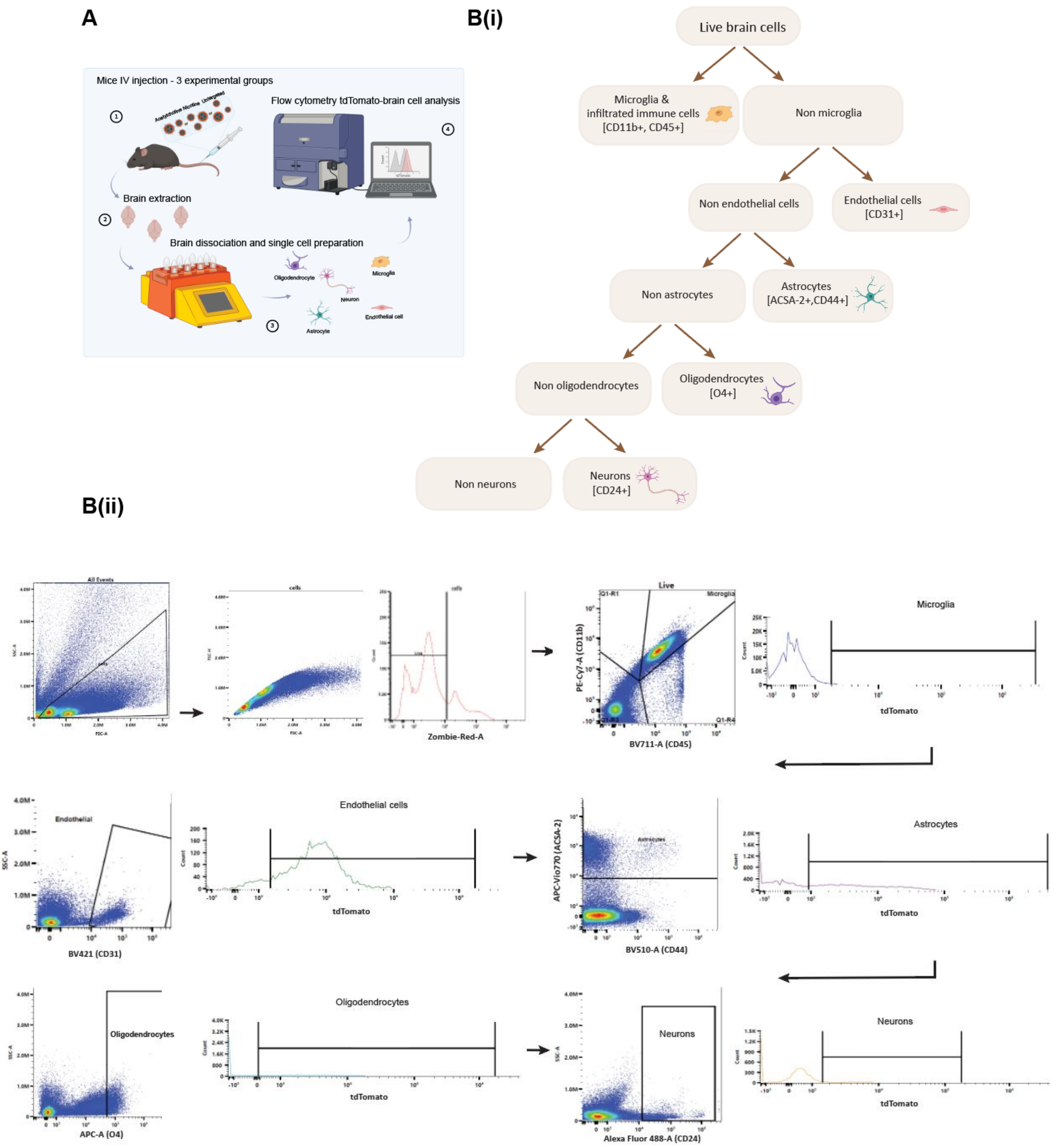

Figure S7. Gating strategy for brain cells flow cytometry analysis. (A) Mice were intravenously injected with selected BT-LNPs (e.g., Acetylcholine, Nicotine, and untargeted) encapsulating Cre-mRNA. 48 hours post-injection, brains were extracted and dissociated into single-cell suspensions. Flow cytometry analysis was used to assess tdTomato expression in various brain cells. **(B) (i-ii)** The gating strategy used to analyze tdTomato expression in different brain cell populations. The total cell population is first identified by plotting

side scatter area (SSC-A) against forward scatter area (FSC-A), excluding debris. The main cell population is gated and labeled as "cells", followed by plotting forward scatter height (FSC-H) against forward scatter area (FSC-A), and live cells are selected by gating on Zombie Red-negative events. Microglia and potential infiltrating immune cells are excluded by gating on CD11b (PE-Cy7)-positive and CD45 (BV711)-positive events, with tdTomato-positive microglia identified separately. Endothelial cells are removed by gating on CD31 (BV421)-positive events, followed by identifying tdTomato-positive endothelial cells. Astrocytes are excluded by gating on ACSA-2 (APC-Vio770)-positive and CD44 (BV510)-positive events, and tdTomato-positive astrocytes are identified. Oligodendrocytes are eliminated by gating on O4 (APC)-positive events, with tdTomato-positive oligodendrocytes detected. Finally, neurons are excluded by gating on CD24 (Alexa-Fluor 488)-positive events, and tdTomato-positive neurons are identified. This sequential gating strategy effectively isolates brain cell types and enables detection of tdTomato-positive populations within each subset. The representative example presents an analysis of an acetylcholine-LNP-treated brain sample. Illustrations created with Biorender.com.

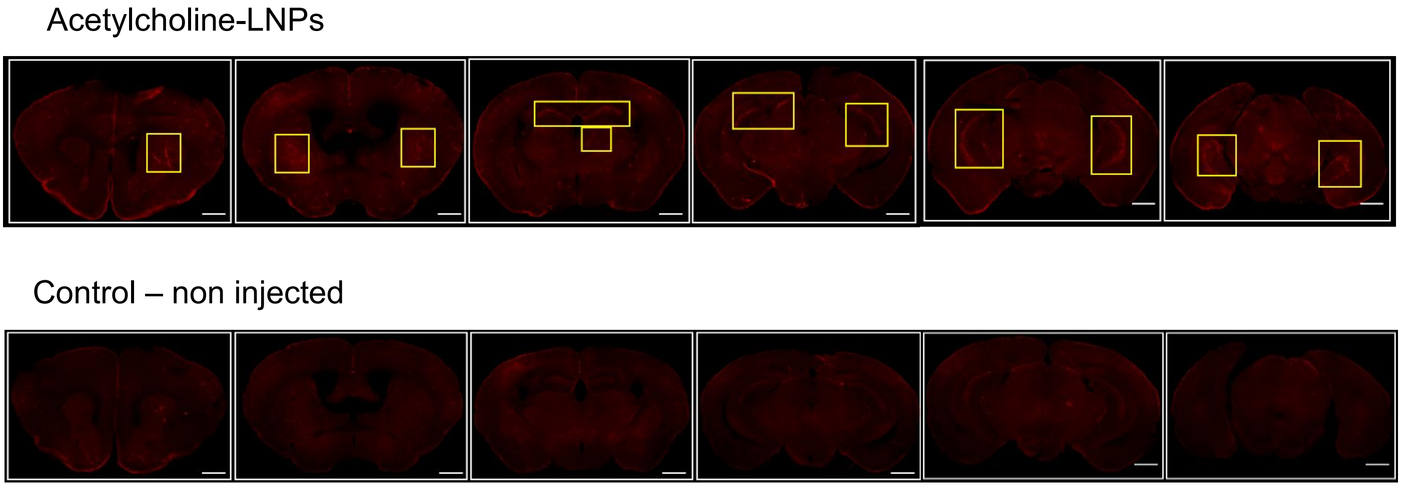

Figure S8. Acetylcholine-LNPs enabled brain tdTomato activation by Cre mRNA delivery *via* intravenous administration. Imaging microscopy of representative brain sections was employed to verify effective tissue tdTomato expression. The expression areas are labeled by yellow rectangles. Scale bar: 1000 µm.

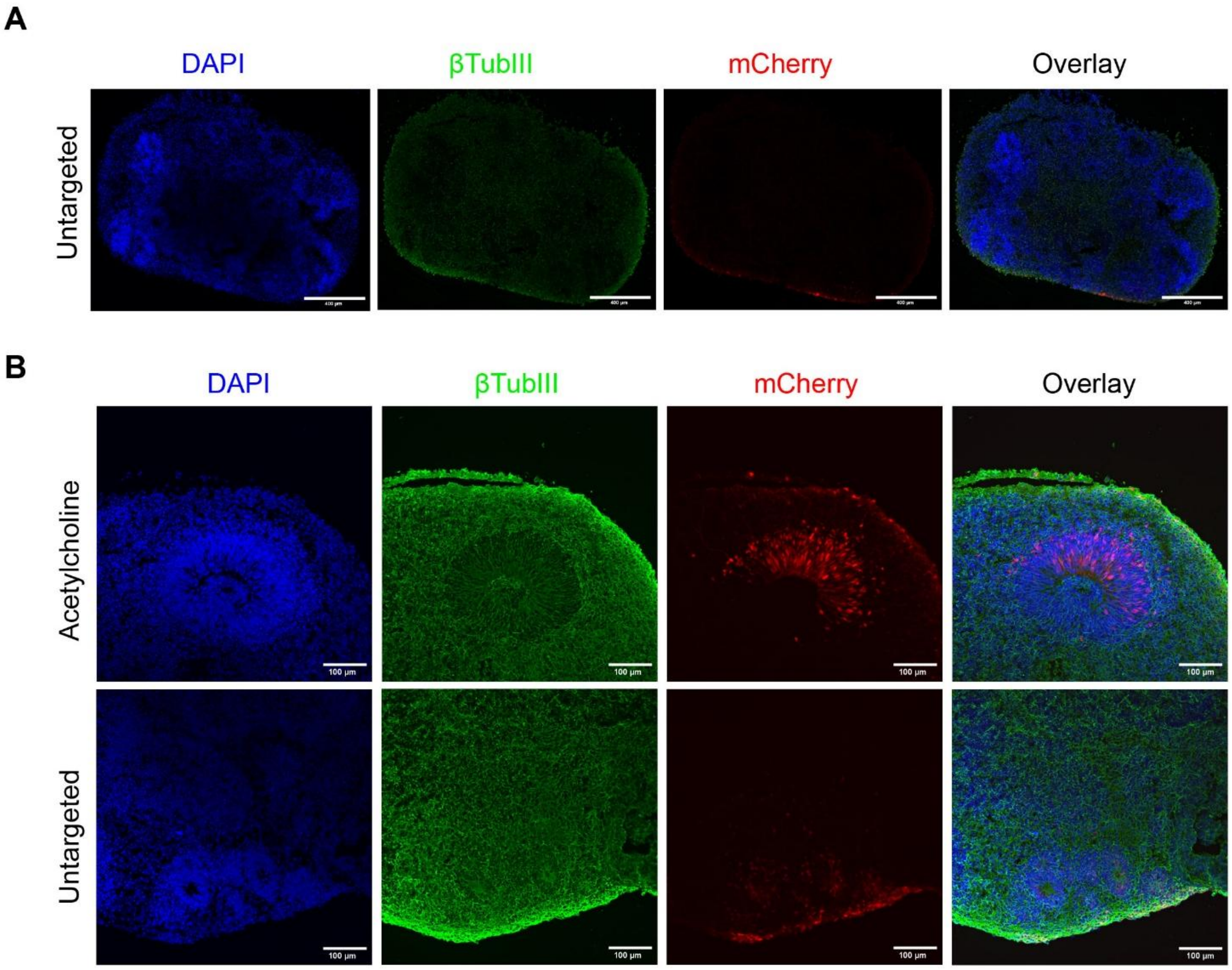

Figure S9. Transfection in deep layers and rosette-like structures in human cortical organoids following acetylcholine-LNP treatment. (A) Representative immunofluorescence images of whole brain organoid sections treated with untargeted LNPs, showing DAPI (blue), β-Tubulin III (green), and mCherry (red). Scale bar: = 400 µm. **(B)** Representative higher magnification (20×) of cortical-like regions highlights robust mCherry expression localized to rosette-like neuroepithelial structures in acetylcholine-LNP-treated organoids, with a lower expression level in untargeted controls. Scale bar: 100 µm. n=2 independent repetitions.

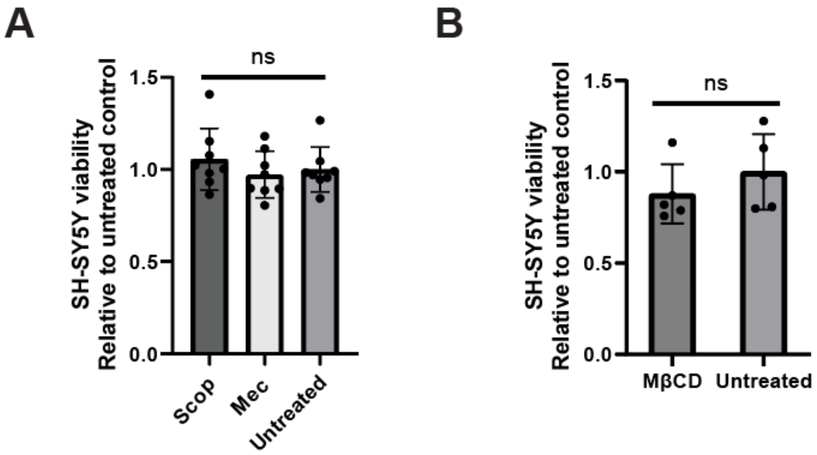

Figure S10. Evaluation of SH-SY5Y cell viability following inhibitor treatments. Viability assays were conducted to assess the cytotoxicity of the inhibitors in uptake mechanism experiments. **(A)** SH-SY5Y cells were treated with 2 mM Scopolamine (Scop) or 10 µM Mecamylamine (Mec). **(B)** Cells were treated with 4.5 mM Methyl-β-cyclodextrin (MβCD). Viability was measured relative to untreated controls. No significant cytotoxicity was observed in any group. Data is presented as mean ± SD, n = 5–8 independent samples; ns = not significant.

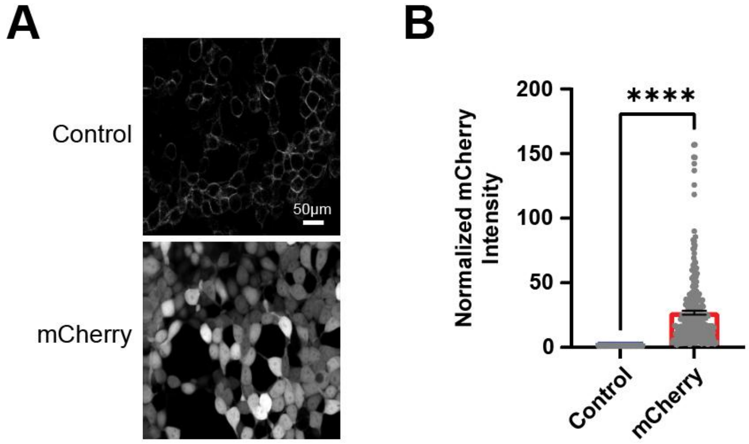

Figure S11. Assessment of mRNA transfection in HEK293 cells following acetylcholine-LNP treatment. (A), (B) Representative images and quantification of normalized mCherry fluorescent intensity in HEK293 transfected with the AchLightG sensor without (control) or with overnight application of mCherry mRNA-encapsulating acetylcholine-LNPs (1.02 ± 0.006, n = 200 or 26.86 ± 1.585, n = 300), respectively. Scale bar: 50 µm. 'n' denotes the number of cells, error bars represent S.E.M. Statistical differences were measured using an unpaired two-tailed Student T-test. Representative images represent experiments that were repeated independently at least three times. **** $p < 0.0001$.

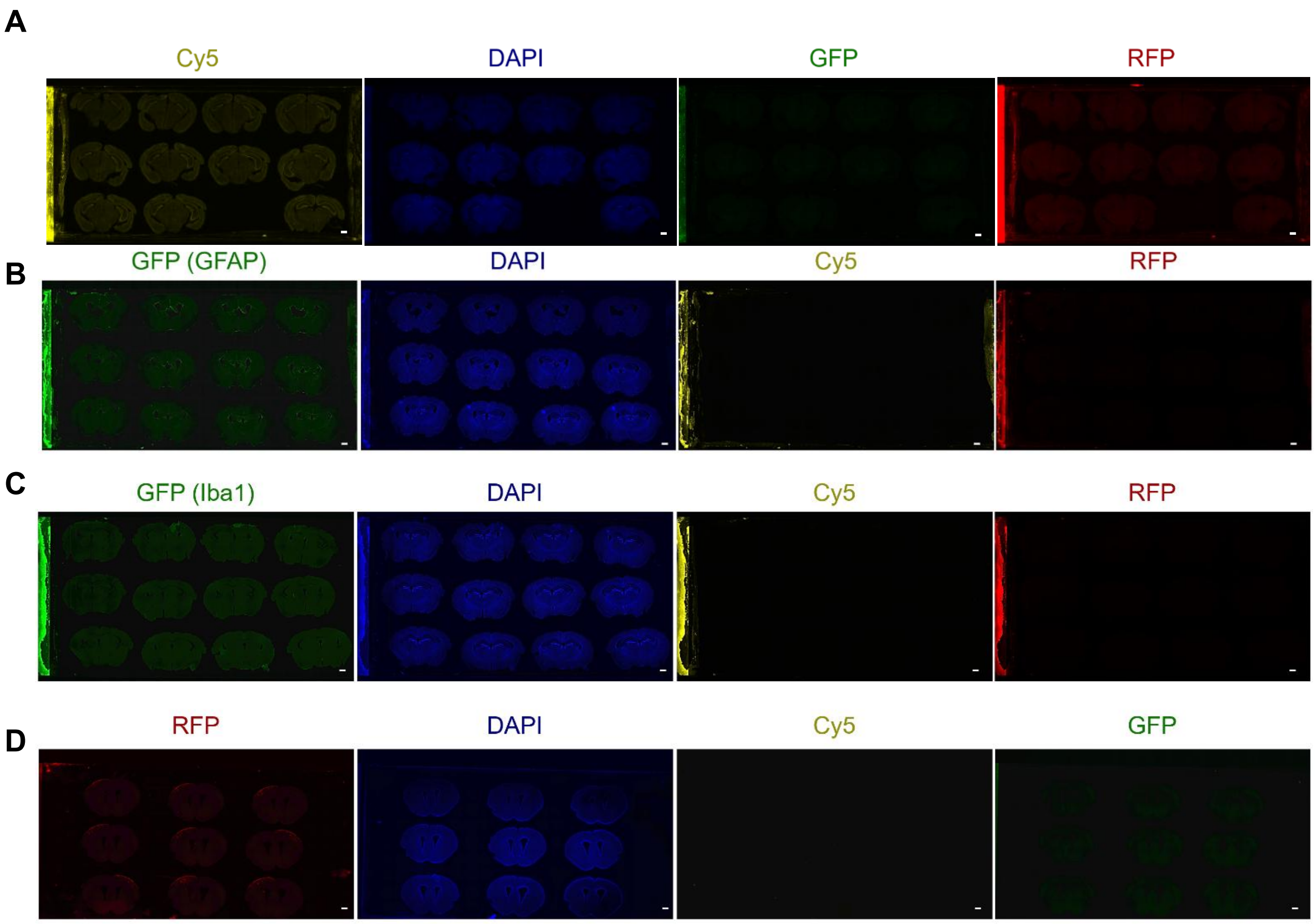

Figure S12. Controls for dye channels in the immunostaining of brain sections. (A) Representative brain sections showing the Cy5 channel (yellow) alongside DAPI (blue), with no staining observed in the GFP (green) and RFP (red) channels, verifying the absence of crosstalk between channels. Scale bar: 1000 µm. **(B)** Representative brain sections showing the GFP channel for GFAP (green) alongside DAPI (blue), with no staining observed in the Cy5 (yellow) and RFP (red) channels, verifying the absence of crosstalk between channels. Scale bar: 1000 µm. **(C)** Representative brain sections showing the GFP channel for Iba1 (green) alongside DAPI (blue), with no staining observed in the Cy5 (yellow) and RFP (red) channels, verifying the absence of crosstalk between channels. Scale bar: 1000 µm. **(D)** Representative brain sections showing the RFP channel (red) alongside DAPI (blue), with no staining observed in the Cy5 (yellow) and GFP (green) channels, verifying the absence of crosstalk between channels. Scale bar: 1000 µm.

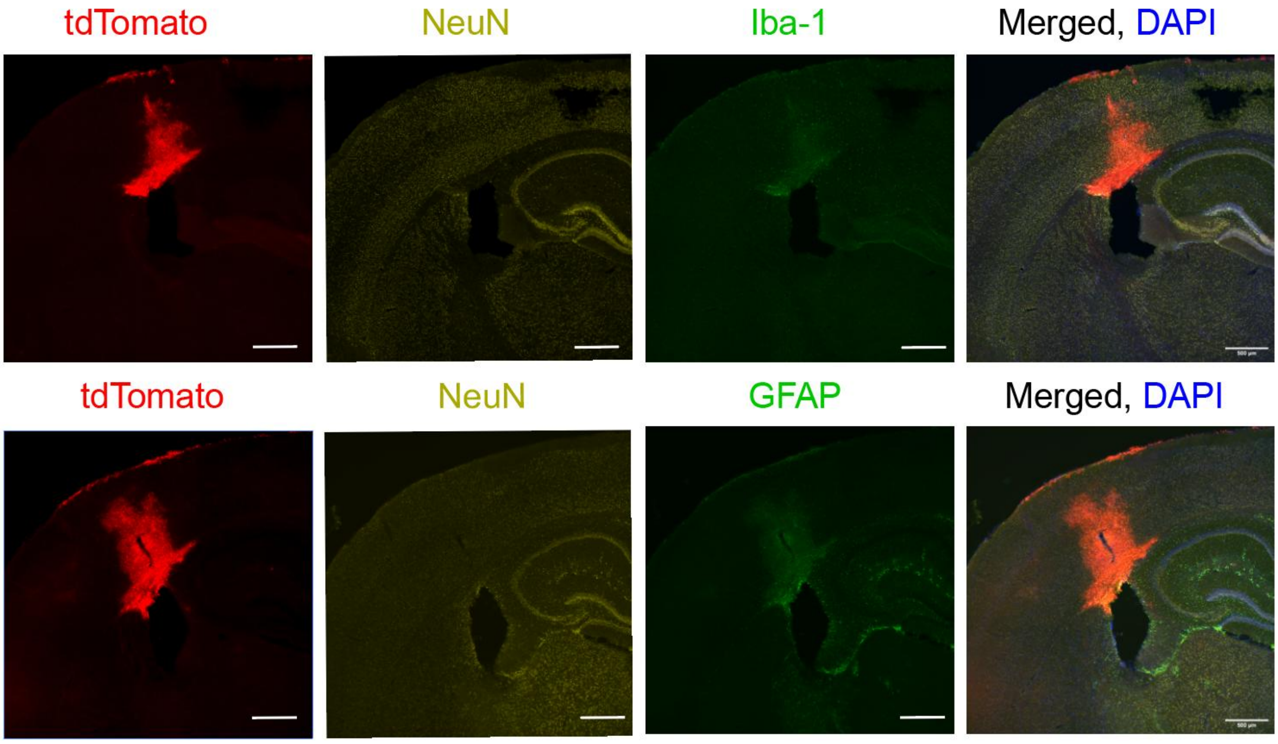

Figure S13. Untargeted-LNPs enabled tdTomato activation by Cre mRNA delivery via intracerebral administration in the cortical brain area. **(A)** Representative images of brain sections showing co-localization of tdTomato (red) with neuronal marker NeuN (yellow) and microglial marker Iba1 (green). The merged image also includes DAPI (blue) staining for nuclear visualization. Scale bar: 500 µm. **(B)** Representative images of brain sections showing co-localization of tdTomato (red) with neuronal marker NeuN (yellow) and astrocytic marker GFAP (green). The merged image also includes DAPI (blue) staining for nuclear visualization. Scale bar: 500 µm.

A

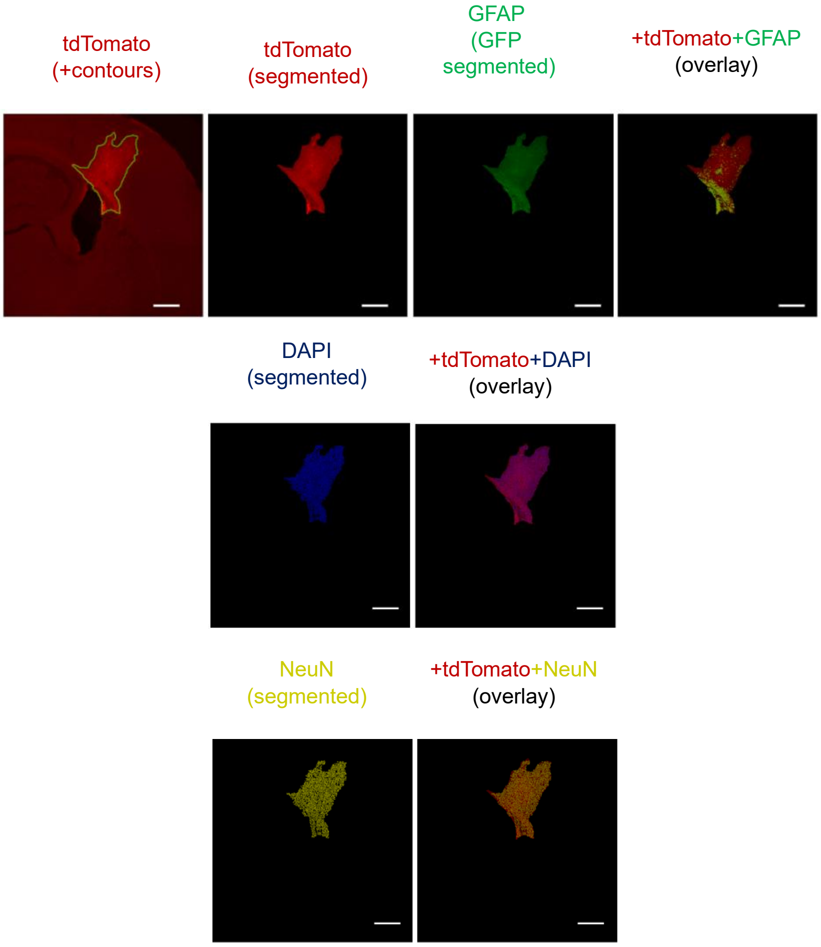
tdTomato (+contours)
tdTomato (segmented)
GFAP (GFP segmented)
+tdTomato+GFAP (overlay)
DAPI (segmented)
+tdTomato+DAPI (overlay)
NeuN (segmented)
+tdTomato+NeuN (overlay)

B

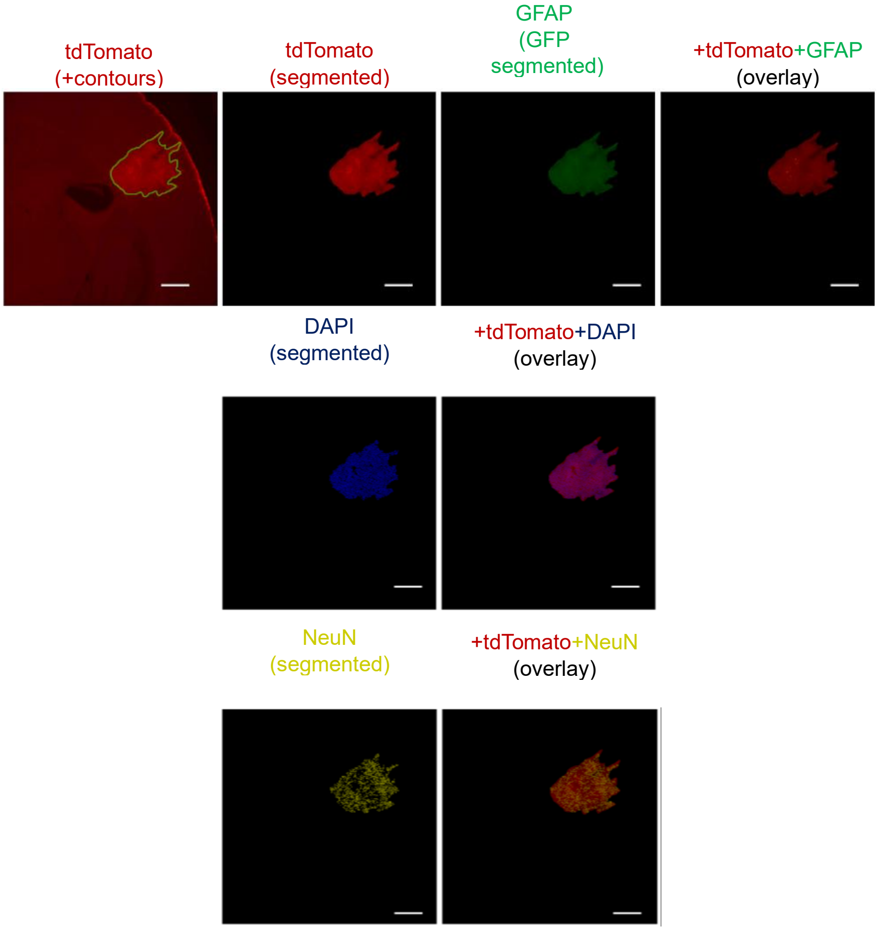
tdTomato (+contours)
tdTomato (segmented)
GFAP (GFP segmented)
+tdTomato+GFAP (overlay)
DAPI (segmented)
+tdTomato+DAPI (overlay)
NeuN (segmented)
+tdTomato+NeuN (overlay)

C

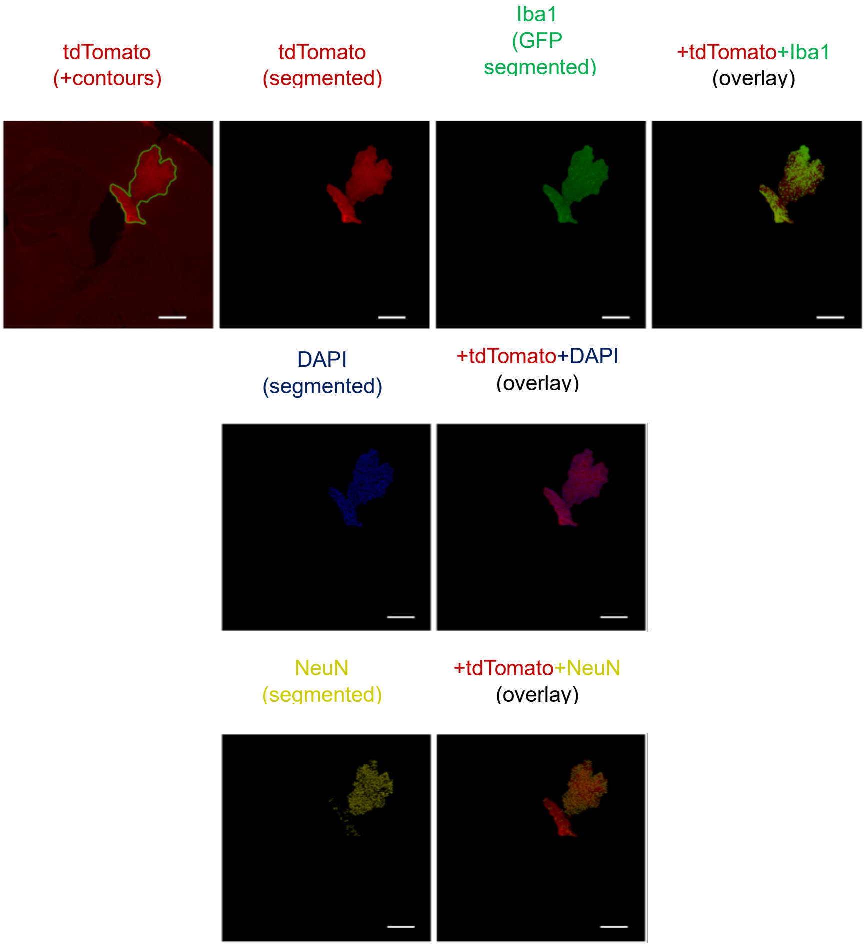
tdTomato
(+contours)
tdTomato
(segmented)
Iba1
(GFP
segmented)
+tdTomato+Iba1
(overlay)
DAPI
(segmented)
+tdTomato+DAPI
(overlay)
NeuN
(segmented)
+tdTomato+NeuN
(overlay)

D

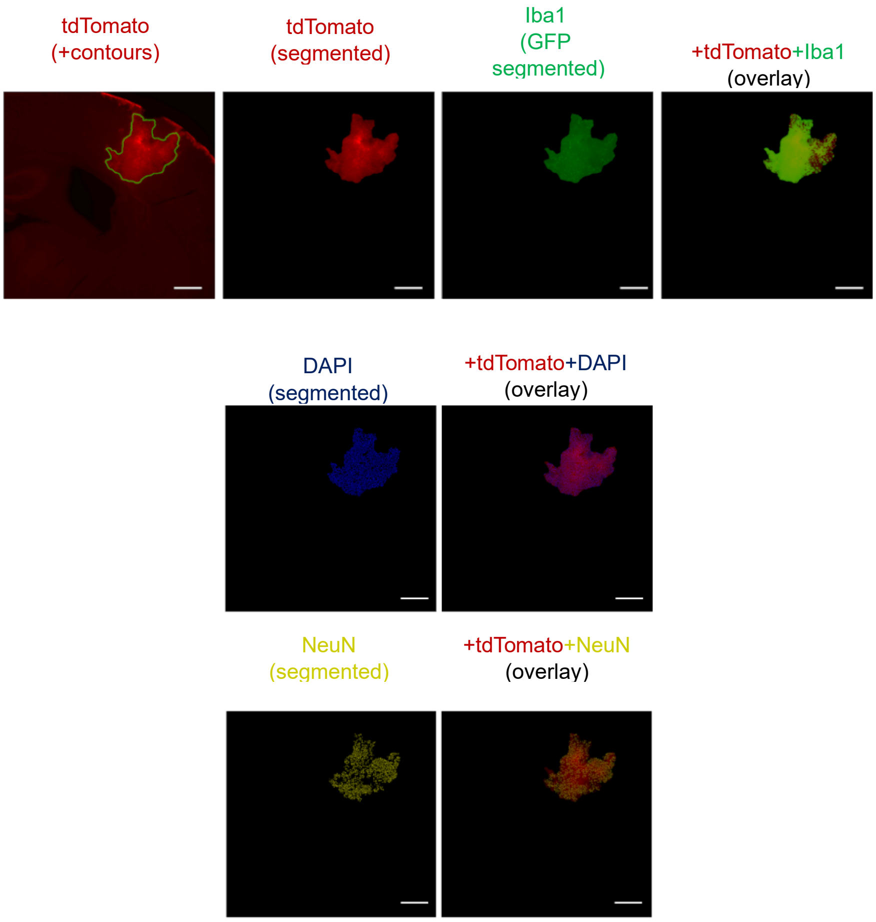
tdTomato
(+contours)
tdTomato
(segmented)
Iba1
(GFP
segmented)
+tdTomato+Iba1
(overlay)
DAPI
(segmented)
+tdTomato+DAPI
(overlay)
NeuN
(segmented)
+tdTomato+NeuN
(overlay)

Figure S14. Segmentation of LNPs-mediated tdTomato activation following intracerebral administration Cre mRNA delivery. (A, B) Brain sections from the (A) acetylcholine-LNPs and (B) untargeted-LNPs groups, respectively, demonstrating tdTomato (red), GFAP (green), DAPI (blue), and NeuN (yellow), with segmentation and overlays. **(C, D)** Representative sections from the (C) Acetylcholine-LNPs and (D) untargeted-LNPs groups, respectively, showing tdTomato (red), Iba1 (green), DAPI (blue), and NeuN (yellow) with segmentation and overlays. For each panel, tdTomato (+contours) shows RFP-positive (tdTomato) regions in red with segmentation boundaries, while tdTomato (segmented) displays the same regions without segmentation contours. GFAP or Iba1 (GFP segmented) highlights astrocytes (GFAP) or microglia (Iba1) in the green channel. The +tdTomato+GFAP or +tdTomato+Iba1 overlay images present a merged view of tdTomato (red) and GFAP or Iba1 (green), with yellow indicating colocalization. DAPI (segmented) shows the blue channel segmentation of DAPI-stained nuclei, and +tdTomato+DAPI (overlay) displays merged tdTomato (red) and DAPI (blue) signals, with colocalization appearing purple. NeuN (segmented) represents NeuN-positive neurons in the Cy5 channel (yellow), while +tdTomato+NeuN (overlay) shows merged tdTomato (red) and NeuN (yellow) channels, with colocalization appearing orange. Scale bar: 500 µm.

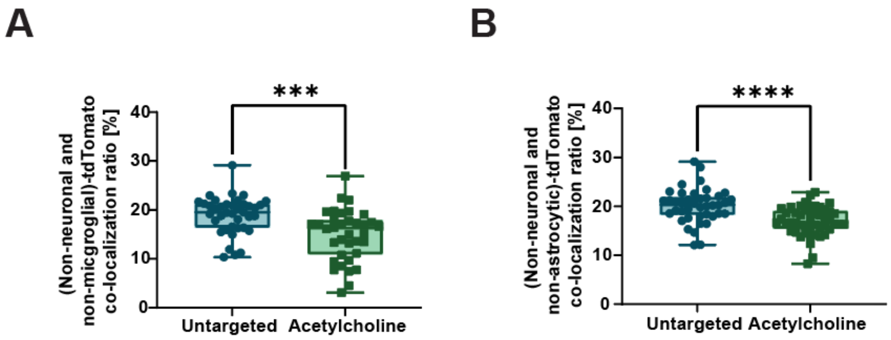

Figure S15. Image analysis of cellular specificity of BT-LNPs following intracerebral injection. (A) Box dot plot graph of brain cells (non-neuronal and non-microglia cells)-tdTomato co-localization ratio (%). **(B)** Box dot plot graph of brain cells (non-neuronal and non-astrocyte cells)-tdTomato co-localization ratio (%). Data is expressed as a mean ± SD (n=4 independent repetitions performed with at least 20 replicates each); Unpaired t-test p-value; *** p=0.0002; **** p <0.0001.

Supplementary movies

Movie S1. LNP_Ach_AchLightG. A movie showing changes in AchLightG fluorescent intensity following acetylcholine-targeted LNP application in HEK293. Images were taken every 2 minutes continuously over 34 minutes. Frame dimensions are 487.5 µm x 487.5 µm.

Movie S2. LNP_Untargeted_AchLightG. A movie shows changes in AchLightG fluorescent intensity following untargeted LNP application in HEK293. Images were taken every 2 minutes continuously over 34 minutes. Frame dimensions are 487.5 µm x 487.5 µm.

Movie S3. LNP_Ach_AchLightG-mCherry_merged. A movie showing changes in AchlightG (green) and mCherry (fire scale) fluorescent intensity following mCherry mRNA-encapsulating acetylcholine-targeted LNPs application in HEK293. Images were taken every 30 minutes continuously over 23 hours. Frame dimensions are 487.5 µm x 487.5 µm.
