# Supplementary material for: AI-Validated Brain Targeted mRNA Lipid Nanoparticles with Neuronal Tropism
Source: ACS Nano. 2025 Sep 16;19(41):36106–28. doi: 10.1021/acsnano.4c15013 (PMC12548354; doi:10.1021/acsnano.4c15013)
Supplement: Supplementary file 5 [file nn4c15013_si_005.pdf]

Compound spectra

DMG-PEG2000-NH2

H-NMR spectrum of sample; solvent: CDCl3

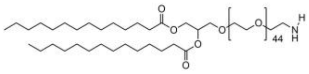

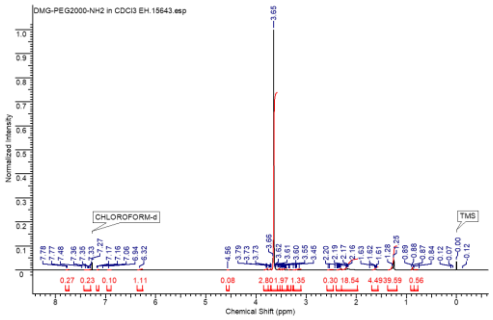

DMG-PEG2000-NH2

Zoom -H-NMR spectrum of sample; solvent: CDCl3

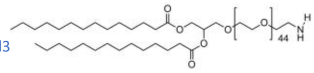

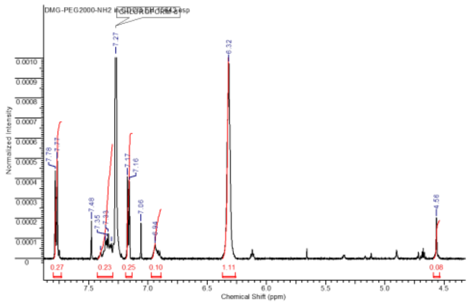

DMG-PEG2000-NH2

Zoom -H-NMR spectrum of sample; solvent: CDCl3

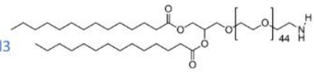

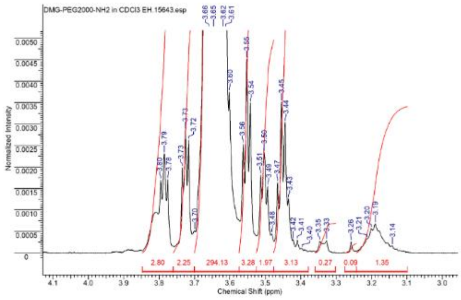

DMG-PEG2000-NH2

Zoom -H-NMR spectrum of sample; solvent: CDCl3

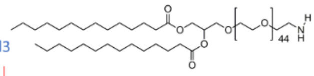

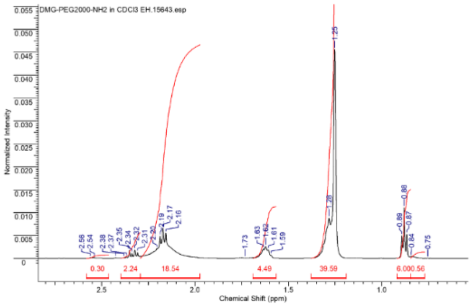

DMG-PEG2000-NH2

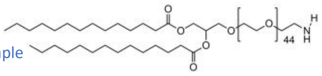

Proposed 1H-NMR assignment of main peaks of sample

^{1}HNMR Chemical shifts (ppm)	Functional group	Structure
0.88	CH_3-	Aliphatic CH_3<br>$\underline{CH_3}$-$(CH_2)_n$
1.25	-$(CH_2)_n$	Aliphatic -CH_2-<br>CH_3-$\underline{(CH_2)_n}$
1.62	-CH_2-	-$\underline{CH_2}$-CH_2-COO
**2.19-2.34**	**-CH_2-**	CH_2-bonded to amine group<br>**-$\underline{CH_2}$-NH_2**
3.19-3.79	-CH_2-	-$\underline{CH_2}$-O-
6.32	-CH-	Probably -$\underline{CH}$-O-

S1. ^{1}H NMR of DMG-PEG2000-NH2

DMG-PEG2000-Tryptophan

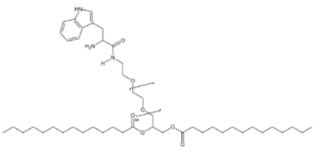

H-NMR spectrum of sample; solvent: DMSO-d6

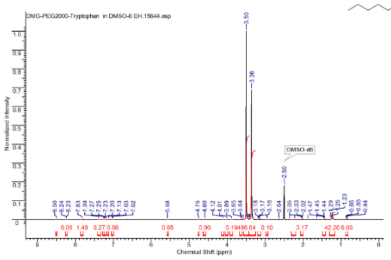

DMG-PEG2000-Tryptophan

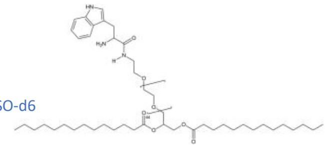

Zoom - H-NMR spectrum of sample; solvent: DMSO-d6

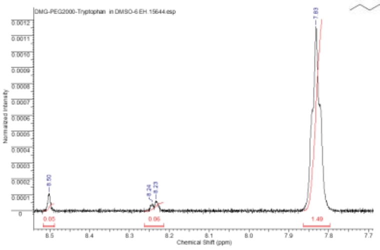

DMG-PEG2000-Tryptophan

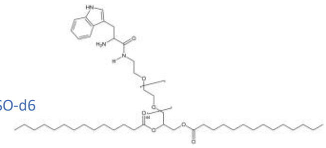

Zoom - H-NMR spectrum of sample; solvent: DMSO-d6

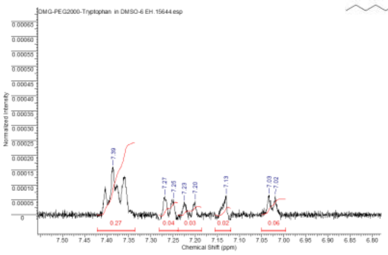

DMG-PEG2000-Tryptophan

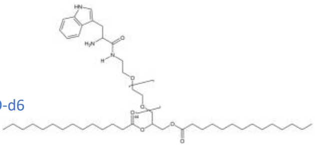

Zoom - H-NMR spectrum of sample; solvent: DMSO-d6

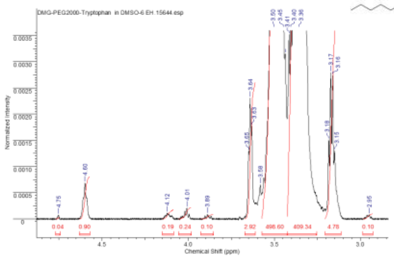

DMG-PEG2000-Tryptophan

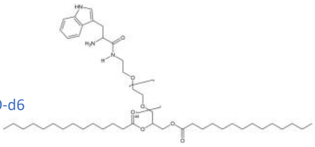

Zoom - H-NMR spectrum of sample; solvent: DMSO-d6

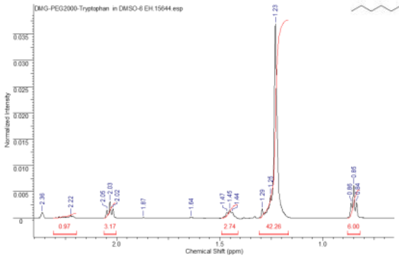

DMG-PEG2000-Tryptophan

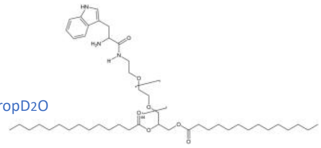

H-NMR spectrum of sample; solvent: DMSO-d6+dropD2O

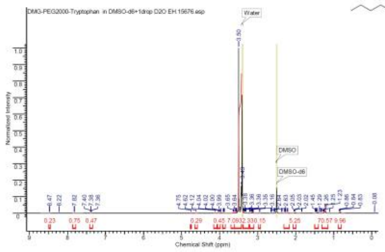

DMG-PEG2000-Tryptophan

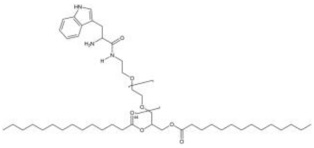

Zoom - H-NMR spectrum of sample; solvent: DMSO-d6+dropD2O

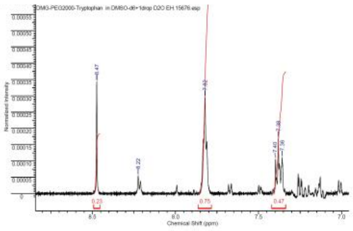

DMG-PEG2000-Tryptophan

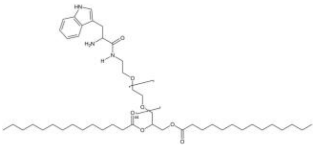

Zoom - H-NMR spectrum of sample; solvent: DMSO-d6+dropD2O

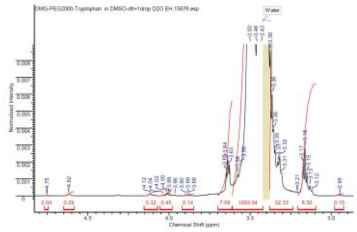

DMG-PEG2000-Tryptophan

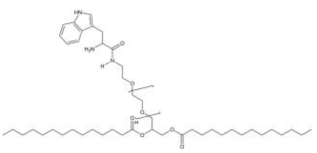

Zoom - H-NMR spectrum of sample; solvent: DMSO-d6+dropD2O

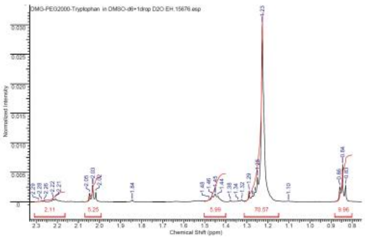

DMG-PEG2000-Tryptophan

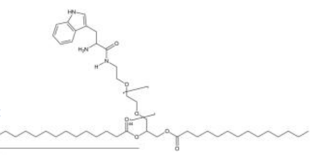

Comparison of H-NMR spectrum of sample; solvent: DMSO-d6+dropD2O vs. DMSO-d6

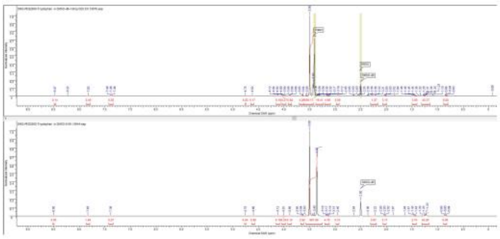

DMG-PEG2000-Tryptophan

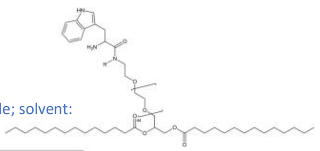

Zoom of Comparison of H-NMR spectrum of sample; solvent: DMSO-d6+dropD2O vs. DMSO-d6

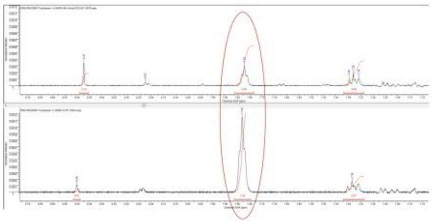

DMG-PEG2000-Tryptophan

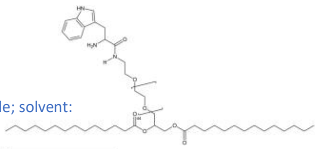

Zoom of Comparison of H-NMR spectrum of sample; solvent: DMSO-d6+dropD2O vs. DMSO-d6

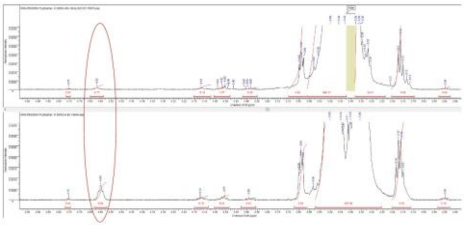

DMG-PEG2000-Tryptophan

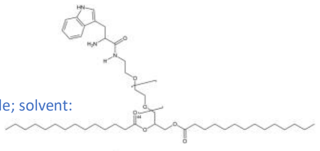

Zoom of Comparison of H-NMR spectrum of sample; solvent: DMSO-d6+dropD2O vs. DMSO-d6

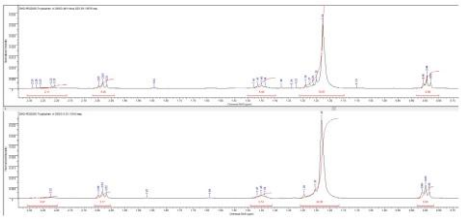

DMG-PEG2000-Tryptophan

Proposed 1H-NMR assignment of main peaks of sample:

^{1}HNMR Chemical shifts (ppm)	Functional group	Structure
0.85	CH_3-	Aliphatic CH_3 CH_3-$(CH_2)_n$
1.23	-$(CH_2)_n$	Aliphatic -CH_2- CH_3-$(CH_2)_n$
1.45	-CH_2-	-CH_2-CH_2-COO
2.03-2.22	-CH_2-	CH_2-bonded to: -CH_2-COO/-CH_2-NH
3.36		Water (belongs to solvent)
3.17-4.60	-CH_2-/-CH-	-CH_2-O-/-CH-NH_2-/-CH_2-CH-NH_2/-CH_2-NH
7.00-8.50	-CH-	Aromatic ring

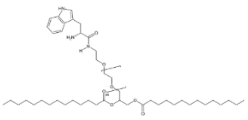

^{1}HNMR Chemical shifts (ppm)	Functional group	Structure
7.80-7.85	NH	-NH- in aromatic ring
4.6	-NH-	-CH_2-NH-

S2. ^{1}H NMR of DMG-PEG2000-NH2-Tryptophan

DMG-PEG2000-Glucose

H-NMR spectrum of sample; solvent: DMSO-d6

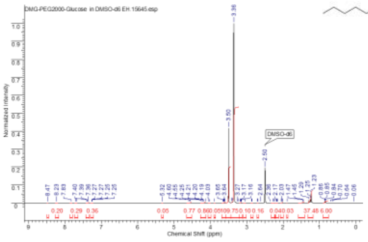

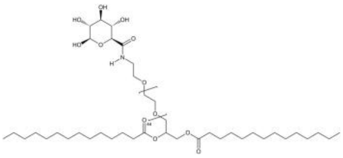

DMG-PEG2000-Glucose

Zoom - H-NMR spectrum of sample; solvent: DMSO-d6

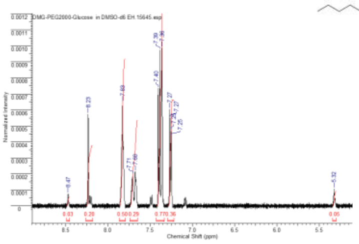

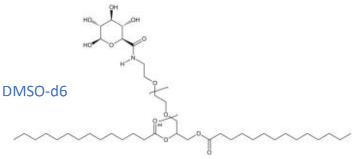

DMG-PEG2000-Glucose

Zoom - H-NMR spectrum of sample; solvent: DMSO-d6

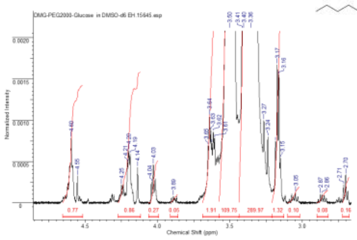

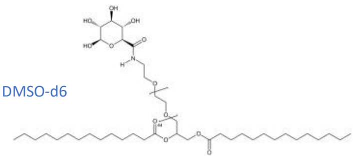

DMG-PEG2000-Glucose

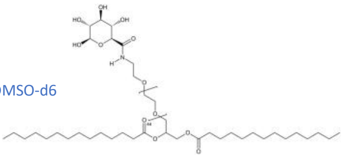

Zoom - H-NMR spectrum of sample; solvent: DMSO-d6

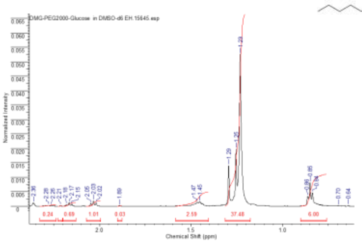

DMG-PEG2000-Glucose

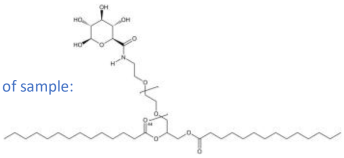

Proposed 1H-NMR assignment of main peaks of sample:

^{1}HNMR Chemical shifts (ppm)	Functional group	Structure
0.85	CH_3-	Aliphatic CH_3 $\underline{CH_3}$-$(CH_2)_n$
1.23	-$(CH_2)_n$	Aliphatic -CH_2- CH_3-$\underline{(CH_2)_n}$
1.45	-CH_2-	-$\underline{CH_2}$-CH_2-COO
2.03-2.17	-CH_2-	CH_2-bonded to -$\underline{CH_2}$-COO/-$\underline{CH_2}$-NH
**3.17-4.60**	**-CH_2-/-CH-**	**-$\underline{CH_2}$-O-/-CH-OH**

S3. ^{1}H NMR of DMG-PEG2000-NH2-Glucose

DMG-PEG2000-Norepinephrine

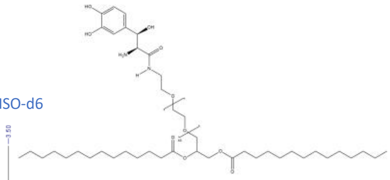

H-NMR spectrum of sample; solvent: DMSO-d6

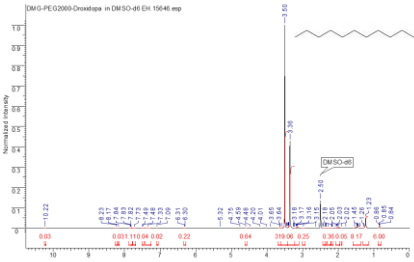

DMG-PEG2000-Norepinephrine

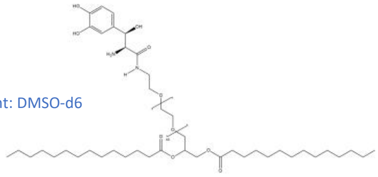

Zoom - H-NMR spectrum of sample; solvent: DMSO-d6

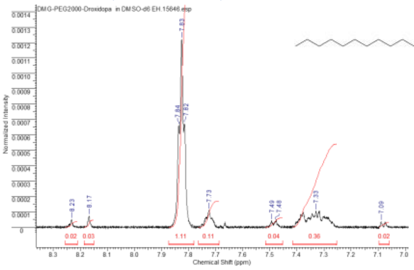

DMG-PEG2000-Norepinephrine

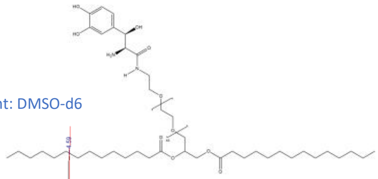

Zoom - H-NMR spectrum of sample; solvent: DMSO-d6

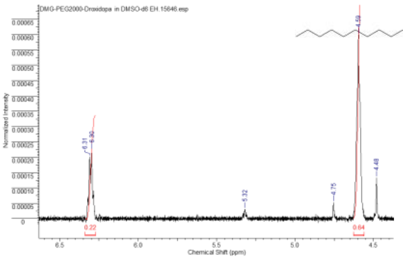

DMG-PEG2000-Norepinephrine

Zoom - H-NMR spectrum of sample; solvent: DMSO-d6

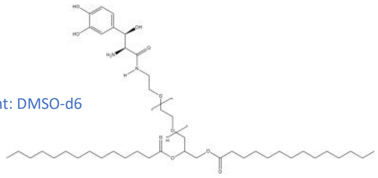

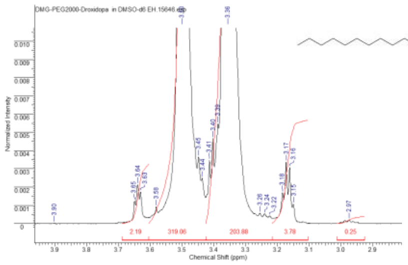

DMG-PEG2000-Norepinephrine

Zoom - H-NMR spectrum of sample; solvent: DMSO-d6

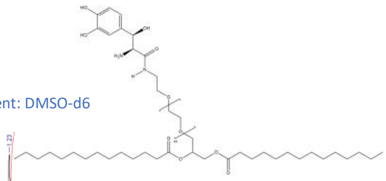

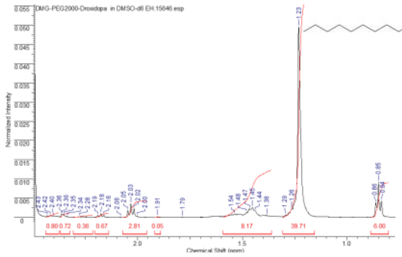

DMG-PEG2000-Norepinephrine

Zoom - H-NMR spectrum of sample; solvent: DMSO-d6

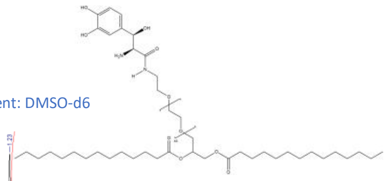

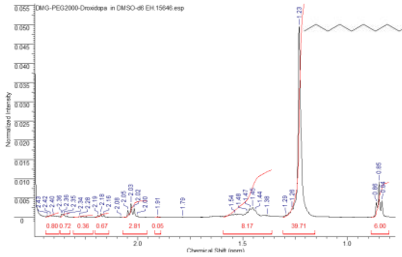

DMG-PEG2000-Norepinephrine

Zoom - H-NMR spectrum of sample; solvent: DMSO-d6

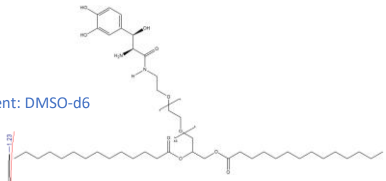

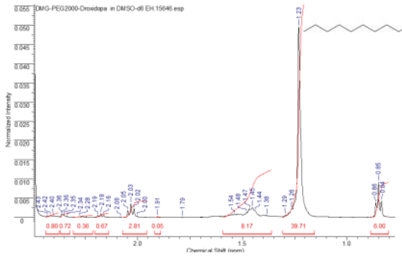

DMG-PEG2000-Norepinephrine

Proposed 1H-NMR assignment of main peaks of sample:

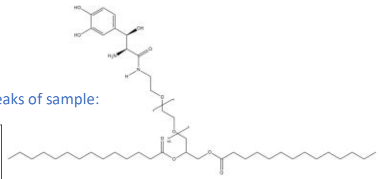

^{1}HNMR Chemical shifts (ppm)	Functional group	Structure
0.85	CH_3-	Aliphatic CH_3 CH_3-$(CH_2)_n$
1.23	-$(CH_2)_n$	Aliphatic -CH_2- CH_3-$(CH_2)_n$
1.45	-CH_2-	-CH_2-CH_2-COO
**2.03-2.40**	**-CH_2-**	CH_2-bonded to: -CH_2-COO/**-CH_2-NH-**
**3.17-4.59**	**-CH_2-/CH**	**-CH_2-O-/-CH-OH/-CH_2-CH-NH_2**
**7.00-8.50**	**-CH-**	**Aromatic ring**

S4. ^{1}H NMR of DMG-PEG2000-NH2-Norephiephrine

DMG-PEG2000-Nicotine

H-NMR spectrum of sample; solvent: DMSO-d6

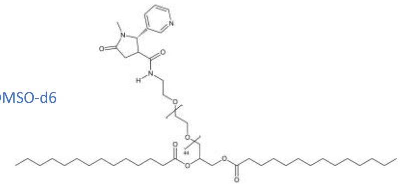

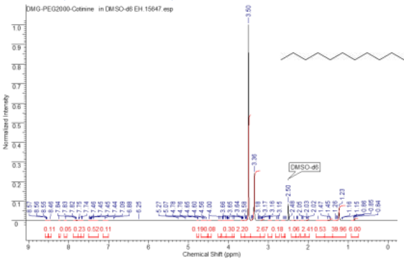

DMG-PEG2000-Nicotine

Zoom - H-NMR spectrum of sample; solvent: DMSO-d6

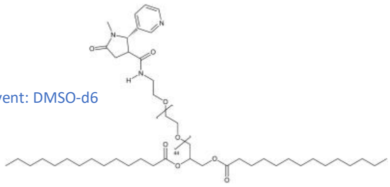

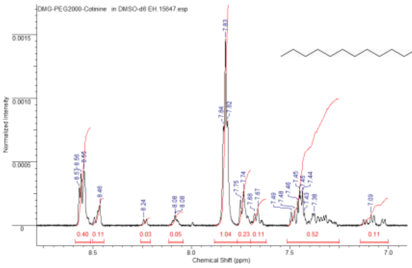

DMG-PEG2000-Nicotine

Zoom - H-NMR spectrum of sample; solvent: DMSO-d6

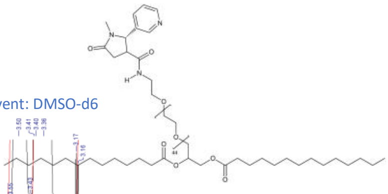

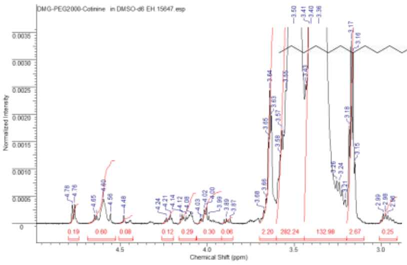

DMG-PEG2000-Nicotine

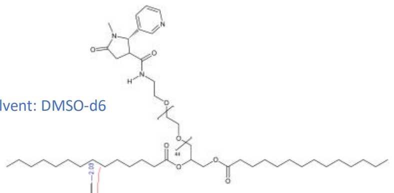

Zoom - H-NMR spectrum of sample; solvent: DMSO-d6

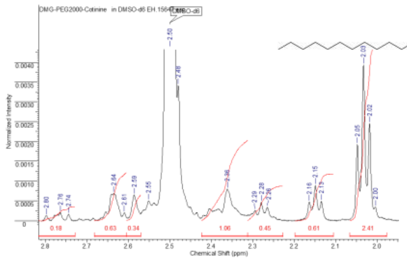

DMG-PEG2000-Nicotine

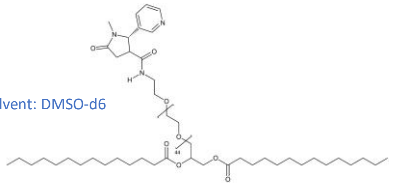

Zoom - H-NMR spectrum of sample; solvent: DMSO-d6

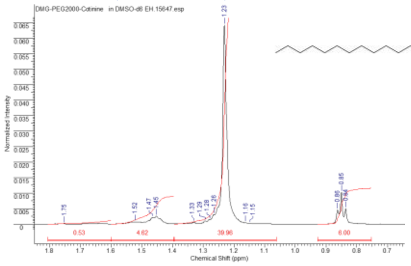

DMG-PEG2000-Nicotine

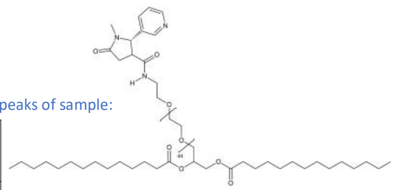

Proposed 1H-NMR assignment of main peaks of sample:

^{1}HNMR Chemical shifts (ppm)	Functional group	Structure
0.85	CH_3-	Aliphatic CH_3 $\underline{CH_3}$-$(CH_2)_n$
1.23	-$(CH_2)_n$	Aliphatic -CH_2- CH_3-$\underline{(CH_2)_n}$
1.45	-CH_2-	-$\underline{CH_2}$-CH_2-COO
**2.03-2.80**	**-CH_2-/probably CH_3 region**	CH_2-bonded to -$\underline{CH_2}$-COO/**probably $\underline{CH_3}$-N region**
**3.10-4.70**	**-CH_2-/-CH-**	**-$\underline{CH_2}$-O-/-$\underline{CH_2}$-NH-/Aliphatic ring**
**7.00-8.50**	**-CH-**	**Aromatic ring**

S5. ^{1}H NMR of DMG-PEG2000-NH2-Nicotine

DMG-PEG2000-Cocaine

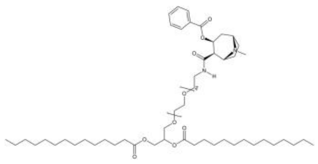

H-NMR spectrum of sample; solvent: DMSO-d6

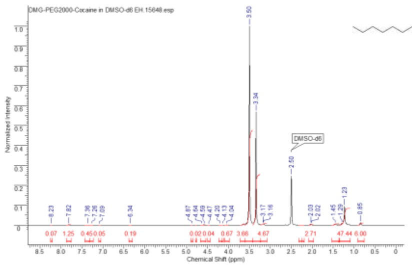

DMG-PEG2000-Cocaine

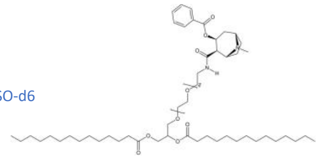

Zoom - H-NMR spectrum of sample; solvent: DMSO-d6

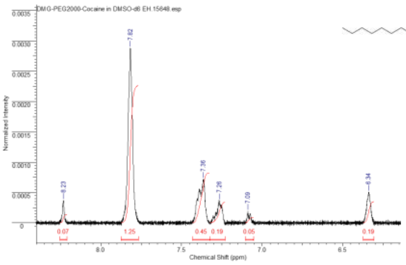

DMG-PEG2000-Cocaine

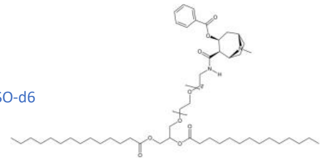

Zoom - H-NMR spectrum of sample; solvent: DMSO-d6

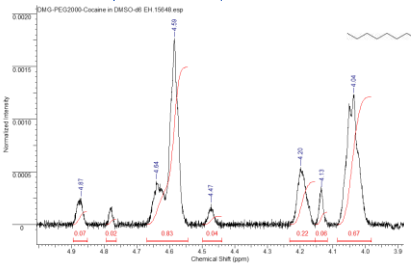

DMG-PEG2000-Cocaine

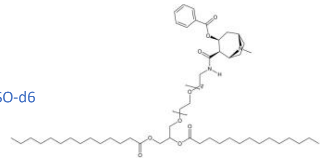

Zoom - H-NMR spectrum of sample; solvent: DMSO-d6

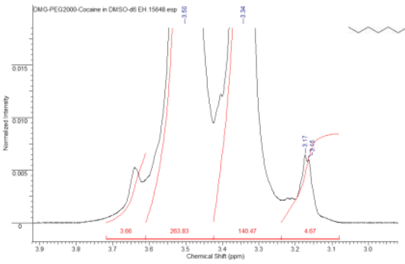

DMG-PEG2000-Cocaine

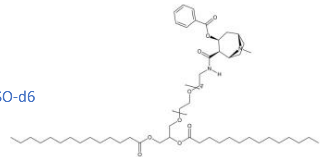

Zoom - H-NMR spectrum of sample; solvent: DMSO-d6

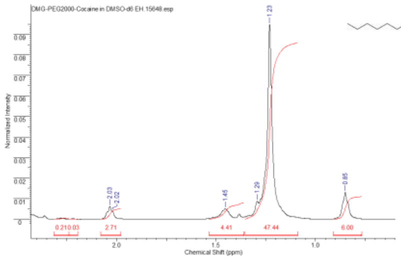

DMG-PEG2000-Cocaine

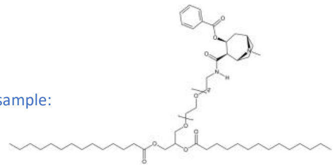

Proposed 1H-NMR assignment of main peaks of sample:

^{1}HNMR Chemical shifts (ppm)	Functional group	Structure
0.85	CH_3-	Aliphatic CH_3 $\underline{CH_3}$-$(CH_2)_n$
1.23	-$(CH_2)_n$	Aliphatic -CH_2- CH_3-$\underline{(CH_2)_n}$
1.45	-CH_2-	-$\underline{CH_2}$-CH_2-COO
**2.00-2.70**	**-CH_2-**	CH_2-bonded to -$\underline{CH_2}$-COO/**-$\underline{CH_2}$-NH-/ probably ring CH_2**
**3.17-3.64**	**-CH_2-**	**-$\underline{CH_2}$-O-/probably ring $\underline{CH}$-N**
**7.00-8.50**	**-CH-**	**Aromatic ring**

S6. ^{1}H NMR of DMG-PEG2000-NH2-Cocaine

DMG-PEG2000-Acetylcholine

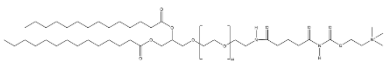

H-NMR spectrum of sample; solvent: DMSO-d6

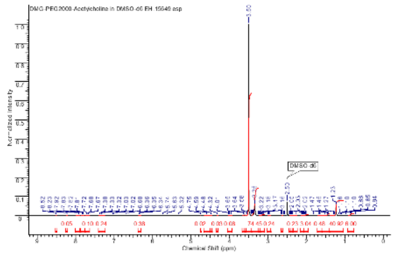

DMG-PEG2000-Acetylcholine

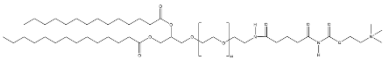

Zoom - H-NMR spectrum of sample; solvent: DMSO-d6

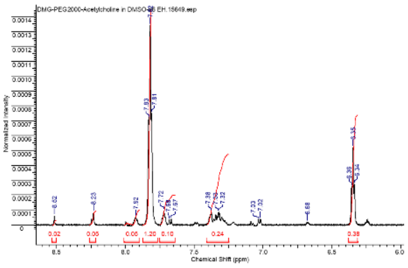

DMG-PEG2000-Acetylcholine

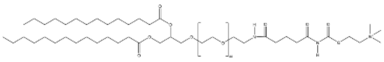

Zoom - H-NMR spectrum of sample; solvent: DMSO-d6

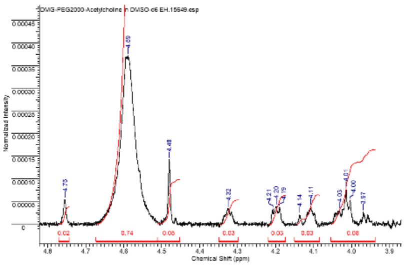

DMG-PEG2000-Acetylcholine

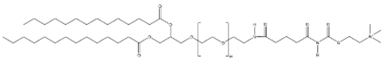

Zoom - H-NMR spectrum of sample; solvent: DMSO-d6

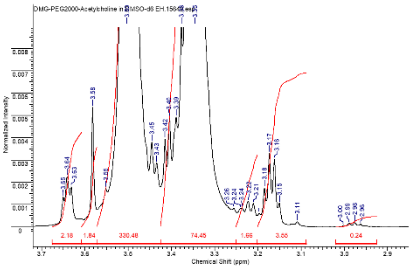

DMG-PEG2000-Acetylcholine

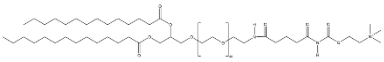

Zoom - H-NMR spectrum of sample; solvent: DMSO-d6

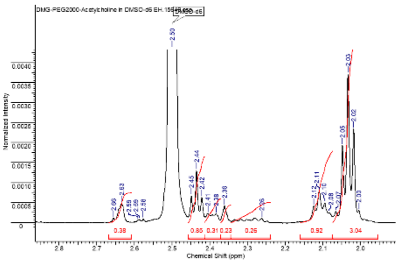

DMG-PEG2000-Acetylcholine

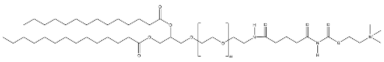

Zoom - H-NMR spectrum of sample; solvent: DMSO-d6

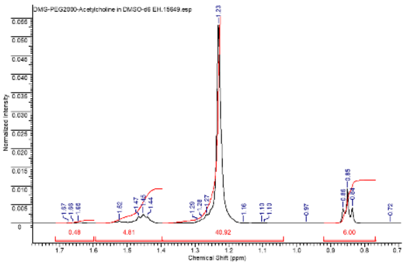

DMG-PEG2000-Acetylcholine

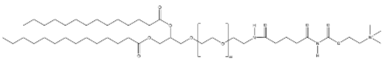

H-NMR spectrum of sample; solvent: DMSO-d6+dropD2O

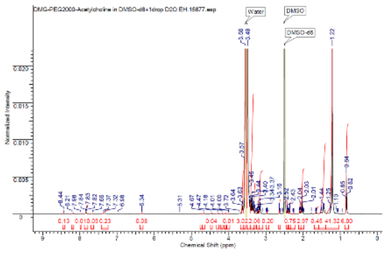

DMG-PEG2000-Acetylcholine

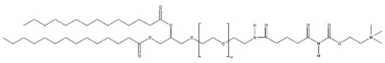

H-NMR spectrum of sample; solvent: DMSO-d6+dropD2O

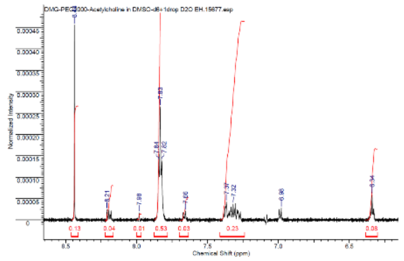

DMG-PEG2000-Acetylcholine

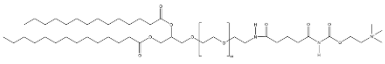

Zoom - H-NMR spectrum of sample; solvent: DMSO-d6+dropD2O

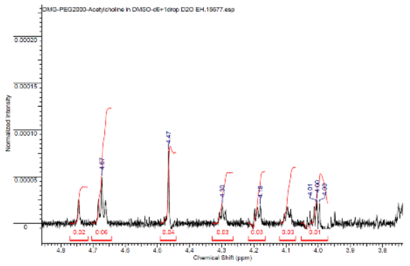

DMG-PEG2000-Acetylcholine

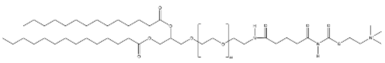

Zoom - H-NMR spectrum of sample; solvent: DMSO-d6+dropD2O

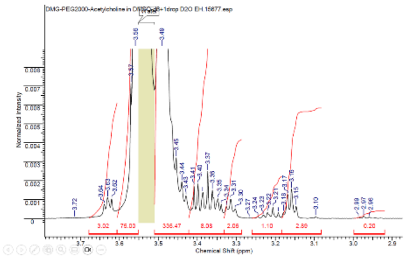

DMG-PEG2000-Acetylcholine

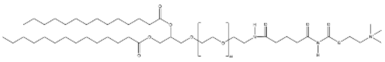

Zoom - H-NMR spectrum of sample; solvent: DMSO-d6+dropD2O

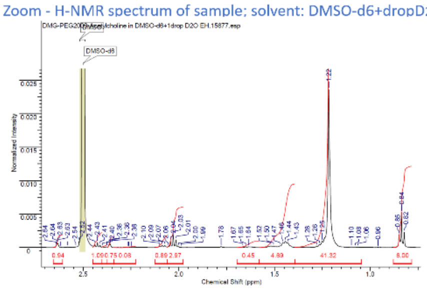

DMG-PEG2000-Acetylcholine

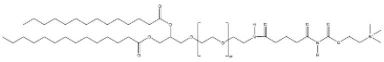

Comparison of H-NMR spectrum of sample; solvent: DMSO-d6+dropD_2O vs. DMSO-d6

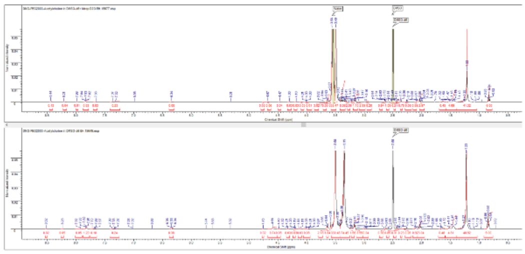

DMG-PEG2000-Acetylcholine

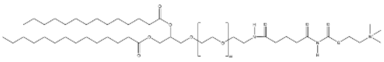

Zoom of comparison of H-NMR spectrum of sample; solvent: DMSO-d6+dropD_2O vs. DMSO-d6

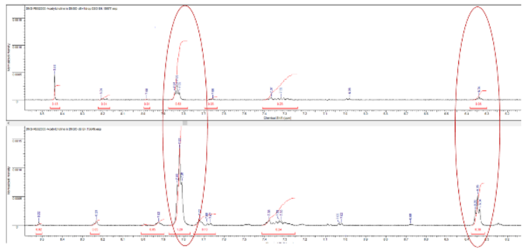

DMG-PEG2000-Acetylcholine

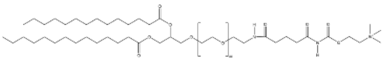

Zoom of comparison of H-NMR spectrum of sample; solvent: DMSO-d6+dropD_2O vs. DMSO-d6

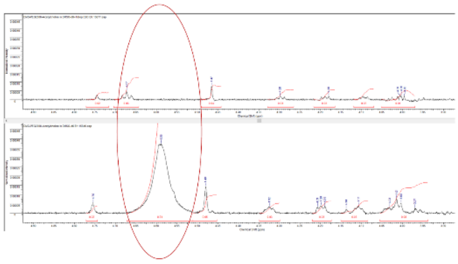

DMG-PEG2000-Acetylcholine

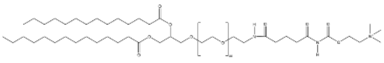

Zoom of comparison of H-NMR spectrum of sample; solvent: DMSO-d6+dropD_2O vs. DMSO-d6

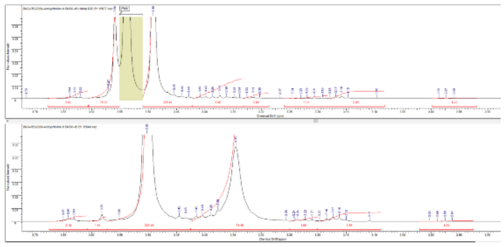

DMG-PEG2000-Acetylcholine

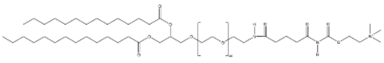

Zoom of comparison of H-NMR spectrum of sample; solvent: DMSO-d6+dropD_2O vs. DMSO-d6

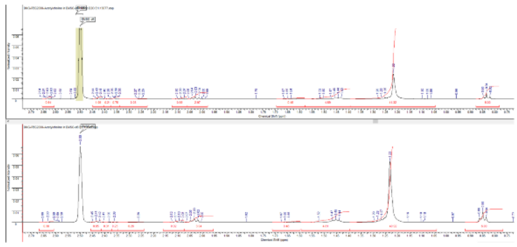

DMG-PEG2000-Acetylcholine

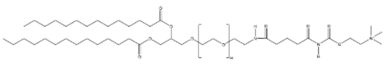

Proposed 1H-NMR assignment of main peaks of sample:

¹HNMR Chemical shifts (ppm)	Functional group	Structure
0.85	CH_3-	Aliphatic CH_3 CH_3-$(CH_2)_n$
1.23	-$(CH_2)_n$	Aliphatic -CH_2- CH_3-$(CH_2)_n$
1.45	-CH_2-	-CH_2-CH_2-COO
2.03-2.70	-CH_2-	CH_2-bonded to -CH_2-COO/-CH_2-NH-/CH_2-CH_2-COO
3.17-4.80	-CH_2-/CH_3	-CH_2-O-/-CH_2-NH-/-CH_3-N^+/-CH_2-N^+
7.00-8.50	-NH-	-CO-NH-CO-

¹HNMR Chemical shifts (ppm)	Functional group	Structure
7.8	-NH-	-CO-NH-CO-
6.3	-NH2-	-R-CO-NH2- (from unconjugated carbachol)
4.6	-NH-	-CH_2-NH-

S7. ¹H NMR of DMG-PEG2000-NH2-Acetylcholine

DMG-PEG2000-Memantine

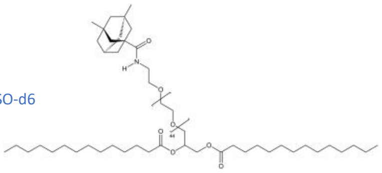

H-NMR spectrum of sample; solvent: DMSO-d6

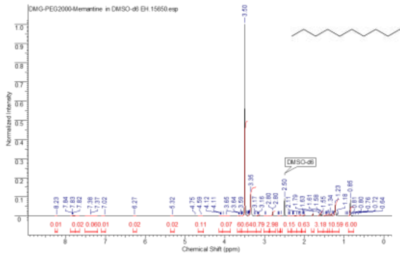

DMG-PEG2000-Memantine

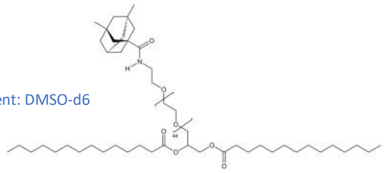

Zoom - H-NMR spectrum of sample; solvent: DMSO-d6

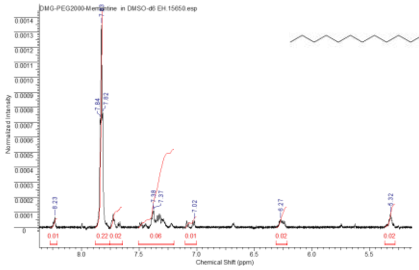

DMG-PEG2000-Memantine

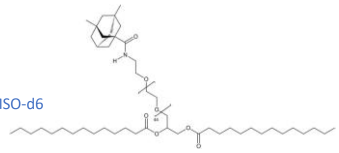

Zoom - H-NMR spectrum of sample; solvent: DMSO-d6

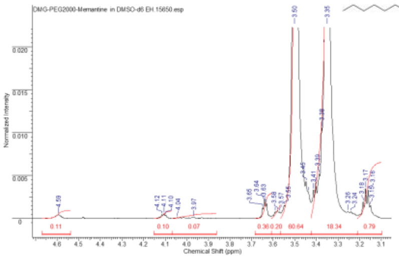

DMG-PEG2000-Memantine

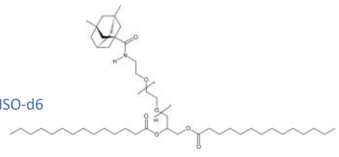

Zoom - H-NMR spectrum of sample; solvent: DMSO-d6

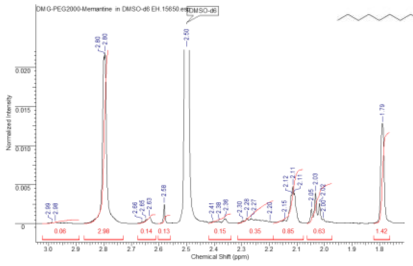

DMG-PEG2000-Memantine

Zoom - H-NMR spectrum of sample; solvent: DMSO-d6

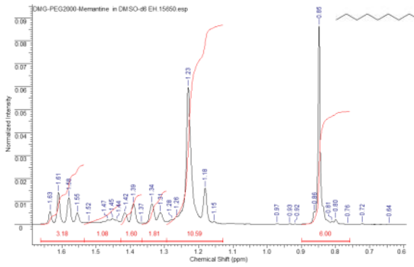

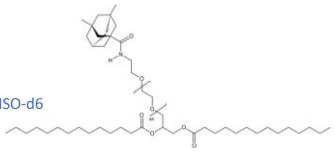

DMG-PEG2000-Memantine

Proposed 1H-NMR assignment of main peaks of sample:

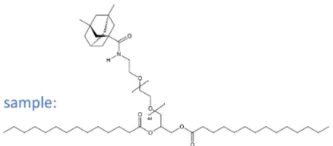

¹HNMR Chemical shifts (ppm)	Functional group	Structure
**0.85**	**CH_3-**	**Aliphatic CH_3 CH_3-$(CH_2)_n$/CH_3 ring**
1.23	-$(CH_2)_n$	Aliphatic -CH_2- CH_3-$(CH_2)_n$
**1.34-1.79**	**-CH_2-/-CH-**	-CH_2-CH_2-COO/ **probably Aliphatic ring**
**2.03-2.90**	**-CH_2-**	CH_2-bonded to -CH_2-COO/**-CH_2-NH-**
3.17-3.64	-CH_2-	-CH_2-O-

S8. ¹H NMR of DMG-PEG2000-NH2-Memantine

DMG-PEG2000-Methylphenidate

H-NMR spectrum of sample; solvent: DMSO-d6

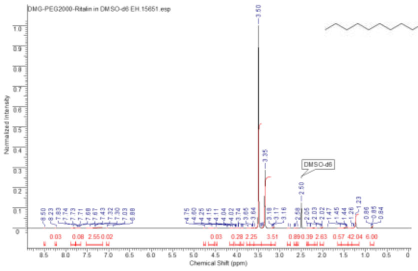

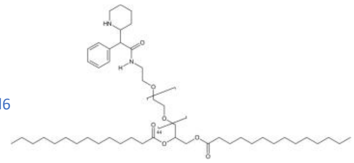

DMG-PEG2000-Methylphenidate

Zoom - H-NMR spectrum of sample; solvent: DMSO-d6

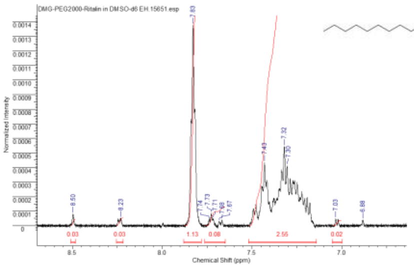

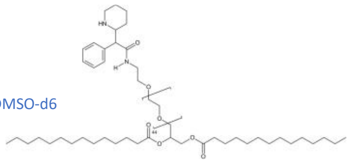

DMG-PEG2000-Methylphenidate

Zoom - H-NMR spectrum of sample; solvent: DMSO-d6

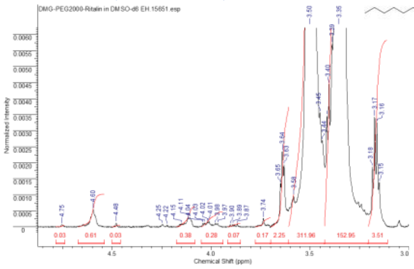

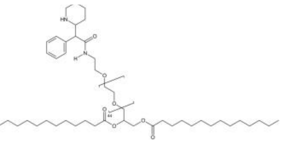

DMG-PEG2000-Methylphenidate

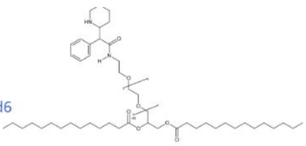

Zoom - H-NMR spectrum of sample; solvent: DMSO-d6

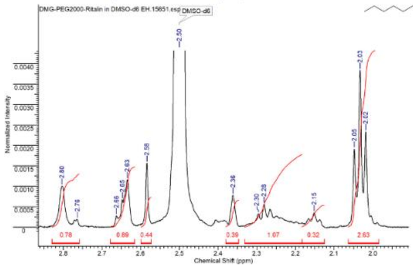

DMG-PEG2000-Methylphenidate

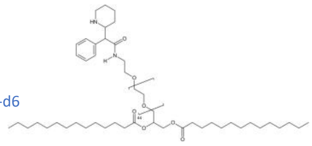

Zoom - H-NMR spectrum of sample; solvent: DMSO-d6

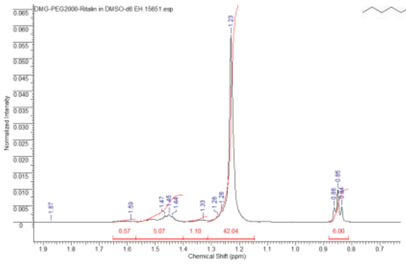

DMG-PEG2000-Methylphenidate

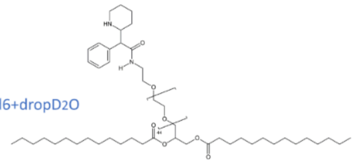

H-NMR spectrum of sample; solvent: DMSO-d6+dropD2O

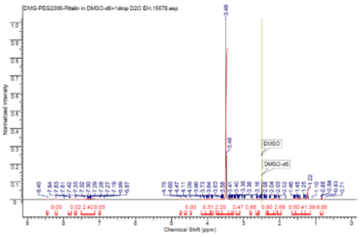

DMG-PEG2000-Methylphenidate

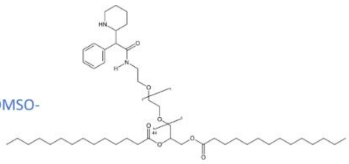

Zoom - H-NMR spectrum of sample; solvent: DMSO-d6+dropD2O

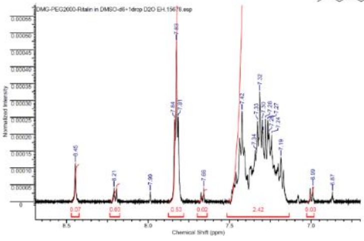

DMG-PEG2000-Methylphenidate

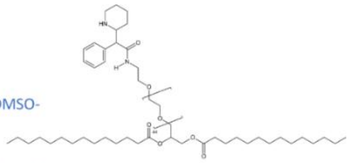

Zoom - H-NMR spectrum of sample; solvent: DMSO-d6+dropD2O

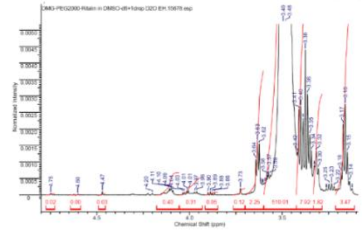

DMG-PEG2000-Methylphenidate

Zoom - H-NMR spectrum of sample; solvent: DMSO-d6+dropD2O

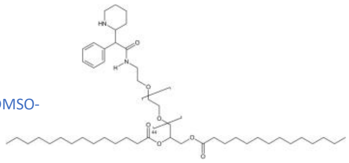

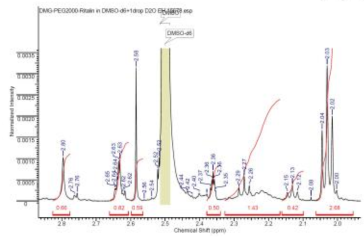

DMG-PEG2000-Methylphenidate

Zoom - H-NMR spectrum of sample; solvent: DMSO-d6+dropD2O

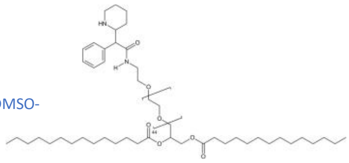

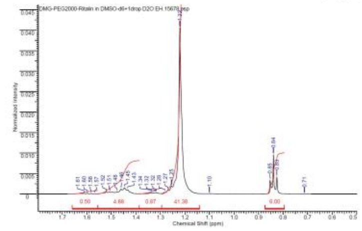

DMG-PEG2000-Methylphenidate

Comparison of H-NMR spectrum of sample; solvent: DMSO-d6+dropD2O vs. DMSO-d6

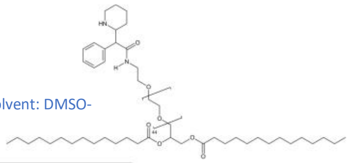

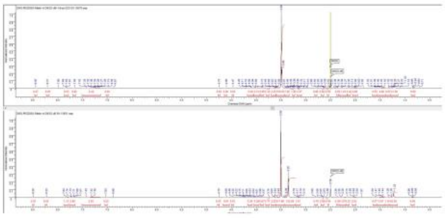

DMG-PEG2000-Methylphenidate

Zoom of comparison of H-NMR spectrum of sample; solvent: DMSO-d6+dropD2O vs. DMSO-d6

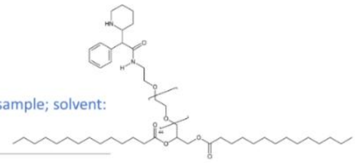

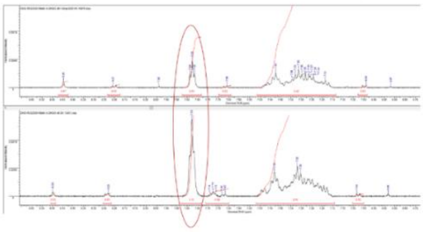

DMG-PEG2000-Methylphenidate

Zoom of comparison of H-NMR spectrum of sample; solvent: DMSO-d6+dropD2O vs. DMSO-d6

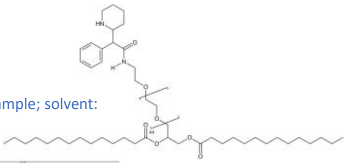

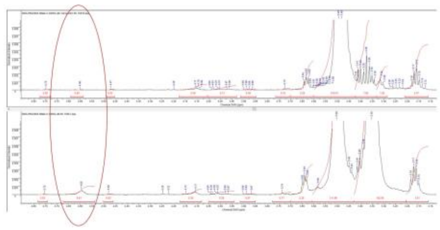

DMG-PEG2000-Methylphenidate

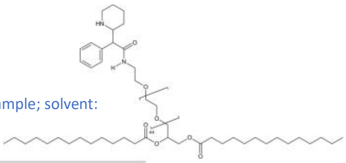

Zoom of comparison of H-NMR spectrum of sample; solvent: DMSO-d6+dropD2O vs. DMSO-d6

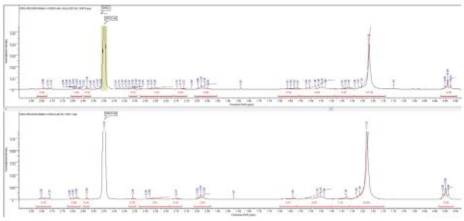

DMG-PEG2000-Methylphenidate

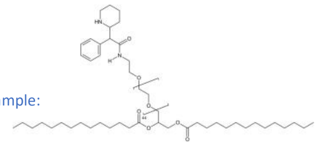

Proposed 1H-NMR assignment of main peaks of sample:

^{1}HNMR Chemical shifts (ppm)	Functional group	Structure
0.85	CH_3-	Aliphatic CH_3 CH_3-$(CH_2)_n$
1.23	-$(CH_2)_n$	Aliphatic -CH_2- CH_3-$(CH_2)_n$
1.45	-CH_2-	-CH_2-CH_2-COO
**2.03-2.80**	**-CH_2-**	CH_2-bonded to -CH_2-COO/**-CH_2-NH- /probably Aliphatic ring**
**3.17-4.60**	**-CH_2-/-CH-**	-CH_2-O-/**-CH- bridge**
**7.00-8.50**	**-CH-**	**Aromatic ring**

^{1}HNMR Chemical shifts (ppm)	Functional group	Structure
4.6	-NH-	-CH_2-NH-
7.83	-NH-	-NH- In aliphatic ring

S9. ^{1}H NMR of DMG-PEG2000-NH2-Methylphenidate
